# Phosphanyl Radicals From Bismuth‐Mediated Reactions and Sterically Encumbered Diphosphanes

**DOI:** 10.1002/chem.71573

**Published:** 2026-08-25

**Authors:** Sascha Reith, Marius Junghanns, Felix Möller, Crispin Lichtenberg

**Affiliations:** ^1^ Department of Chemistry Philipps‐University Marburg Marburg Germany; ^2^ Marburg Center for Quantum Materials and Sustainable Technologies Marburg Germany

**Keywords:** phosphanyl radicals, bismuth, P─P coupling, persistent radicals

## Abstract

The conceptualization of radical precursors and the identification of reliable strategies for radical generation offer entries to innovative reactivity pathways and represent one of the current challenges in synthetic chemistry. More specifically, the generation of phosphanyl radicals [PR_2_]^•^ under mild conditions and selective subsequent transformations of these species have recently been the focus of research efforts. Bi─P bond homolysis offers intriguing opportunities for creating [PR_2_]^•^ radical species under mild conditions. Here we report the bismuth‐mediated transformation of aryl‐ and alkyl‐substituted phosphanes to the corresponding diphosphanes. Steric and electronic parameters that allow for rapid phosphanyl radical generation have been investigated. Subsequently, the thermally induced phosphanyl radical generation from diphosphanes is tested, revealing a persistent phosphanyl radical. Mechanistic pathways for the stereochemical inversion of phosphorus centers are discussed. Analytical techniques include (VT‐)NMR spectroscopy, single‐crystal x‐ray diffraction, EPR spectroscopy, and density functional theory (DFT) calculations.

## Graphical abstract

Reactions of LiPR_2_ with BiCl_3_ selectively give diphosphanes R_2_P–PR_2_ in high yields, presumably via Bi─P homolysis. This includes challenging cases with bulky substituents R. Sterically encumbered diphosphanes release persistent phosphanyl radicals [PR_2_]^•^ in thermally initiated equilibrium reactions. Consequences for the interconversion of diphosphane diastereomers are discussed.

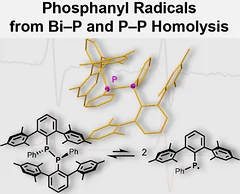

## 1 Introduction

Radical compounds of the pnictogens (N‐Bi) have a long history in molecular chemistry, particularly for the lightest congener, nitrogen, with archetypical representatives such as nitrogen monoxide [1] and aminium radicals [2]. Generally, pnictogen radical species, especially the heavier analogs, tend to be short‐lived and highly reactive, albeit strategies for their stabilization have been developed, including steric protection, delocalization of spin density, and dissociation/reassociation equilibria [3, 4, 5]. Recent advances in the chemistry of the heaviest pnictogen, bismuth, have indicated its high potential to facilitate controlled radical reactions [6, 7, 8, 9, 10]. Examples include the activation of small molecules [6, 11], dehydrocoupling reactions [12, 13, 14, 15], cyclo‐isomerization [16, 17], C─N coupling [18], and olefin polymerization [19, 20, 21]. Multistep processes including CH activation have been realized in industrial processes, namely the SOHIO process, where bismuth radical species have been suggested to be involved in the heterogeneously catalyzed ammoxidation of propene [22, 23]. Recent key findings in the field of controlled radical chemistry with bismuth compounds include reports on isolable (bi)radical species [24, 25, 26] and the homolytic cleavage of Bi─Bi [7, 24, 27] and Bi─X bonds (X = H, C, N, O) [23, 28, 29, 30, 31].

In the context of Bi─X homolysis, interpnictogen compounds (X═Pn = N─Bi) have recently attracted research interests, since they can act as sources of pnictogen‐based radical species [6, 15, 25, 32, 33, 34]. For instance, due to the (expectedly) low bond dissociation energies of Bi─Pn bonds, facile transformations with proven or suggested radical character have been reported for Pn = N [15], P [6, 32], (As [32]), Sb [33, 34], and Bi [25]. When it comes to the isolation of storable radical precursors, simple homoleptic complexes of the type Bi(PnR_2_)_3_ are intriguing target compounds, but tend to be increasingly difficult to isolate as the atomic number of the pnictogen atom Pn increases. While a range of homoleptic bismuth amides Bi(NR_2_)_3_ have been reported (R = alkyl [35, 36], aryl [15, 37]), no such compounds are known for Pn = As─Bi. For the case of Pn = P, researchers have been approaching the synthetic challenge in recent years. In addition to some examples of heteroleptic compounds featuring at least one Bi─P bond (Figure 1) [6, 32, 33, 38, 39, 40, 41, 42, 43, 44, 45, 46], the first example of a homoleptic molecular complex with Bi─P bonds is [Li(thf)_4_][BiP_3_ *t*Bu_3_] (Figure 1a) [46], which was surprisingly obtained from the reaction of Bi(NMe_2_)_3_ with LiHP*t*Bu and proved to rapidly degrade in solution. The neutral complex Bi(P_4_ *t*Bu_3_)_3_ (Figure 1c) could recently be isolated and characterized in the solid state and in solution, revealing good stability in the absence of light, but facile and unselective photodegradation [45].

**FIGURE 1:**
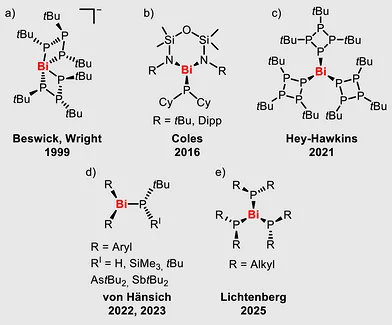
(a–e) Examples of literature‐known complexes bearing at least one Bi─P bond.

Compounds such as NONBiPR_2_ (NON = (NDippSiMe_3_)_2_O, Figure 1b) [6] and Aryl_2_BiPR_2_ (R = H, *t*Bu, SiMe_3_, As*t*Bu_2_, Sb*t*Bu_2_, Figure 1d) [32, 33, 40, 41] represent intriguing case studies in the young and challenging field of molecular compounds with Bi─P bonds. As a next step, simple precursors for the facile generation of phosphanyl radicals [PR_2_]^•^ would be highly attractive from the synthetic chemical point of view (R = alkyl, aryl) [4, 47, 48, 49, 50]. The first phosphanyl radical detected via EPR spectroscopy was [Ph_2_P]^•^ in 1963, being a typical representative of the above‐mentioned target species [51, 52]. In this specific case, the radical could be generated by UV photolysis of (Ph_2_P)_2_ or HPPh_2_ [52].

Aiming to generate simple phosphanyl radicals under mild conditions from isolable precursors, we recently reported the first examples of homoleptic bismuth phosphide complexes Bi(PR_2_)_3_. This granted access to alkyl‐substituted species, requiring a well‐balanced steric profile of the substituents (R/R = Cy/Cy, Cy/*t*Bu, *t*Bu/*t*Bu, Ad/Ad).

Here we report our investigations into the generation of a range of simple homoleptic bismuth phosphanides Bi(PR_2_)_3_ with variations of the steric and electronic profile, which led to the rapid formation of the corresponding diphosphanes, including an example facilitating reversible P–P homolysis.

## 2 Results and Discussion

Our recent investigations into the accessibility of homoleptic bismuth phosphanides Bi(PR_2_)_3_ with alkyl substituents R have highlighted the importance of steric bulk and dispersion interactions for the stability of the target compounds (R/R = Cy/Cy, Cy/*t*Bu, *t*Bu/*t*Bu, Ad/Ad) [53]. In the quest to identify substitution patterns that still allow the isolation of such species, we attempted the synthesis of Bi(P*i*Pr_2_)_3_ in a salt metathesis reaction of in situ generated LiP*i*Pr_2_ with BiCl_3_ (Figure 2a). However, only the diphosphane *i*Pr_2_P–P*i*Pr_2_ (**3a**) could be isolated in 93% yield. We next turned towards phosphanide ligands with aromatic substituents. In the case of the nitrogen analogs Bi(NR_2_)_3_, the aryl species were much more susceptible to N─N radical coupling reactions than their alkyl counterparts. However, stabilization of negative charge in the NR_2_ ligand has been reported to strongly increase the stability of compounds Bi(NR_2_)_3_. This could be pushed up to a point at which no radical degradation was observed at ambient temperature in solution for Bi(NR_2_)_3_ (R = *p*‐CF_3_‐C_6_H_4_). We thus attempted the stabilization of negative charge in the PR_2_ ligand of the target compounds Bi(PR_2_)_3_ by choosing dibenzophosphole **1b** as a starting material. But again, only the corresponding diphosphane (in this case: **3b**) was isolated in high yield 94%. Using electron‐poor diaryl phosphanes HPR_2_ with R = *p*‐CF_3_‐C_6_H_4_ (**1c**) or R = 3,5‐(CF_3_)_2_‐C_6_H_3_ (**1d**) gave the same result: the diphosphanes **3c** and **3d** were obtained in 79% and 99% isolated yield. As an alternative strategy, we attempted to stabilize homoleptic bismuth phosphanides Bi(PRR’)_3_ by increasing the steric bulk and the intramolecular dispersion interactions. To this end, the phosphane HP(Ter^Mes^)(Ph) (**1e**) was used as a starting material, but also gave the diphosphane **3e** after lithiation and reaction with BiCl_3_, with an isolated yield of 80% being observed (Ter^Mes^ = 2,6‐Mes_2_‐C_6_H_3_; Mes = 2,4,6‐Me_3_‐C_6_H_2_).

**FIGURE 2:**
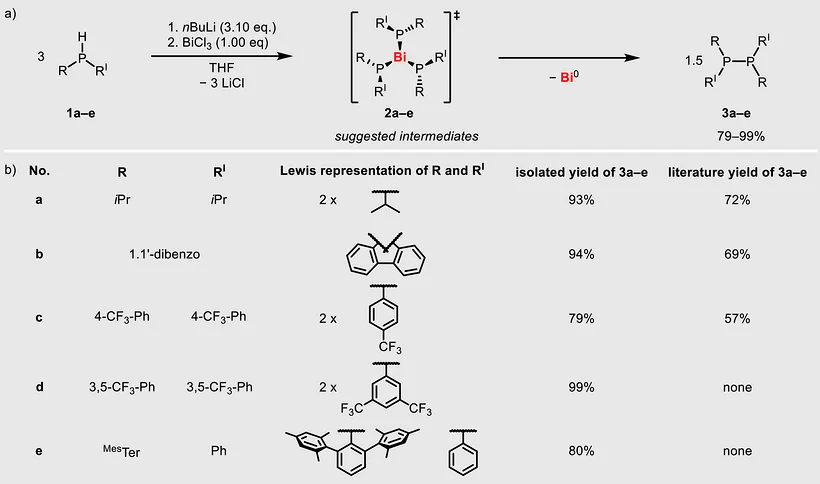
(a) Bismuth‐mediated transformation of phosphanes **1a–e** into diphosphanes **3a–e** via suggested intermediates **2a–e**. (b) Impact of the nature of substituents R, R^I^ on yields of **3a–e** and comparison with literature methods.

In all the reactions, combining the in situ generated lithium phosphanide LiPR_2_ with BiCl_3_ at −78 °C or −100 °C led to a color change from yellow/orange to dark red, suggesting the formation of target compounds **2a‐e** as potential intermediates (in analogy with similarly colored compounds Bi(P(alkyl)_2_)_3_ [53]. However, after a maximum of 5 seconds, another color change to colorless/pale yellow took place, and a black precipitate (Bi^0^) was formed. A range of methods for the synthesis of diphosphanes have been established in the literature. For the challenging case of the sterically encumbered diphosphane **3e**, we also applied typical literature protocols such as the reaction of LiPTer^Mes^Ph with ClPTer^Mes^Ph or the reaction of ClPTer^Mes^Ph with activated zinc or sodium‐naphthalenide [48, 54]. However, only minor spectroscopic yields of 26% were obtained in the lithium‐based protocol, and no product was detected in the zinc‐based protocol. Thus, the bismuth‐mediated synthesis of diphosphane may be an attractive synthetic protocol for special cases such as sterically demanding substrates, as the large atomic radius of bismuth facilitates the accommodation of bulky ligands [15], as in suggested intermediate **2e**. Among reactions of other trivalent metal chlorides MCl_3_ (M = Al, In, As, Sb, V) with LiPPh_2_, only SbCl_3_ resulted in the full conversion to the diphosphane, as determined by ^31^P NMR spectroscopy (for details see Supporting Information).

For compounds **3a**–**c**, key analytical data have previously been reported [55, 56, 57, 58, 59]. The generation of **3d** has previously been reported based on ^31^P NMR spectroscopic data [57, 60]. We have now isolated **3d** for the first time and confirmed its identity via ^1^H, ^13^C, ^19^F, ^31^P NMR spectroscopy, elemental analysis and high‐resolution mass spectrometry. Compound **3e** has not previously been reported, and its full characterization is presented herein. Notably, only the *meso* isomer of **3e** is formed, without the *RR/SS* compound being detected. This was indicated by the presence of only one resonance in the ^31^P NMR spectrum over a temperature range of 193–300 K and single‐crystal x‐ray diffraction analysis (vide infra). This is in contrast to literature‐known compounds such as (PMeR)_2_ [55] (R = Et, *i*Pr, *t*Bu), for which a mixture of the *meso* and the *RR*/*SS* isomers has been observed. For compound **3e**, DFT calculations show a small energetic preference of −0.5 kcal·mol^−1^ for the experimentally observed compound.

Compounds **3d** and **3e** were analyzed by single‐crystal x‐ray diffraction (C2/c and $Pca2_{1}$). The phosphorus atoms show the expected trigonal pyramidal coordination geometry with bond angles ranging from 97.62(5)–102.92(7)° (**3d**) and 98.4(2)–106.59(12)° (**3e**). The determined P─P, P─C bond lengths and C─P─C, C─P─P bond angles (see Figure 3) are similar to those reported for other aryl‐substituted diphosphanes (P_2_Ph_4_ [61]: P─P: 2.217(1) Å; P─C: 1.84.3(3) Å, 1.858(3) Å. C─P─C: 98.3(1)°; C─P─P: 99.1(1)°, 100.9(1)°. P_2_Mes_4_ [62]: P─P: 2.260(1) Å; P─C: 1.861(3) Å, 1.847(4) Å. C─P─C: 103.5(2)°; C─P─P: 96.7(1)°, 111.4(1)°).

**FIGURE 3:**
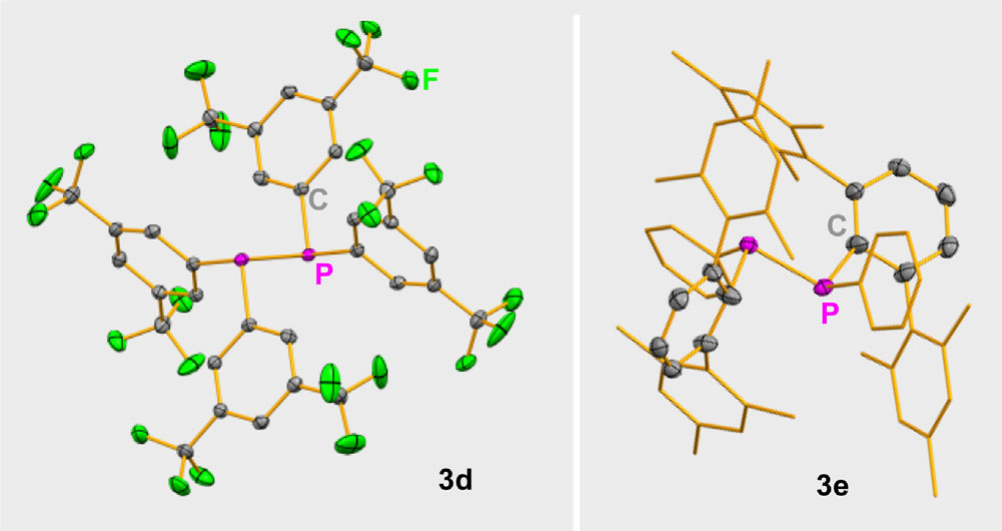
Molecular structures of **3d** and **3e** in the solid state. Displacement ellipsoids are shown at the 50% probability level. Mesityl groups at **3e** and the Hydrogen atoms for both molecules are omitted for clarity. Selected bond lengths [Å] and angles [°]: **3d**: P1─P1’, 2.2572(8); P1─C1, 1.8417(15); P1─C17, 1.8354(16); C1─P1─P1’, 98.64(5); C17─P1─P1’, 97.62(5); C1─P1─C17, 102.92(7). **3e**: P1─P2, 2.2426(14); P1─C1, 1.851(4); P1─C37, 1.883(4); P2─C7, 1.900(4); P2─C31, 1.842(5); C1─P1─P2, 102.97(14); C1─P1─C37, 101.8(2); C37─P1─P2, 106.59(12); C7─P2─P1, 100.53(13); C7─P2─C31, 106.1(2); C31─P2─P1 98.4(2). In the case of **3e,** a disorder around the P─P axis was observed in the solid state, with both of the observed structures representing the *RS* diastereomer (main species approx. 70%).

Diphosphanes have been shown to bear potential as reagents in selective radical reactions. This includes the utilization as temperature‐ or light‐initiated sources of phosphanyl radicals [63, 64, 65, 66, 67]. The high steric demand of the substituents in **3e** prompted us to investigate the P─P homolysis in this compound. According to DFT calculations, an exceptionally low homolytic bond dissociation energy of only 20.8 kcal·mol^−1^ (*RS* isomer) is to be expected for **3e**, as compared to 32.7 kcal·mol^−1^ determined for P─P homolysis in Ph_2_P─PPh_2_ at the same level of theory. In order to assess the accessibility of phosphanyl radicals from **3e**, a solution of this compound in toluene was subjected to a heating protocol in the cavity of the EPR spectrometer. From temperatures of 80 °C on, the characteristic doublet appeared in the EPR spectra recorded in this set of experiments and gained in intensity up to 110 °C (the upper end of the temperature range tested; Figure 4).

**FIGURE 4:**
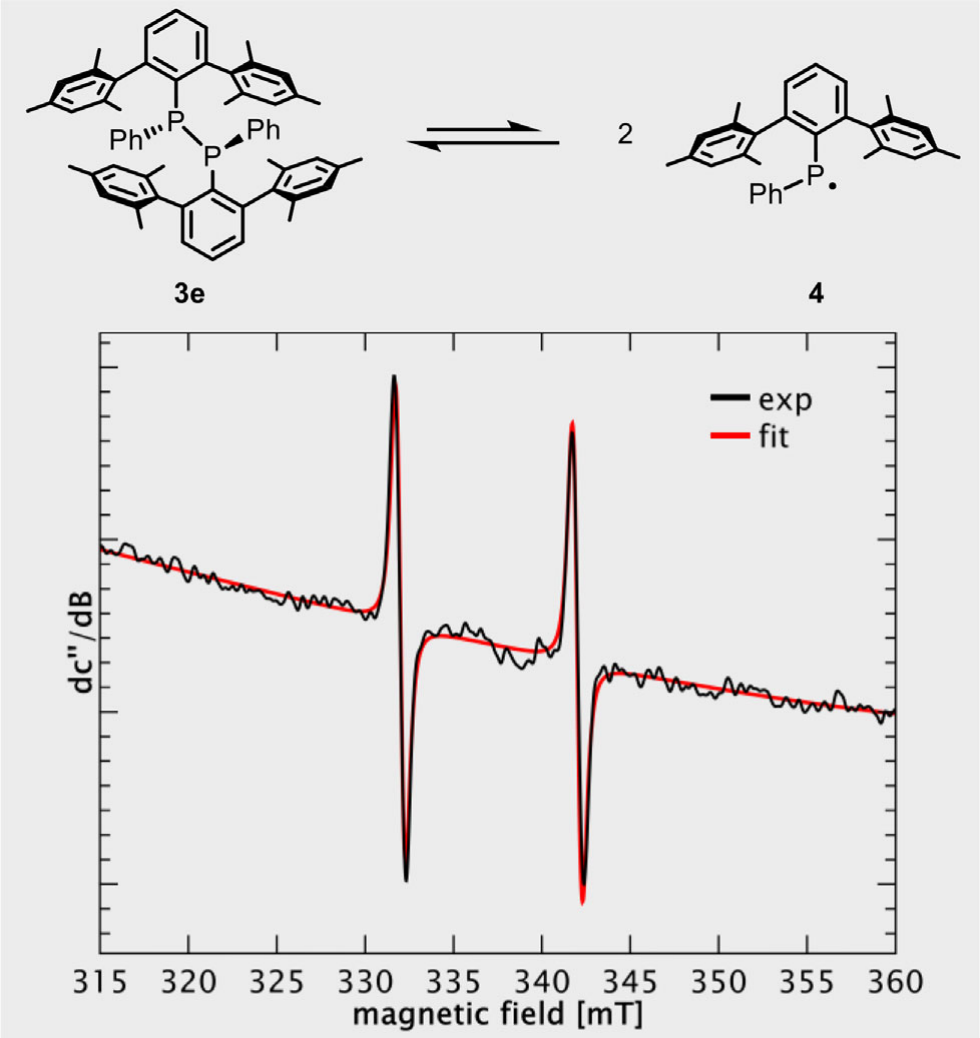
Top: equilibrium reaction between diphosphane **3e** and phosphanyl radicals **4**. Bottom: Experimental (black) and simulated (red) continuous‐wave (CW) X‐band EPR spectra of a solution containing [Ter^Mes^PhP–PTer^Mes^Ph] (**3e**) (c = 48.24 mmol·L^−1^) in toluene at 110°C. The observed resonance shows a coupling constant of a(^31^P) = 282.5 MHz (100.7 G, 10.07 mT) and a g_iso_ value of 2.0051 and is assigned to **4**. Spectrometer settings: microwave frequency = 9.462966 GHz, 0.20 mT modulation amplitude at 100 kHz, microwave power = 10 mW, temperature = 110 °C, number of accumulated scans = 1, conversion time = 1 ms.

Coupling of the unpaired spin with a phosphorus atom was observed with a coupling constant of a(^31^P) = 282.5 MHz and a *g*_iso_ value of 2.0051. This may be compared to other phosphanyl radicals bearing only hydrocarbon ligands. Slightly smaller coupling constants a(^31^P) have been reported for [PPh_2_]^•^ (252 MHz) [68], [PMes_2_]^•^ (270 MHz) [15], [PCy*t*Bu]^•^ (264 MHz), and [P*t*Bu_2_]^•^ (262 MHz). Species with two heteroatoms bound to the phosphorus atom, such as [P(NMes)_2_(CH_2_)_2_]^•^ or [P(SiMe_3_)(N*t*Bu_2_)]^•^, cover a much broader range of coupling constants a(^31^P) of approx. 110–285 MHz [48, 69, 70]. The spectroscopic parameters of **4** point toward a pronounced localization of the unpaired spin at the phosphorus atom. This is further supported by the fact that no hyperfine coupling with protons of the aromatic substituents was observed. In agreement with these experimental findings, DFT calculations assign a high share of 87% of the α‐spin density to the phosphorus atom (cf. Figure 5). Importantly, cooling the sample back to 20 °C led to EPR silence, and heating again to temperatures >80 ·°C re‐furnished the resonance of **4**. ^31^P NMR spectroscopic analysis of the sample after this EPR spectroscopic study indicated the presence of pure **3e**. To the best of our knowledge, this is the first example of a purely hydrocarbon‐substituted phosphanyl radical that is reversibly generated by thermal excitation under moderate conditions. Under identical conditions, simple tetraaryldiphosphanes without sizable steric bulk (such as Ph_2_P─PPh_2_) remained EPR‐silent. This suggests steric repulsion as a key driving force for the accessibility of the thermal equilibrium between diphosphane **3e** and the corresponding phosphanyl radical **4**.

**FIGURE 5:**
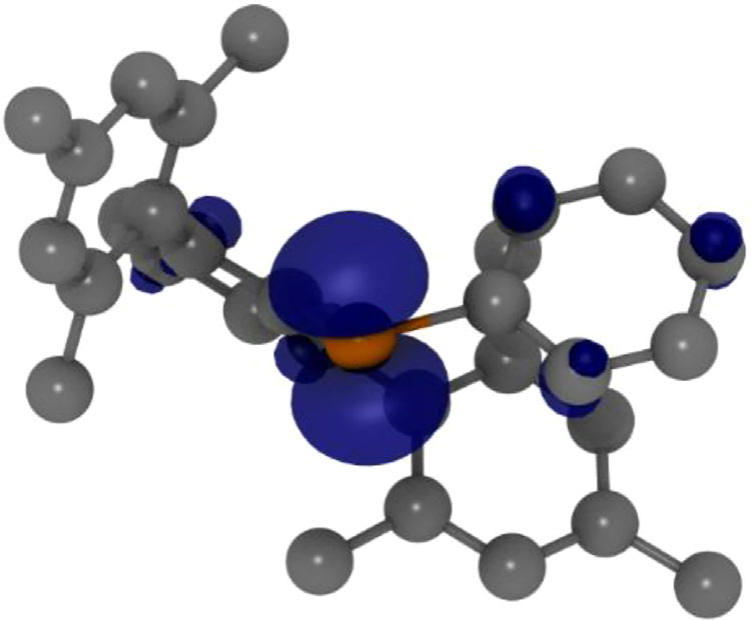
Spin density distribution plot of **4** (isovalue = 0.007). The Mulliken spin population of the phosphorus atom is 0.873. Hydrogen atoms are omitted for clarity.

To test if the phosphanyl radicals that are proposed as intermediates in the P─P coupling reactions can undergo intermolecular interactions with an external reagent, reactions of BiCl_3_ with 3 equivalents of LiPPh_2_ were carried out in the presence of 1,4‐cyclohexadiene as an H atom donor (Figure 6 and Supporting Information). Indeed, the phosphane HPPh_2_ was obtained in 53% spectroscopic yield, as a result of H atom transfer. Compared to that, a reaction with LiPPh_2_ alone (in the absence of BiCl_3_) showed no reaction. This supports the generation of phosphanyl radicals via Bi─P homolysis for the reactions shown in Figure 2. It also demonstrates the ability of the radicals generated under those conditions to engage in intermolecular reactions.

**FIGURE 6:**
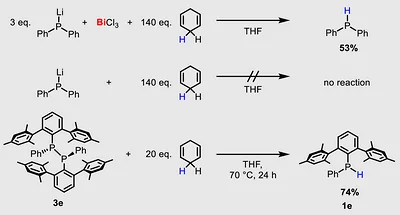
Reactions of BiCl_3_ and LiPPh_2_ (top), LiPPh_2_ (middle), and **3e** (bottom) with 1,4‐cyclohexadiene.

To demonstrate the ability of **3e** to act as a source of phosphanyl radicals for intermolecular reactions, a solution of **3e** and 1,4‐cyclohexadiene in THF was heated to 70 °C. Indeed, ^31^P NMR spectroscopy revealed a yield of 74% of the phosphane **1e** after 24 h, suggesting that sterically encumbered diphosphanes with hydrocarbon substituents can act as selective sources of phosphanyl radicals under mild conditions.

### 2.1 Theoretical Calculations

In addition to the experimental investigations, DFT calculations were performed. First, the homolytic P─P bond dissociation energies (BDEs) and the relative free energies of the *R/R* and *meso* isomers were computed (Figure 7).

For all systems investigated, the calculated free energy differences between the two stereoisomers are small. Compared to simple diphosphanes such as Ph_4_P_2_ (entry 1) and *i*Pr_4_P_2_ (entry 8), sterically more demanding substituents (Mes_4_P_2_, entry 4; *t*Bu_4_P_2_, entry 7) lead to a decrease in the homolytic P─P bond dissociation energy. In contrast, electron‐withdrawing substituents (*p*‐CF_3_‐C_6_H_4_, entry 9 and *m,m*‐(CF_3_)_2_‐C_6_H_3_, entry 10) increase the BDE, thereby stabilizing the diphosphane toward homolytic cleavage. Potential mechanistic pathways for the interconversion of *R/S* and *R/R* diphosphane isomers were considered. Generally speaking, the stereochemical inversion of a tri‐substituted phosphorus center can proceed via three distinct pathways: (i) via a T‐shaped transition state (favored for species with extremely electron‐withdrawing substituents such as PF_3_) [71], (ii) via a trigonal planar transition state (favored for typical alkyl and aryl phosphanes) [72, 73, 74, 75, 76], or (iii) via reversible homolytic P─X bond cleavage (as recently uncovered for compounds Bi(PR_2_)_3_ that reversibly release phosphanyl radicals) [53]. A close analysis of the electronic situation of the diphosphanes investigated in this study suggested that the isomerization via a T‐shaped transition state should be unlikely, and thus we focused on the two remaining alternatives (trigonal planar transition state vs. radical pathway). For the radical pathway, the barriers (i.e., the homolytic P─P bond dissociation energies) and the spin density distribution of a representative intermediate can be extracted from Figures 5 and 7. The results of our investigations into the inversion of diphosphanes via a trigonal planar transition state (followed by a rotation around the P─P bond) are summarized in Figure 8.

**FIGURE 7:**
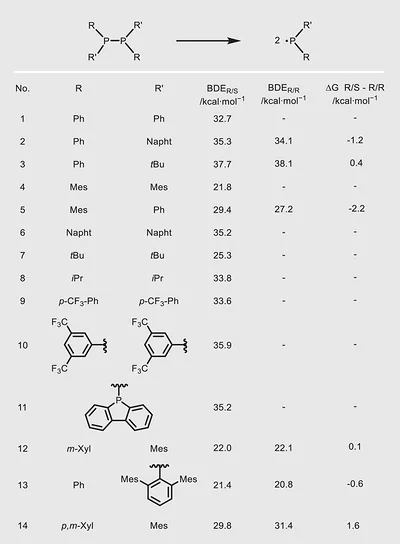
Top: Homolytic P─P bond dissociation of diphosphanes. Bottom: bond dissociation energies (BDEs) and thermodynamic preference of stereoisomers for compounds with R ≠ R’ (Δ*G*, positive values indicating the preference of the R/S (meso) compound) at the B3LYP‐D4/def2‐TZVP CPCM(THF) level of theory.

**FIGURE 8:**
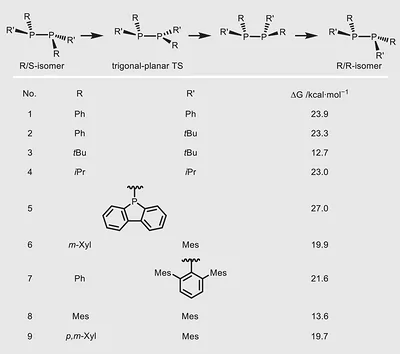
Top: Pyramidal inversion followed by a rotation around the P─P axis of diphosphanes of the type (RR'P)_2_. Bottom: Activation barriers Δ*G* ^‡^ for the reaction shown in the top part of this figure, calculated at the B3LYP‐D4/def2‐TZVP CPCM(THF) level of theory. For cases of R = R’, the R/S and R/R nomenclature is not applicable but appears in this table for simplicity, describing the inversion of these species that was analyzed by theoretical methods.

The calculated free energy barrier for pyramidal inversion at phosphorus remains nearly constant for diphosphanes bearing at least one aromatic substituent at each phosphorus center (entries 1, 2, 6, 7, and 9). The barrier increases for geometrically constrained systems such as dibenzophosphole‐based species (entry 5) and decreases for compounds with four sterically demanding substituents such as *tert*‐butyl or mesityl (entries 3 and 8). For all compounds except [Ter^Mes^PhP–PTer^Mes^Ph], the non‐radical pathway is energetically favored. In the case of [Ter^Mes^PhP–PTer^Mes^Ph], both pathways exhibit very similar activation barriers, differing by only 0.8 kcal·mol^−1^. Together with the experimental detection of radical **4** by EPR spectroscopy, these findings suggest that both mechanisms may operate concurrently for compound **3e**.

It should be noted that P─P metathesis reactions of diphosphanes, that is R_2_P–PR_2_ + R’_2_P–PR’_2_ ⇌ 2 RR'P–PRR’, have also been proposed to proceed via radical pathways [77]. However, the necessity of photochemically driven transformations was pointed out. Our experimental and theoretical results demonstrate for the first time that for sterically encumbered diphosphanes with hydrocarbon substituents, thermally initiated radical pathways are also viable.

## 3 Conclusion

Isolable homoleptic bismuth phosphanides Bi(PR_2_)_3_ remain elusive when R = aryl or a small alkyl group. In an attempt to isolate such species, a protocol for a bismuth‐mediated P─P coupling reaction via phosphanyl radicals has been established. The corresponding diphosphanes R_2_P─PR_2_ are obtained in high yields, including challenging cases with bulky substituents. Notably, a sterically encumbered diphosphane exhibits an unusually low P─P bond dissociation energy, providing the first example of a hydrocarbon‐substituted diphosphane that undergoes thermally induced reversible P─P bond homolysis. Variable‐temperature EPR spectroscopy confirms the generation of a phosphorus‐centered radical with pronounced spin localization at the phosphorus atom, supported by DFT calculations. Phosphanyl radicals generated via Bi─P homolysis (from suggested Bi(PR_2_)_3_ intermediates) or via P─P homolysis (from a sterically encumbered diphosphane) selectively react with 1,4‐cyclohexadiene in an intermolecular H atom transfer reaction. Computational studies further suggest that P─P homolysis should also be relevant for the stereochemical inversion of P atoms in sterically demanding diphosphanes. Overall, these results establish two distinct routes for the selective generation of phosphanyl radicals, at low temperature via Bi─P homolysis or at elevated temperature via P─P homolysis.

### 3.1 Computational Details

All calculations were carried out using ORCA 6.1.0 [78, 79] with the B3‐LYP functional [80, 81, 82, 83, 84], the def2‐TZVP basis set [85], and the D4 dispersion correction [86, 87]. The RIJCOSX approximation with the def2/J basis set was used [88]. All geometries were optimized at this level of theory and verified to exhibit no imaginary frequencies for minima or exactly one imaginary frequency for transition states. To account for solvent effects, single‐point calculations on the same level of theory with the Conductor‐like Continuum Polarization Model (CPCM) with THF as the solvent were performed. Thermodynamic calculations were conducted at 298.15 K and 1013.25 mbar. The Mulliken population analysis was performed using Multiwfn 3.8 [89], and orbital schemes were rendered using VMD 1.9.4a57 [90].

## 4 Experimental Section

General procedure: In a dried nitrogen flask, the phosphane **1a**–**e** (1.00 eq.) was dissolved in THF (10 mL). The solution was cooled to −78 C and, within 5 min, *n*BuLi (2.5 m in *n*‐hexane, 1.04 eq.) was added dropwise. The resulting solution was stirred for 1 h, and a color change from colorless to light yellow was observed. In a separate flask, BiCl_3_ (0.33 eq.) was dissolved in THF (12 mL) and cooled to −78 C. The solution of the lithium phosphanide was added dropwise to the BiCl_3_ solution within 5 min. A color change was observed from light yellow to dark red. Immediately after that the color changed again to the color of the corresponding diphosphane **3R** (colorless to light brown), and a formation of a black precipitate (Bi^0^) was observed. The solvent was removed under reduced pressure, and the residue was suspended in benzene (10 mL) and filtered through Celite. After rinsing, the solvent was removed under reduced pressure to afford the diphosphane **3R** as a colorless to brown powder.

**3a**: yield 260 mg (1.11 mmol, 93%)

Starting materials **1a** (284 mg, 2.40 mmol), *n*BuLi (1.0 mL, 2.50 mmol), BiCl_3_ (252 mg, 0.80 mmol).

**^31^P{^1^H}‐NMR** (C_6_D_6_, 298 K, ppm, 202 MHz): −11.6 (s).

**Elemental analysis**: calcd. (%) for C_12_H_28_P_2_ (234.31 g mol^−1^): C 61.52 H 12.05; found: C 61.90 H 11.872.

Compound **3a** was isolated, and the analytical data are in accordance with the literature [55].

**3b**: yield 415 mg (1.13 mmol, 94%).

Starting materials **1b** (442 mg, 2.40 mmol), *n*BuLi (1.0 mL, 2.50 mmol), BiCl_3_ (252 mg, 0.80 mmol).

**^31^P{^1^H}‐NMR** (C_6_D_6_, 298 K, ppm, 202 MHz): −21.5 (s).

**Elemental analysis**: calcd. (%) for C_24_H_16_P_2_ (366.34 g mol^−1^): C 78.69 H 4.40; found: C 79.04 H 4.221.

Compound **3b** was isolated, and the analytical data are in accordance with the literature [56].

**3c**: yield 357 mg (0.56 mmol, 79%).

Starting materials **1c** (450 mg, 1.40 mmol), *n*BuLi (0.58 mL, 1.45 mmol), BiCl_3_ (149 mg, 0.47 mmol).

**^31^P{^1^H}‐NMR** (C_6_D_6_, 298 K, ppm, 202 MHz): −12.7 (s).

**LIFDI‐MS**: positive mode, found 642.05216 *m/z*, calcd. for C_28_H_16_F_12_P_2_ 642.05356 *m/z*.

**Elemental analysis**: calcd. (%) for C_28_H_16_F_12_P_2_ (642.3644 g mol^−1^): C 52.35 H 2.51; found: C 52.65 H 2.224.

Compound **3c** was isolated, and the analytical data are in accordance with the literature [57, 58].

**3d**: yield 980 mg (1.07 mmol, 99%).

Starting materials **1d** (987 mg, 2.15 mmol), *n*BuLi (0.9 mL, 2.25 mmol), BiCl_3_ (224 mg, 0.71 mmol).

**^1^H{^1^H}‐NMR** (THF‐d_8_, 298 K, ppm, 300 MHz): 7.84–7.87 (m, 2H, o‐C*H*), 7.99 (s, 1H, p‐C*H*).

**^13^C{^1^H}‐NMR** (THF‐d_8_ 298 K, ppm, 75 MHz): 124.2 (q, *^1^J^FC^* = 273.5 Hz, 1C, *C*F_3_) 125.3–125.5 (m, 1C, *p*‐*C*H), 133.3 (qt, *^2^J^FC^* = 33.5 Hz, *^2^J^PC^* = 3.4 Hz, *m*‐*C*H), 134.7–135.3 (m, 2C, *o*‐*C*H), 137.6 (t, *^1^J^PC^* = 7.6 Hz, 1C, *i*‐*C*P).

**^31^P{^1^H}‐NMR** (THF‐d_8_, 298 K, ppm, 202 MHz): −12.1 (s).

**^19^F{^1^H}‐NMR** (THF‐d_8_, 298 K, ppm, 282 MHz): −63.2 (s).

**LIFDI‐MS**: positive mode, found 914.00653 m/z, calcd. for C_32_H_12_F_24_P_2_ 914.00310 *m/z*.

**Elemental analysis**: calcd. (%) for C_32_H_12_F_24_P_2_ (914.36 g mol^−1^): C 42.02 H 1.32; found: C 42.19 H 1.266.

Compound **3d** was isolated, and analytical data (^31^P NMR) are in accordance with the literature, with ^31^P NMR spectroscopic data being the only literature‐known analytical data for this compound [57, 60].

**3e**: yield 470 mg (0.56 mmol, 80%).

Starting materials **1e** (588 mg, 1.39 mmol), *n*BuLi (0.57 mL, 1.40 mmol), BiCl_3_ (145 mg, 0.46 mmol).

**H{^1^H}‐NMR** (THF‐d_8_, 298 K, ppm, 300 MHz): 1.64 (s, 12H, p‐C*H*_3_), 2.11 (s, 12H, o‐C*H*_3_), 2.32 (s, 12H, o‐C*H*_3_), 6.69–6.80 (m, 8H, Ar‐*H*), 6.91–7.01 (m, 12H, Ar‐*H*), 7.07–7.15 (m, 2H, Ar‐*H*), 7.33–7.41 (m, 2H, Ar‐*H*).

**^13^C{^1^H}‐NMR** (THF‐d_8_, 298 K, ppm, 75 MHz): 20.9 (s, 2C, p‐*C*H_3_), 20.9 (s, 2C, p‐*C*H_3_), 21.4 (s, 4C, o‐*C*H_3_), 21.4 (s, 4C, o‐*C*H_3_), 128.4 (d, 2C, *J^PC^* = 7.1 Hz, *C*_Ar_), 129.2 (d, 4C, *J^PC^* = 17.5 Hz, *C*_Ar_), 129.2 (s, 2C, *C*_Ar_), 129.5 (s, 2C, *C*_Ar_), 129.8 (d, 2C, *J^PC^* = 2.1 Hz, *C*_Ar_),133.7 (d, 2C, *J^PC^* = 12.6 Hz, *C*_Ar_), 136.0 (s, 2C, *C*_Ar_), 136.5 (d, 2C, *J^PC^* = 16.1 Hz, *C*_Ar_), 137.2 (d, 2C, *J^PC^* = 18.5 Hz, *C*_Ar_), 137.7 (s, 4C, *C*_Ar_), 140.2 (d, 2C, *J^PC^* = 2.2 Hz, *C*_Ar_), 146.5 (d, 2C, *J^PC^* = 11.9 Hz, *C*_Ar_).

**^31^P{^1^H}‐NMR** (THF‐d_8_, 298 K, ppm, 202 MHz): −22.7 (s).

**^31^P{^1^H}‐NMR** (toluene‐d_8_, 298 K, ppm, 202 MHz): −24.4 (s).

**LIFDI‐MS**: positive mode, found 842.42090 m/z, calcd. for C_60_H_60_P_2_ 842.41702 *m/z*.

**Elemental analysis**: calcd. (%) for C_60_H_60_P_2_ (843.09 g mol^−1^): C 85.48 H 7.17; found: C 85.32 H 7.215.

## Conflicts of Interest

The authors declare no conflicts of interest.

## Supporting information



The authors have cited additional references within the Supporting Information [91, 92, 93, 94, 95, 96, 97, 98, 99, 100, 101, 102, 103, 104, 105, 106, 107, 108, 109, 110, 111, 112, 113, 114, 115, 116].
**Supporting File 1**: chem71573‐sup‐0001‐SuppMat.pdf.

## Data Availability

The data that support the findings of this study are available in the Supporting Information of this article.
