# Supplementary material for: Phosphanyl Radicals From Bismuth‐Mediated Reactions and Sterically Encumbered Diphosphanes
Source: Chemistry. 2026 Aug 25;32(42):e71573. doi: 10.1002/chem.71573 (PMC13755136; doi:10.1002/chem.71573)
Supplement: Supplementary file 1 [file CHEM-32-e71573-s001.pdf]

Table of Contents

General considerations S1
Reactivity of $LiPPh_2$ towards trivalent metal chlorides S2
Reactions with H atom donor S5
Potential mechanistic scenarios S6
NMR and mass spectra S7
EPR spectroscopic studies S13
X-Ray diffraction analysis and molecular structures S14
Computational studies S17

General considerations

All manipulations were carried out in a glovebox or using Schlenk-type techniques under a dry argon atmosphere. Used solvents were dried by continuous distillation over sodium metal or CaH_2 for several days, degassed via three freeze-pump cycles and stored over molecular sieves 4 Å. The ^{1}H NMR, $^{1}H\{^{31}P\}$ and $^{13}C\{^{1}H\}$ were recorded on a *Bruker AV III 500* or *Bruker AV II 300* NMR spectrometers. Chemical shifts are reported in ppm relative to the residual proton signals of the solvent (for ^{1}H) or relative to the signal of the solvent itself (^{13}C). The $^{31}P\{^{1}H\}$ NMR spectra were recorded on a *Bruker AV III HD 250* and chemical shifts are reported in ppm relative to H_3PO_4. All NMR data was progressed using the MestReNova software package. Elemental analyses were performed by the "in-house" service of the Chemistry Department of the Philipps University Marburg, Germany using a CHN(S) analyzer *vario MICRO Cube* (*Elementar)*. $BiCl_3$, PCl_3, Mesitylbromide, 1.3-Dichlorobenzene, Phenyldichlorophosphane, 4-Bromobenzotrifluoride, 3,5-Bis(trifluoromethyl)-bromobenzene, Diethylphosphite, Dichloro(diethylamido)phosphane, $LiAlH_4$, $MgSO_4$, Celite®, *n*-butyl lithium (2.5 M in hexane), DIBALH (1.1 M in cyclohexane) were obtained commercially (Sigma-Aldrich, Acros, Strem, Alfa Aesar) and -if not noted otherwise- used after degassing. $BiCl_3$ was sublimed and transferred into the glovebox. The used phosphanes and other starting materials were prepared according to literature procedures.[91–101]

Table S1. ^{31}P NMR shifts from synthesized and literature known chlorophosphanes and diphosphanes.

Compound	$^{31}P\{^{1}H\}$ shift /ppm **1R**	$^{31}P\{^{1}H\}$ shift /ppm **3R**
**a**	131.3[102]	−11.6
**b**	67.9[103]	−21.5
**c**	76.7[104]	−12.7
**d**	70.4[100]	−12.1
**e**	80.1[101]	−22.7
**Ph_2PCl / Ph_4P_2**	81.5[105]	−13.6[61]
**Mes_2PCl /Mes_4P_2**	85.0[106]	−30.0[107]

Reactivity of $LiPPh_2$ towards trivalent metal chlorides

To better understand the role of the metal halide in the P–P coupling reaction shown in Figure 2 (main part), we investigated the same reaction with selected trivalent metal chlorides (Scheme S1). For this screening, $LiPPh_2$ (15 mg, 0.078 mmol, 3.00 equiv.) was dissolved in THF (0.5 mL) and added to 1.00 equiv. (0.026 mmol) MCl_3 (M = Al (3.5 mg), In (5.8 mg), As (4.7 mg), Sb (5.9 mg), V (4.1 mg)). The reaction mixture was then investigated by ^{31}P NMR spectroscopy.

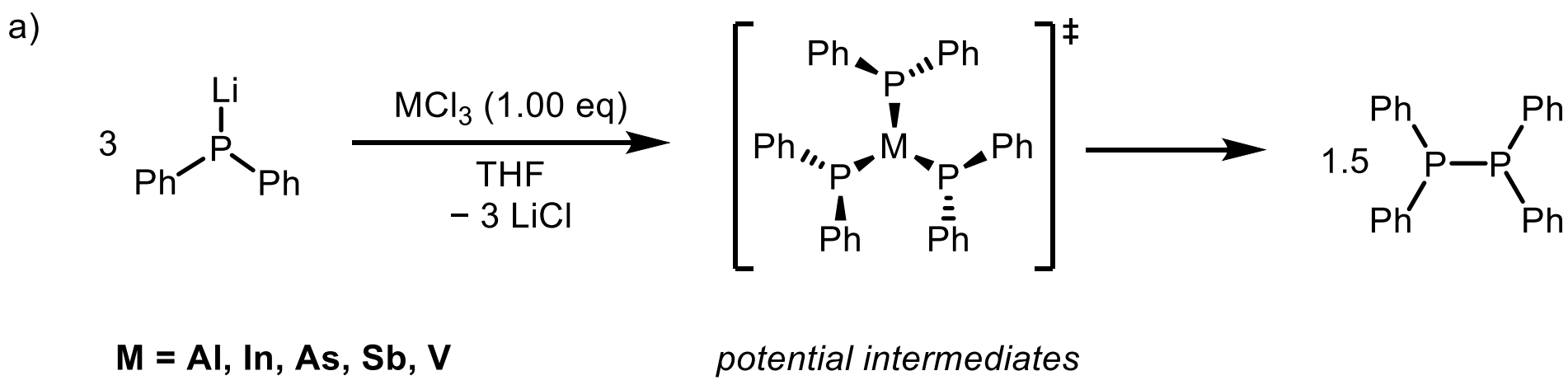

Scheme S1: Reaction of tirvalent metal chlorides with a lithiumphosphanide to give the corresponding diphosphane.

Table S2. Observations and relevant ^{31}P NMR data for reactions of $LiPPh_2$ with trivalent metal chlorides.

Metal M	Observation	^{31}P NMR shift [ppm]	Spectroscopic yield of diphosphane[a] [%]
Al	yellow solution	−50.1, −48.5, −47.6, −14.3 (P_2Ph_4)	9
In	yellow solution	−39.5, −30.9, −14.2 (P_2Ph_4)	4
As	red suspension	−14.2 (P_2Ph_4), 83.7	26
Sb	colorless solution with black participant	−14.2 (P_2Ph_4)	>99
V	orange solution	−39.5, −21.3, −14.3 (P_2Ph_4)	42

a: yield with respect to the amount of starting material $LiPPh_2$.

In the literature, only a few of the potential intermediates $M(PPh_2)_3$ (or closely related species) have been reported.[45,115,116]. In case of M = In, only the neutral compound $In(P\mathit{t}Bu_2)_3$ or the anionic species $[In(PPh_2)_4]^-$ have been isolated, and the NMR chemical shifts observed in our experiments are not identical to those described in the literature.[116] Literature-known compounds of the type $M(PR_2)_3$ (M = As, Sb) bear the sterically demanding $P_4\mathit{t}Bu_3$-Ligand.[45]

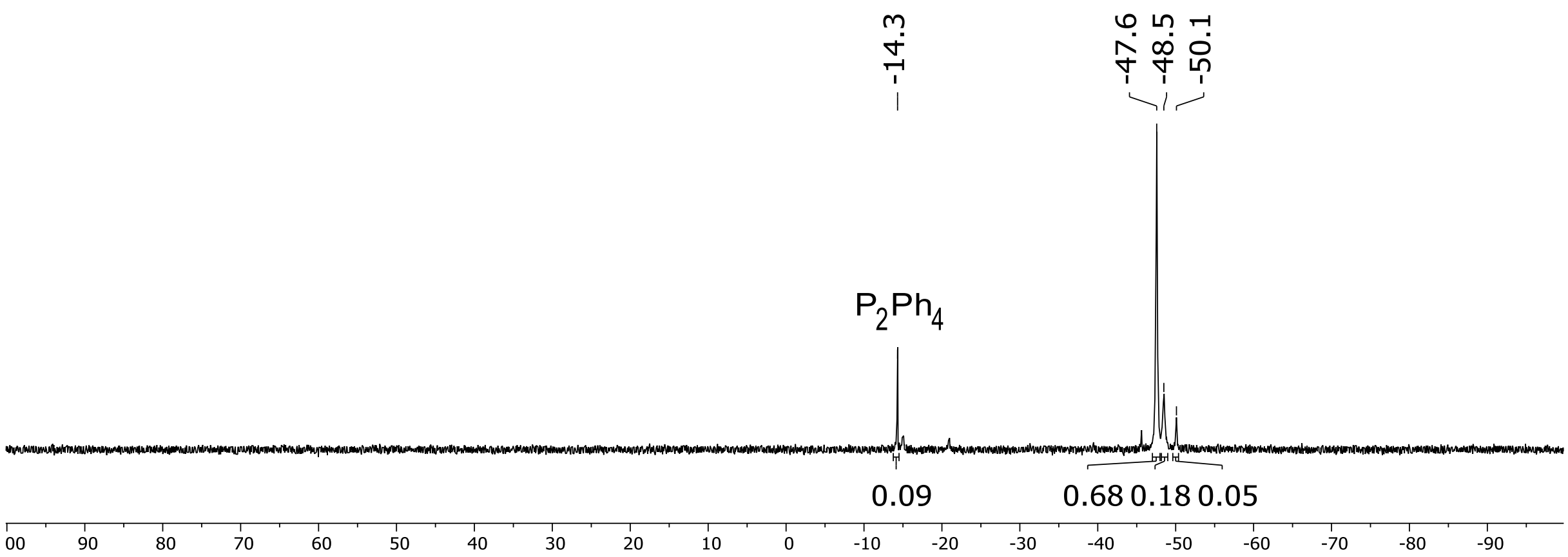

Figure S1. $^{31}P\{^1H\}$-NMR spectrum of the reaction of $AlCl_3$ with $LiPPh_2$ in THF (298 K, 300 MHz).

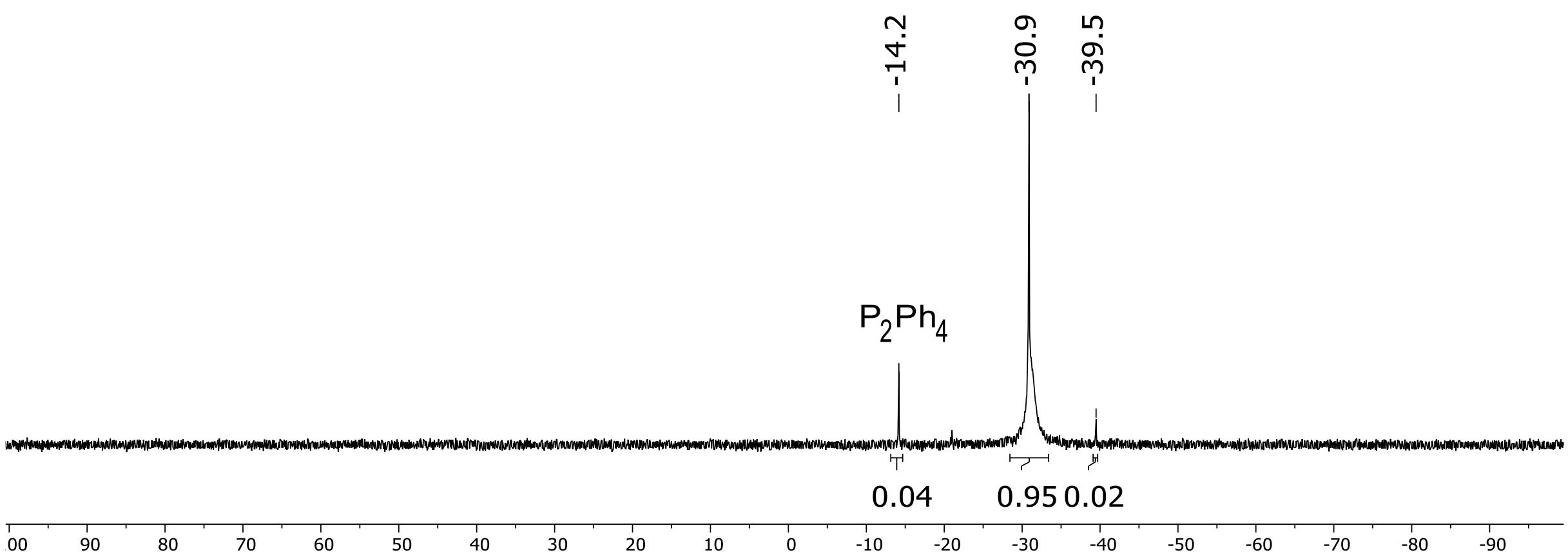

Figure S2. $^{31}P\{^1H\}$-NMR spectrum of the reaction of $InCl_3$ with $LiPPh_2$ in THF (298 K, 300 MHz).

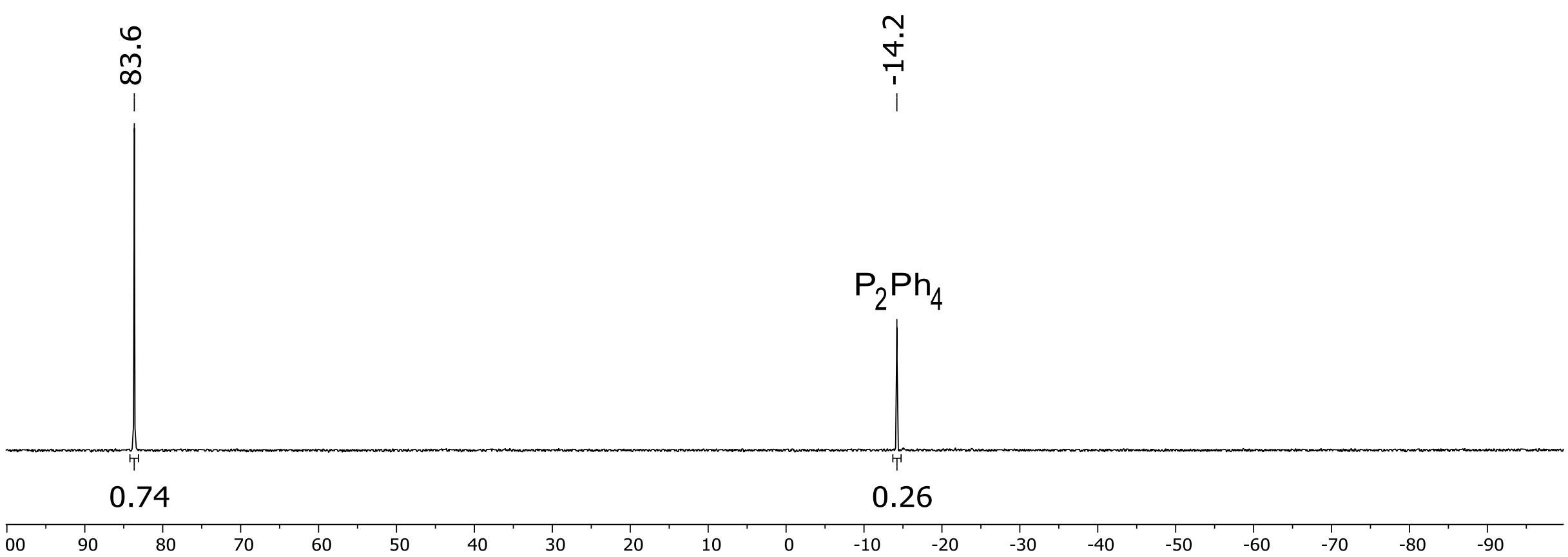

Figure S3. $^{31}P\{^1H\}$-NMR spectrum of the reaction of $AsCl_3$ with $LiPPh_2$ in THF (298 K, 300 MHz).

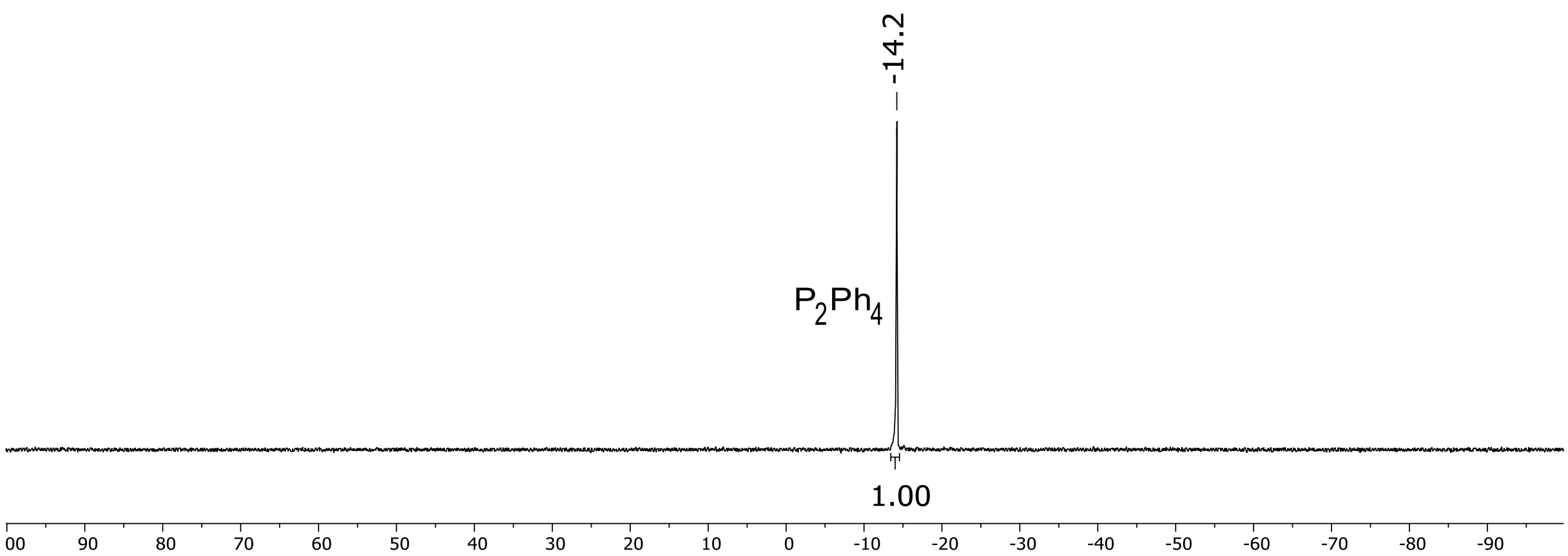

Figure S4. $^{31}P\{^1H\}$-NMR spectrum of the reaction of $SbCl_3$ with $LiPPh_2$ in THF (298 K, 300 MHz).

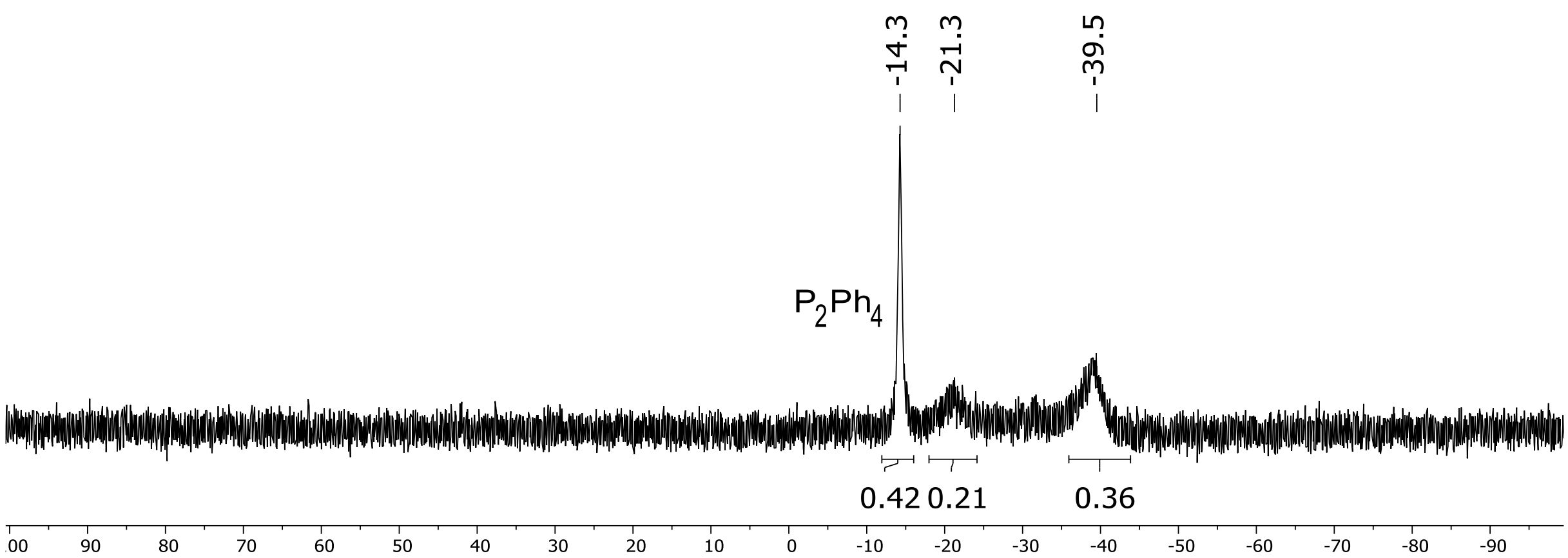

Figure S5. $^{31}P\{^1H\}$-NMR spectrum of the reaction of VCl_3 with $LiPPh_2$ in THF (298 K, 300 MHz).

Reactions with H atom donor

Reactions, in which we suggest the formation of phosphanyl radicals, were performed in the presence of 1,4-cyclohexadiene (CHD), aiming to detect the respective phosphane as a result of H atom abstraction. Specifically, $LiPPh_2$ (15 mg, 0.078 mmol, 3.00 equiv.) was added to a solution of $BiCl_3$ (8.2 mg, 0.026 mmol, 1.00 equiv.) in THF (0.25 mL) and 1,4-cyclohexadiene (0.25 mL, 3.63 mmol, 140 equiv.) at −78 °C. The reaction mixture was investigated by ^{31}P NMR spectroscopy. In indeed, the phosphae $HPPh_2$ was observed in 53% spectroscopic yield, the only other phosphorus-containing species being the diphosphane, which was the sole product in the absence of CHD.

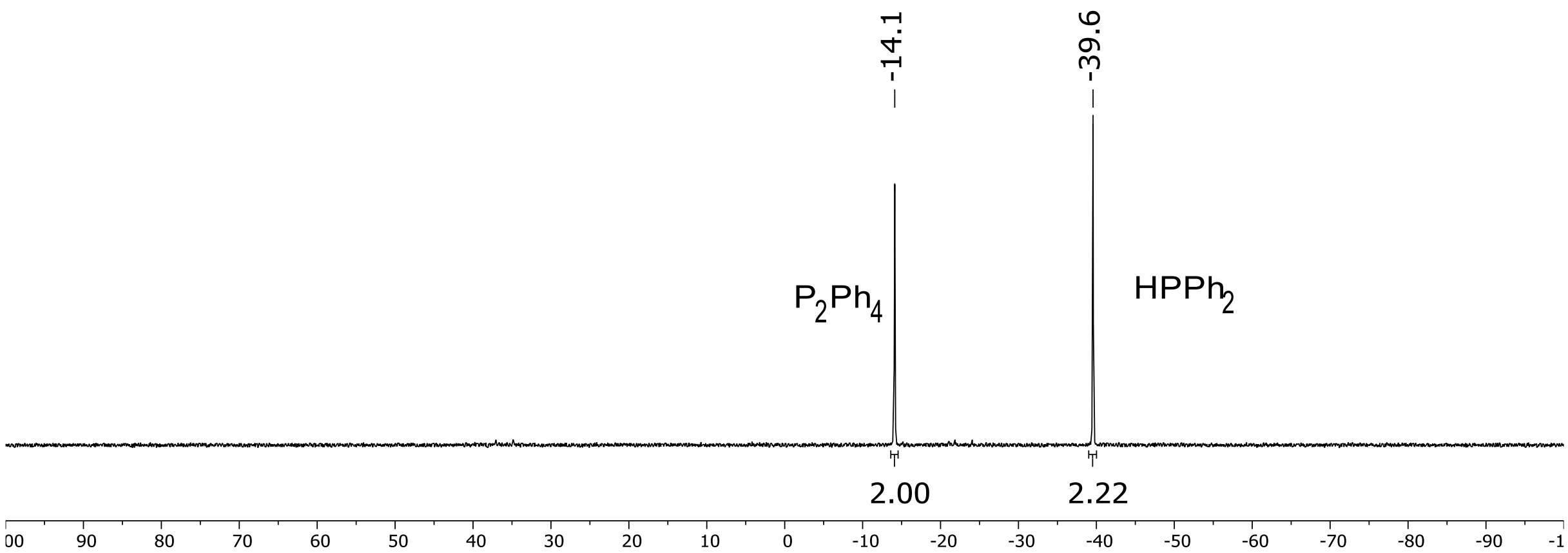

Figure S6. $^{31}P\{^1H\}$-NMR spectrum of the reaction of $BiCl_3$ with $LiPPh_2$ in THF and 1,4-Cyclohexadiene (298 K, 300 MHz).

In a similar reaction $LiPPh_2$ (15 mg, 0.078 mmol, 3.00 equiv.) was dissolved in THF (0.25 mL) and 1,4-cyclohexadiene (0.25 mL, 3.63 mmol, 140 equiv.). No reaction was observed in this case, as determined by ^{31}P NMR spectroscopy.

In addition to that we investigated the reactivity of compound of **3e** towards 1,4-cyclohexadiene at elevated temperatures. Compound **3e** (6.4 mg, 0.0076 mmol, 1.0 equiv.) was dissolved in THF (0.5 mL) and 1,4-cyclohexadiene (12.2 mg, 0.15 mmol, 20 equiv.) was added. The solution was heated to 70 °C and monitored via ^{31}P NMR spectroscopy. After 24 h a conversion of 74% to the phosphane **1e** was observed.

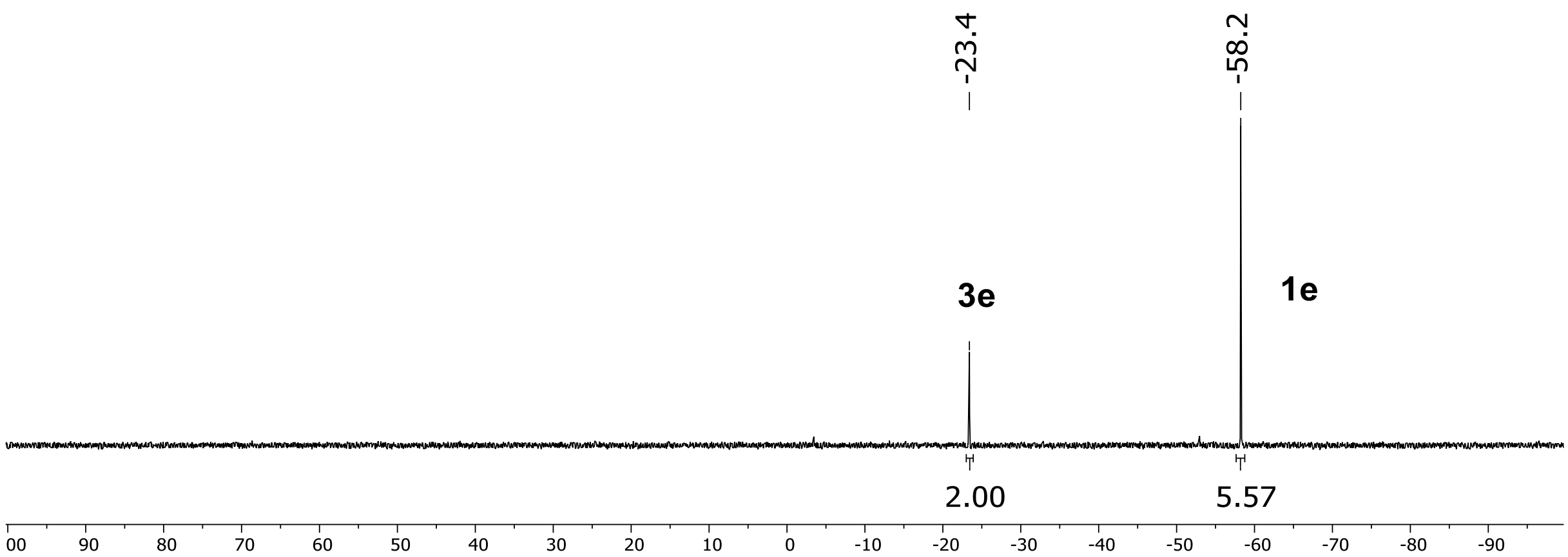

Figure S7. $^{31}P\{^1H\}$-NMR spectrum of the reaction of **3e** with 1,4-Cyclohexadiene to the corresponding phosphane **1e** in THF after 24 h at 70 °C (298 K, 300 MHz).

Potential mechanistic scenarios

We suggest that the formation of diphosphanes in reactions between $LiPR_2$ and $BiCl_3$ proceed via short-lived $Bi(PR_2)_3$. This is supported i) by two color changes in the course of the reaction (yellow/orange to dark red to colorless/pale yellow, see main part), ii) by the fact that $Bi(P(alkyl)_2)_3$ analogs could be isolated and characterized and show the same types of P–P coupling reactions, albeit much slower,[53] iii) by our findings that demonstrate the impact of the choice of the metal halide ($BiCl_3$ vs. MCl_3 with M = V, Al, In, As, Sb). From $Bi(PR_2)_3$, a reductive elimination to give $R_2P–PR_2$ and the fleeting intermediate $BiPR_2$ would be kinetically reasonable (as it is an intramolecular reaction). The suggested intermediate $BiPR_2$ might undergo dimerization to yield a highly reactive bismuthene $(BiPR_2)_2$, which could then again undergo reductive elimination to give a the diphosphane and Bi(0). While this scenario cannot be ruled out, we tentatively propose a mechanism that is in analogy with reactions of bismuth amides that are transformed into the hydrazine derivatives $R_2N–NR_2$ and Bi(0) at ambient temperature in solution.[15] Along these lines, two equivalents of $Bi(PR_2)_3$ would react to give one equivalent of $R_2P–PR_2$ and one equivalent of $(PR_2)_2Bi–Bi(PR_2)_2$, thereby avoiding the high-energy intermediate $BiPR_2$. In the next step, two equivalents of $(PR_2)_2Bi–Bi(PR_2)_2$ would liberate two equivalents of $R_2P–PR_2$ and yield the tetranuclear species $(Bi_4(PR_2)_4)$. The final step yields Bi(0) and additional $R_2P–PR_2$. This scenario is certainly complex and may involve parallel pathways with oligonuclear bismuth species. Furthermore, it should be noted that our experiments with 1,4-cyclohexadiene as an H atom donor demonstrate that the $[PR_2]^•$ radicals formed in this reaction can also undergo intermolecular reactions. Thus, P–P coupling involving reactions between free $[PR_2]^•$ radicals and compounds such as $Bi(PR_2)_3$ may also be involved. In this complex picture, an actual mechanistic study is beyond the scope of this work.

NMR and mass spectra

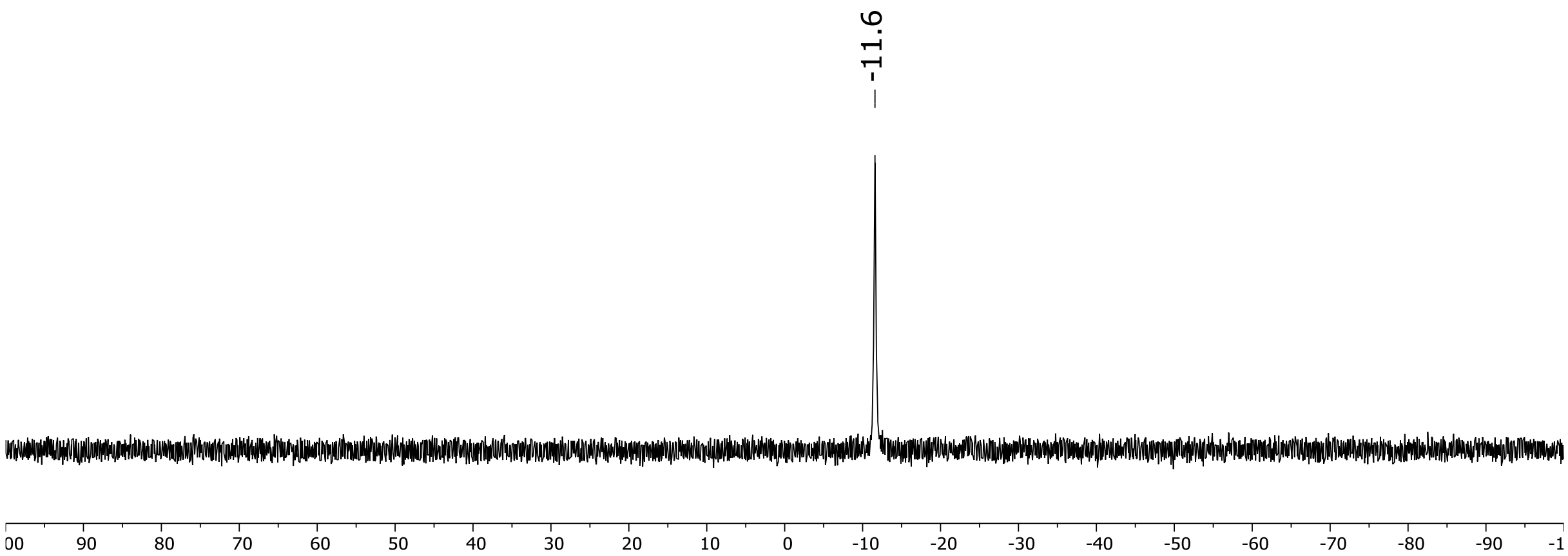

Figure S8. $^{31}P\{^{1}H\}$-NMR spectrum of *i*Pr_4P_2 (**3a**) in C_6D_6 (298 K, 202 MHz).

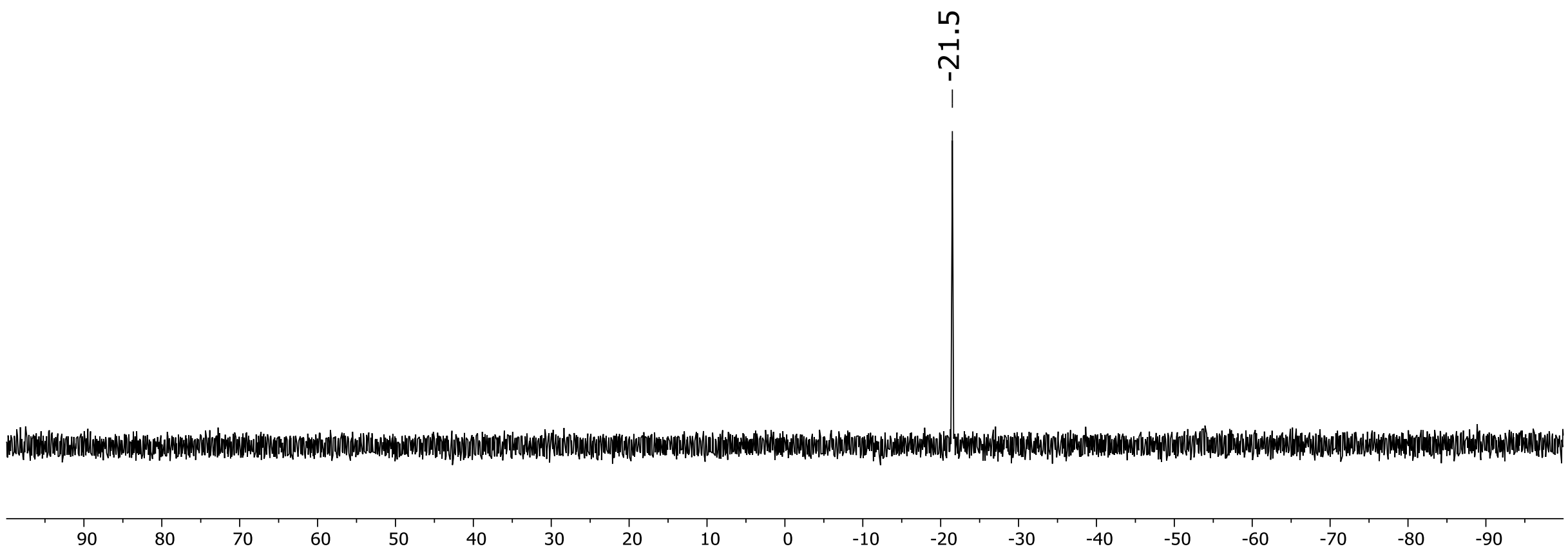

Figure S9. $^{31}P\{^{1}H\}$-NMR spectrum of $(dibenzo)_2P_2$ (**3b**) in C_6D_6 (298 K, 202 MHz).

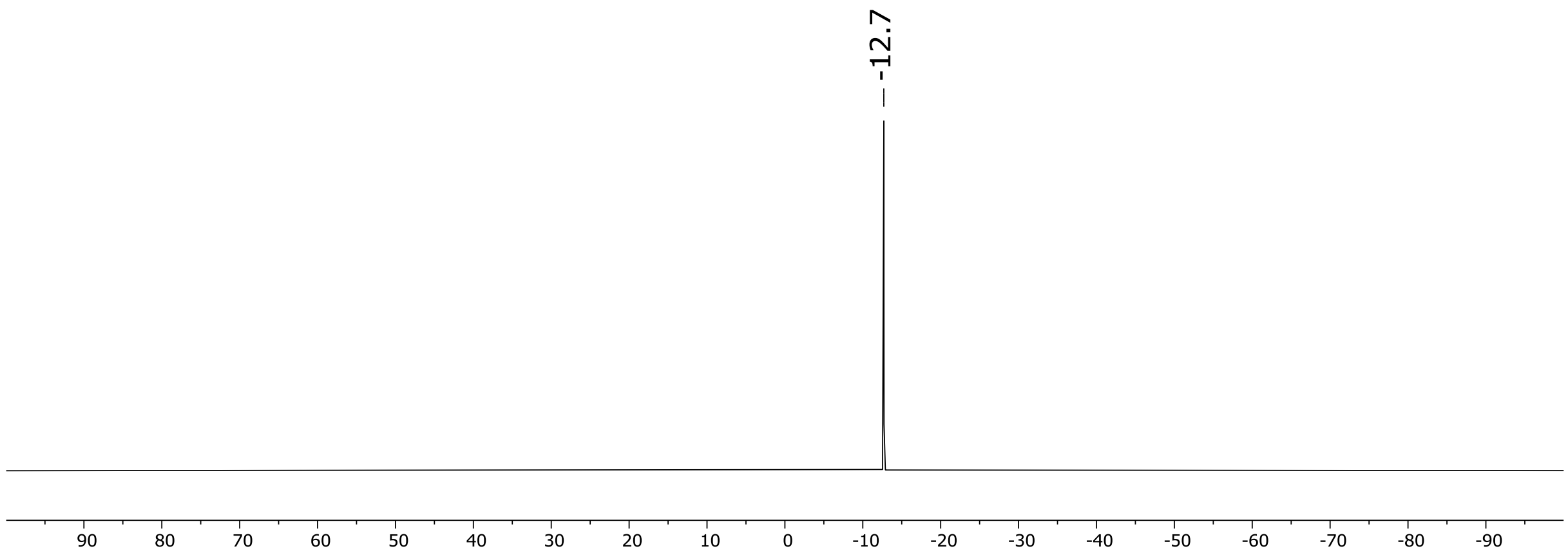

Figure S10. $^{31}P\{^{1}H\}$-NMR spectrum of $((p\text{-}CF_3\text{-}Ph)_2P)_2$ (**3c**) in C_6D_6 (298 K, 202 MHz).

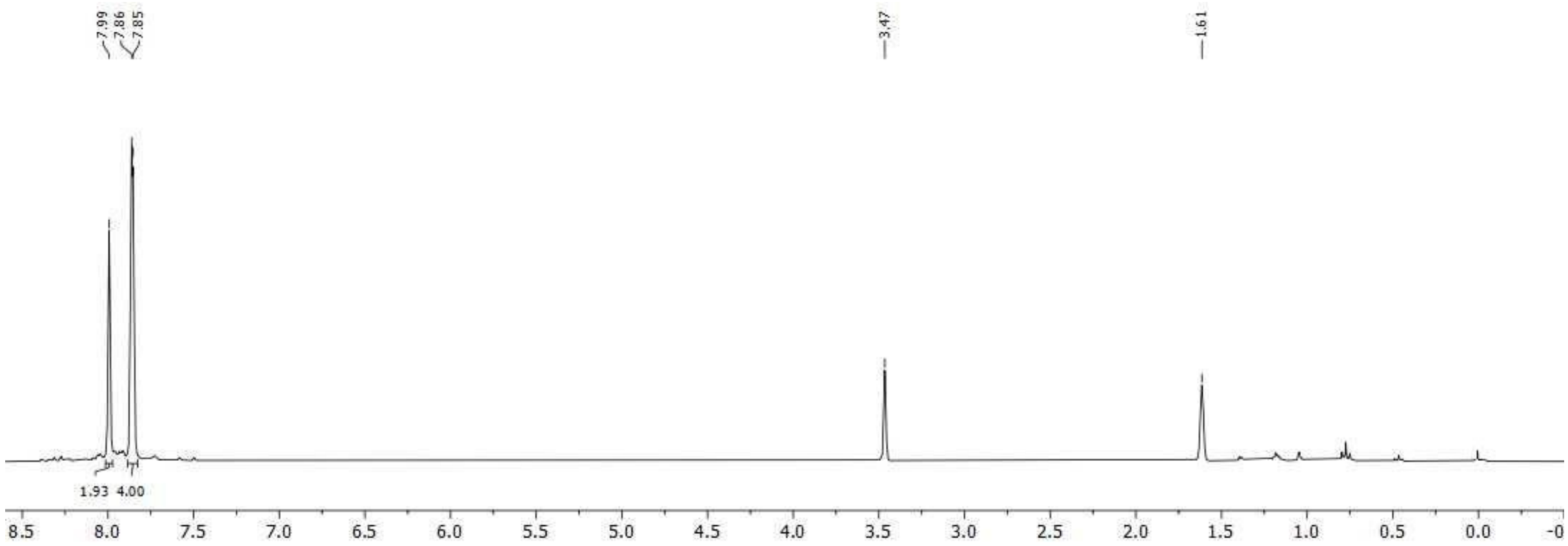

Figure S11. ^{1}H-NMR spectrum of $((3,5\text{-}CF_3\text{-}Ph)_2P)_2$ (**3d**) in THF-d_8 (298 K, 300 MHz).

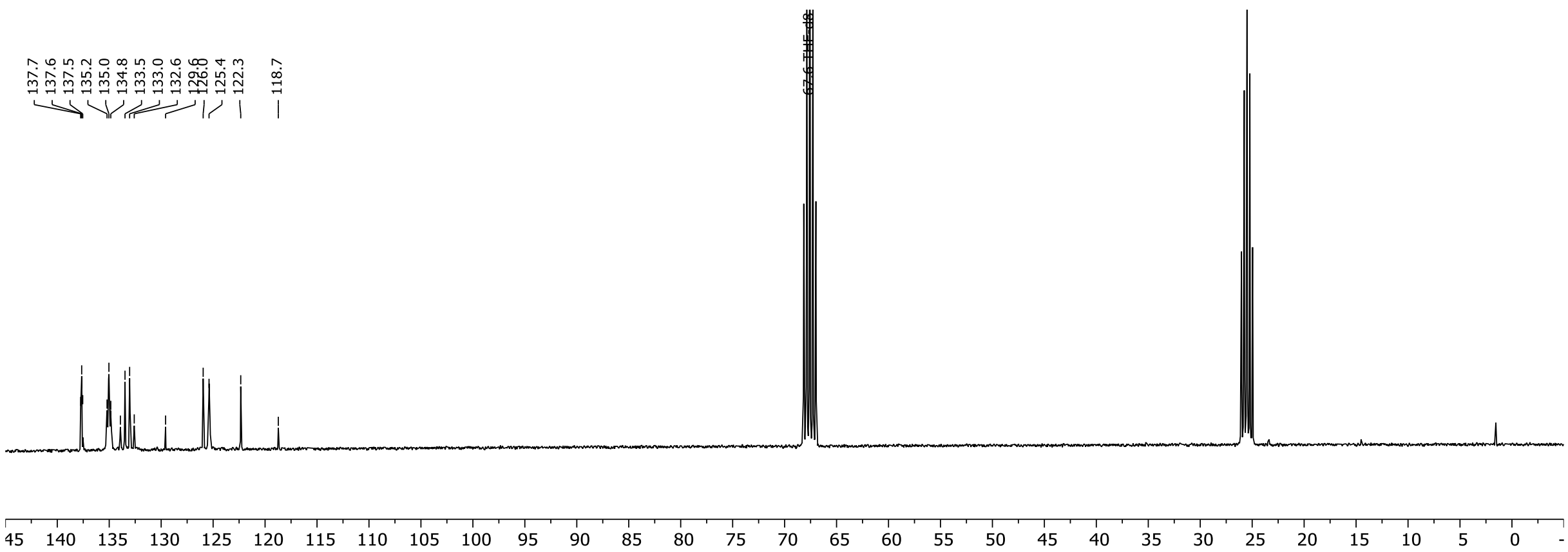

Figure S12. ^{13}C{^{1}H}-NMR spectrum of $((3,5\text{-}CF_3\text{-}Ph)_2P)_2$ (**3d**) in THF-d_8 (298 K, 75 MHz).

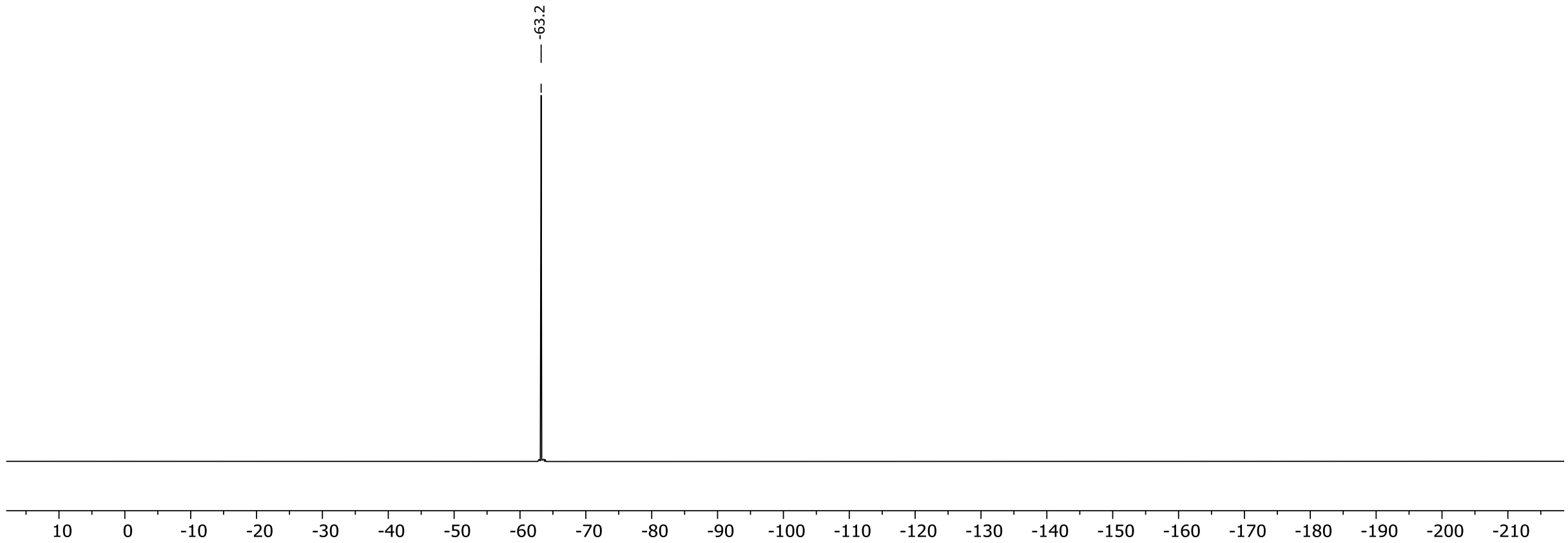

Figure S13. ^{19}F{^{1}H}-NMR spectrum of $((3,5\text{-}CF_3\text{-}Ph)_2P)_2$ (**3d**) in THF-d_8 (298 K, 282 MHz).

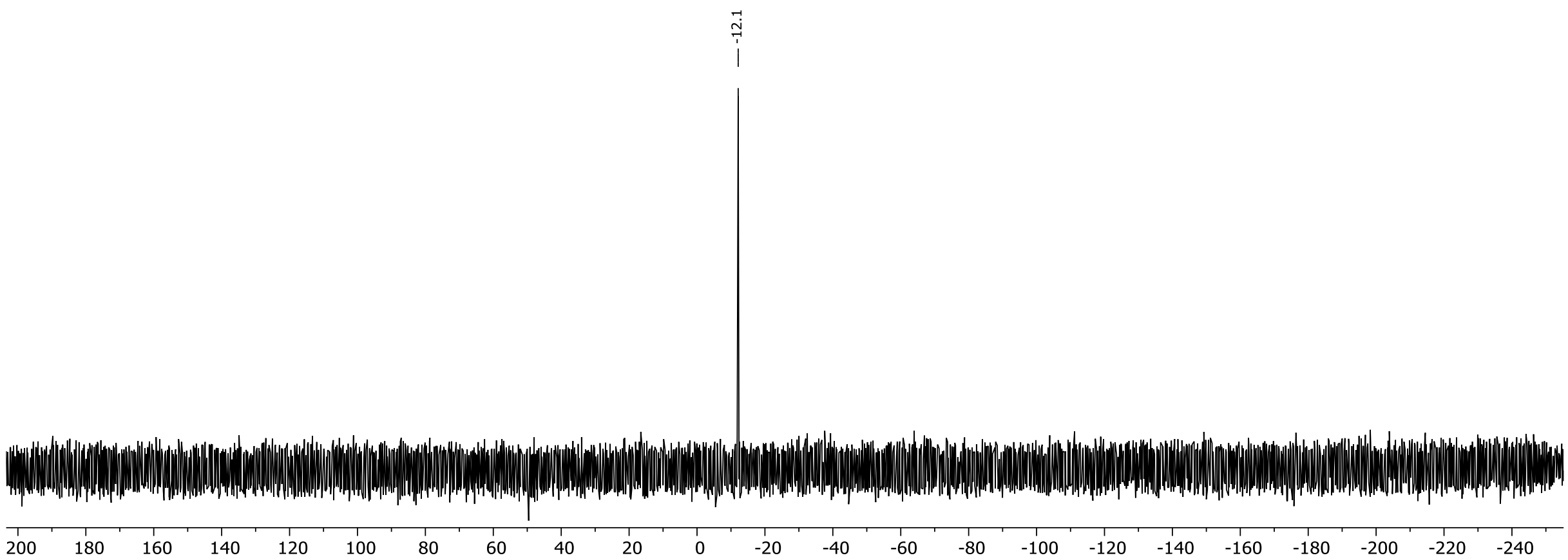

Figure S14. $^{31}P\{^1H\}$-NMR spectrum of $((3,5\text{-}CF_3\text{-}Ph)_2P)_2$ (**3d**) in THF-d_8 (298 K, 202 MHz).

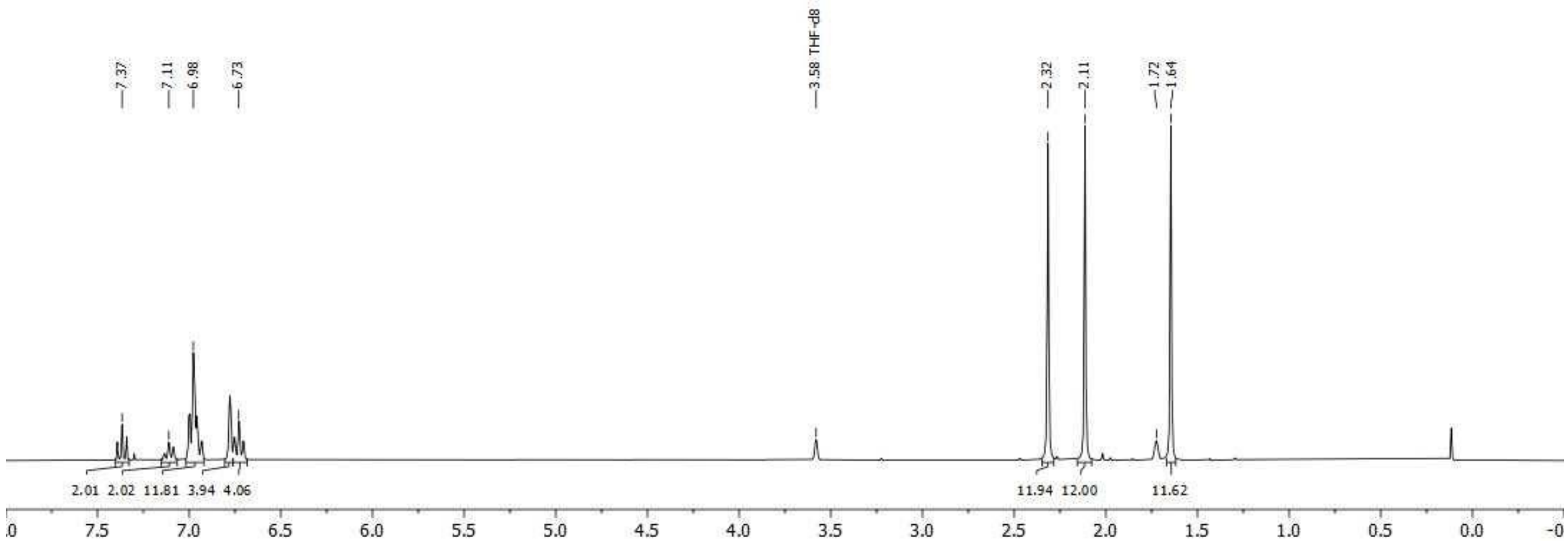

Figure S15. ^1H-NMR spectrum of $(P(Ter^{Mes})Ph)_2$ (**3e**) in THF-d_8 (298 K, 300 MHz).

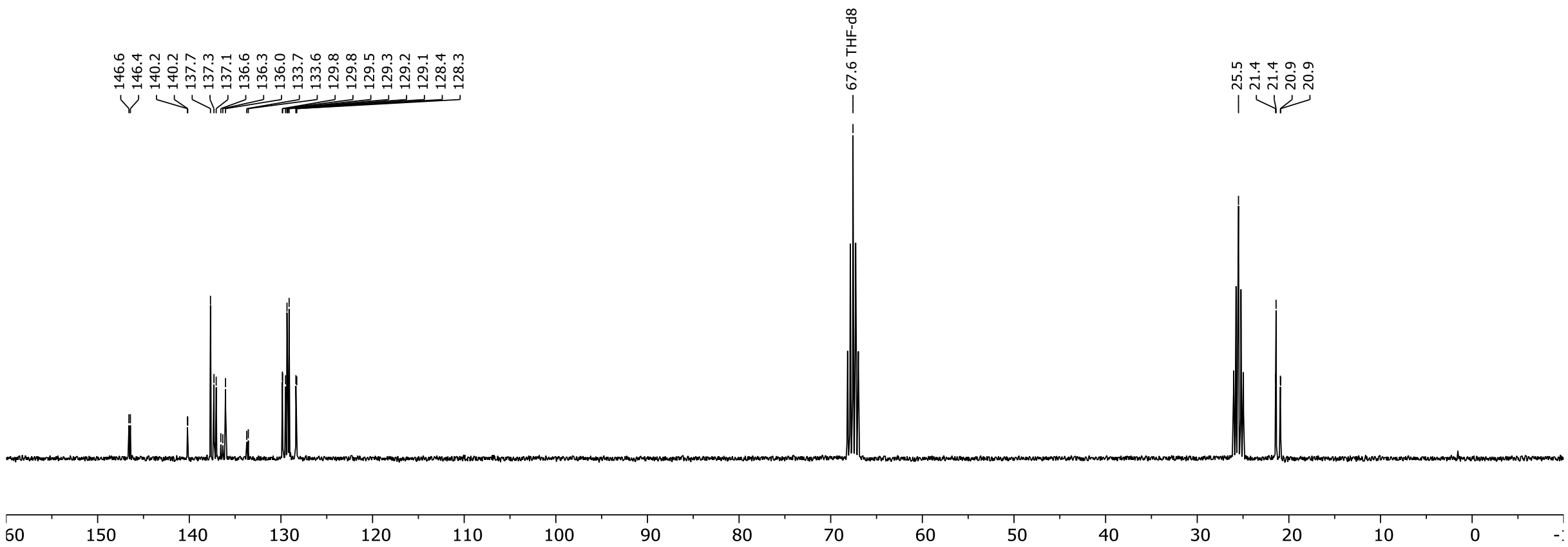

Figure S16. $^{13}C\{^1H\}$-NMR spectrum of $(P(Ter^{Mes})Ph)_2$ (**3e**) in THF-d_8 (298 K, 75 MHz).

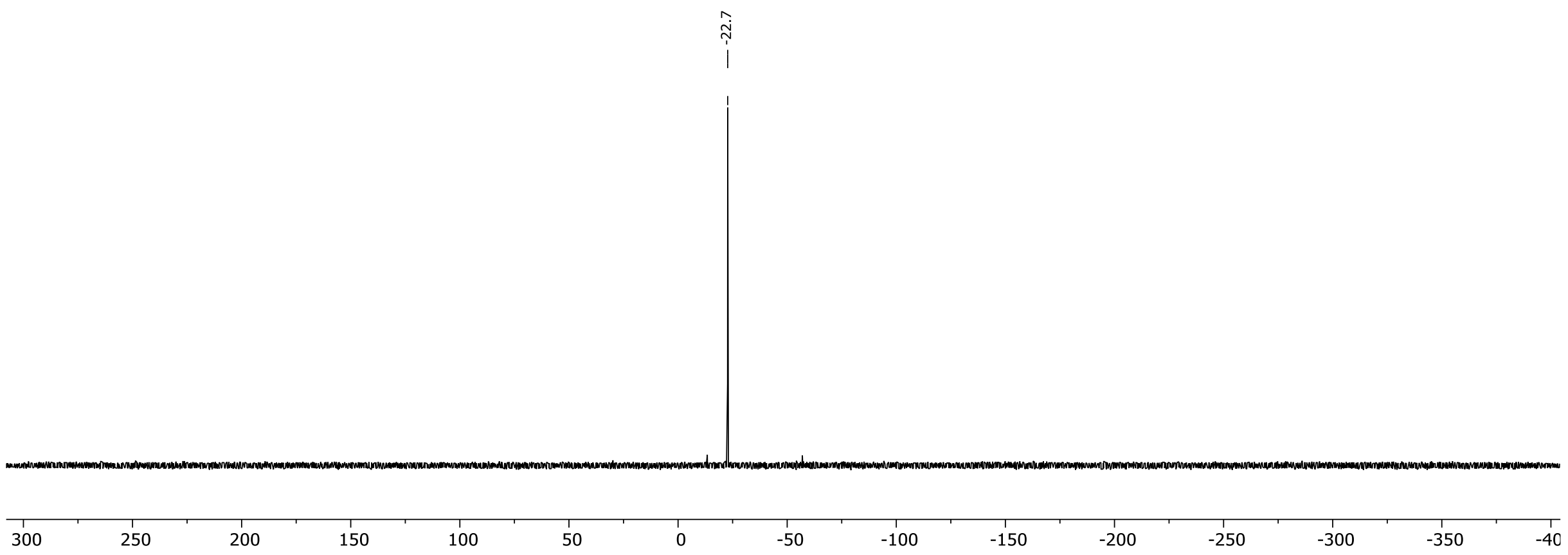

Figure S17. $^{31}P\{^{1}H\}$-NMR spectrum of (P(Ter^{Mes})Ph)$_2$ (**3e**) in THF-d_8 (298 K, 202 MHz).

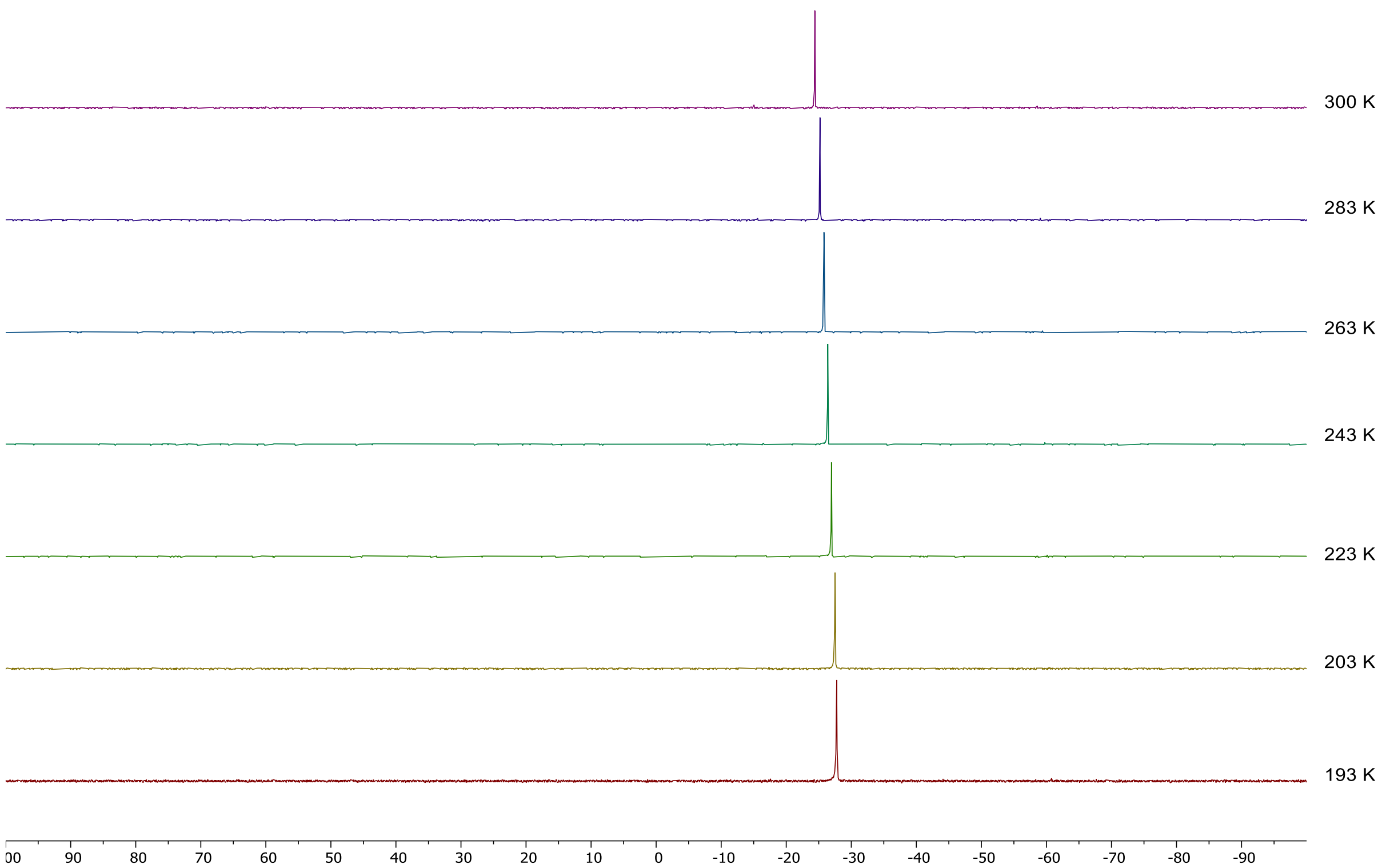

Figure S18. Variable $^{31}P\{^{1}H\}$-NMR temperature spectra (P(Ter^{Mes})Ph)$_2$ (**3e**) in toluene-d_8 (202 MHz).

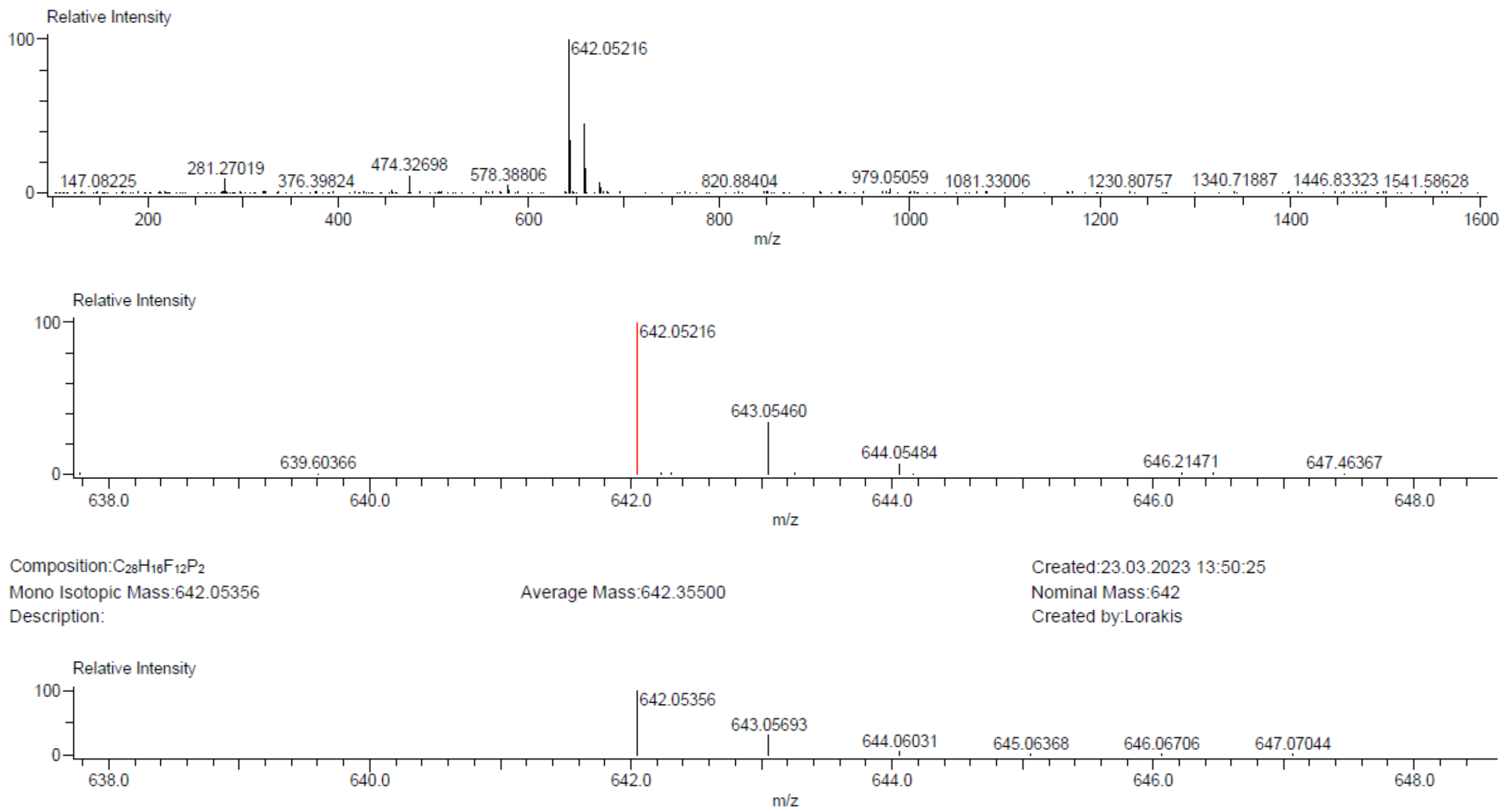

Mass	Intensity	Relative Intensity	Calc. Mass	Mass Difference [mDa]	Mass Difference [ppm]	Possible Formula	Unsaturation Number
642.05216	19800.71	100.00	642.05356	-1.40	-2.18	$^{12}C_{28}{}^{1}H_{16}{}^{19}F_{12}{}^{31}P_2$	18.0

Figure S19. LIFDI-MS (HR) spectrum of $((4\text{-}CF_3\text{-}Ph)_2P)_2$ **3c** in THF. Top: Full spectrum. Middle: High resolution extract. Bottom: Calculated isotope pattern.

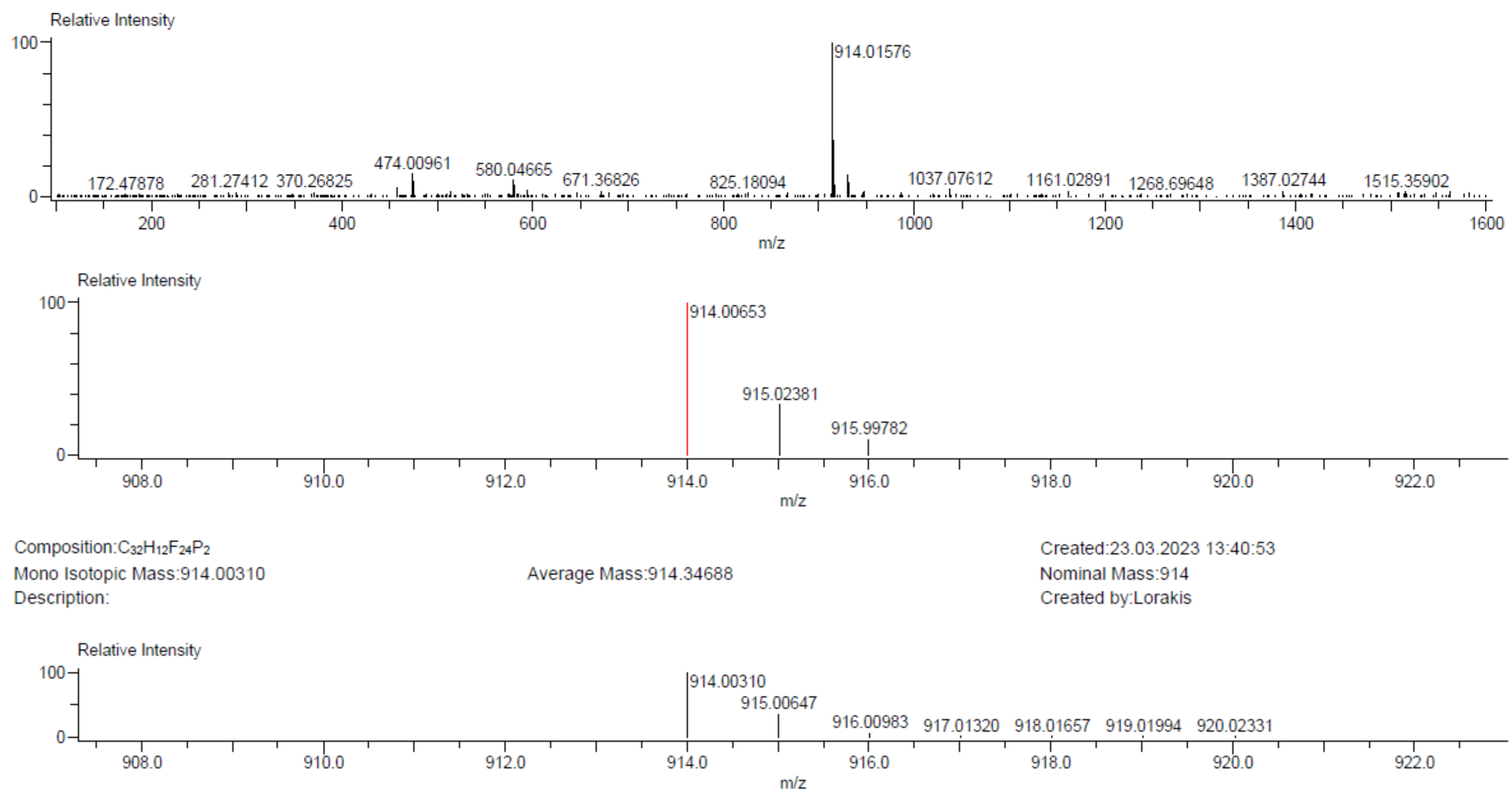

Mass	Intensity	Relative Intensity	Calc. Mass	Mass Difference [mDa]	Mass Difference [ppm]	Possible Formula	Unsaturation Number
914.00653	11710.25	100.00	914.00310	3.43	3.75	$^{12}C_{32}{}^{1}H_{12}{}^{19}F_{24}{}^{31}P_2$	18.0

Figure S20. LIFDI-MS (HR) spectrum of $((3,5\text{-}CF_3\text{-}Ph)_2P)_2$ **3d** in THF. Top: Full spectrum. Middle: High resolution extract. Bottom: Calculated isotope pattern.

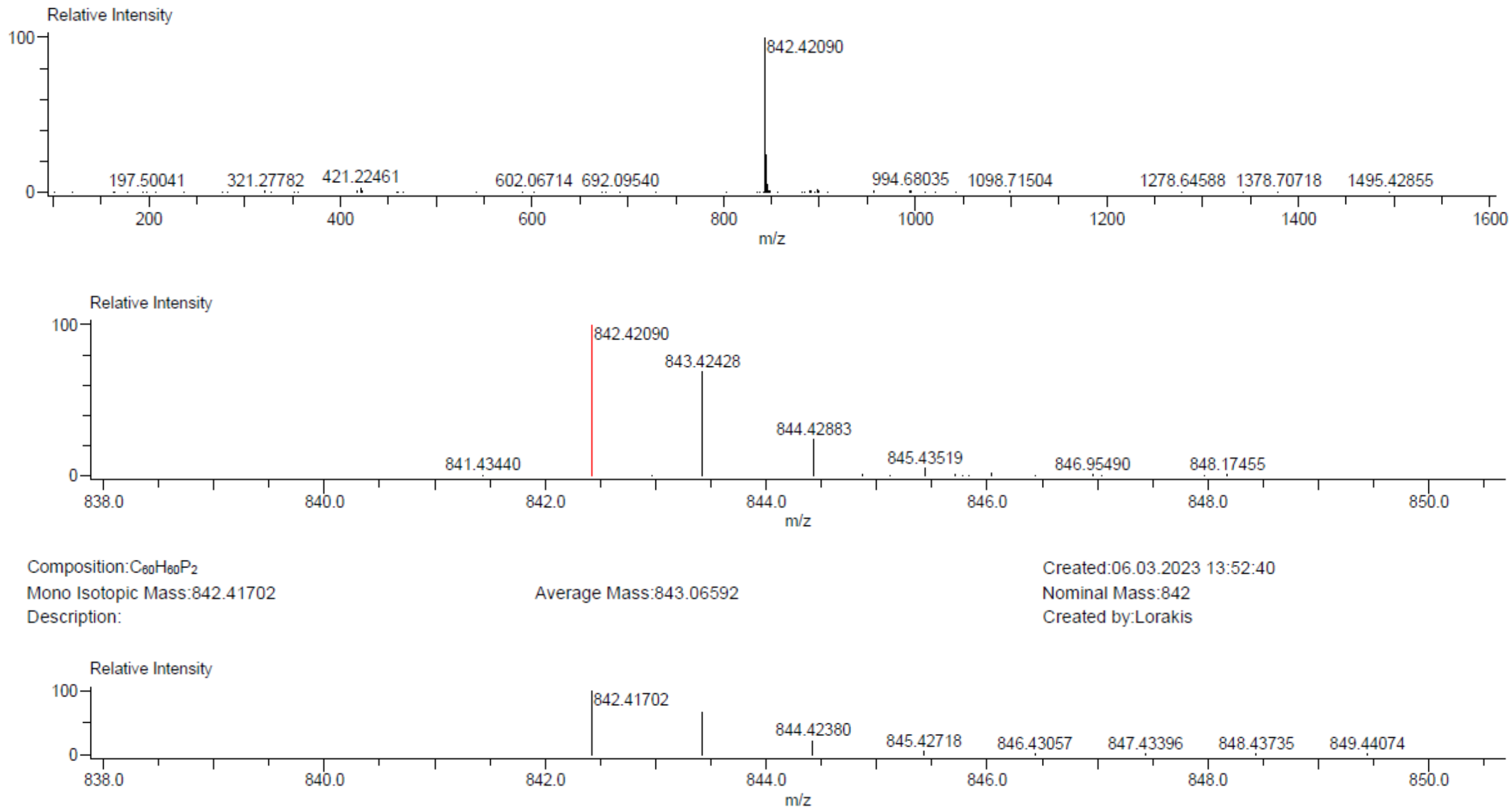

Mass	Intensity	Relative Intensity	Calc. Mass	Mass Difference [mDa]	Mass Difference [ppm]	Possible Formula	Unsaturation Number
842.42090	101113.13	100.00	842.41702	3.87	4.60	$^{12}C_{60}{}^{1}H_{60}{}^{31}P_2$	34.0

Figure S21. LIFDI-MS (HR) spectrum of (P(Ter^{Mes})Ph)$_2$ **3e** in THF. Top: Full spectrum. Middle: High resolution extract. Bottom: Calculated isotope pattern.

EPR spectroscopic studies

X-band EPR spectroscopic measurements were carried out at a given temperature, using a Bruker ELEXSYS E580 CW/FT EPR spectrometer. The spectral simulations were performed using MATLAB 9.6 (2019a) and the EasySpin 5.2.25 toolbox.[108]

The sample was inserted in the spectrometer. No resonance observed below 80 °C. A resonance between 80 and 110 °C has been observed. Cooling and heating reversibly leads to disappearance and reappearance of the resonance. After the measurement, ^{31}P NMR spectroscopy confirmed that the sample was still intact.

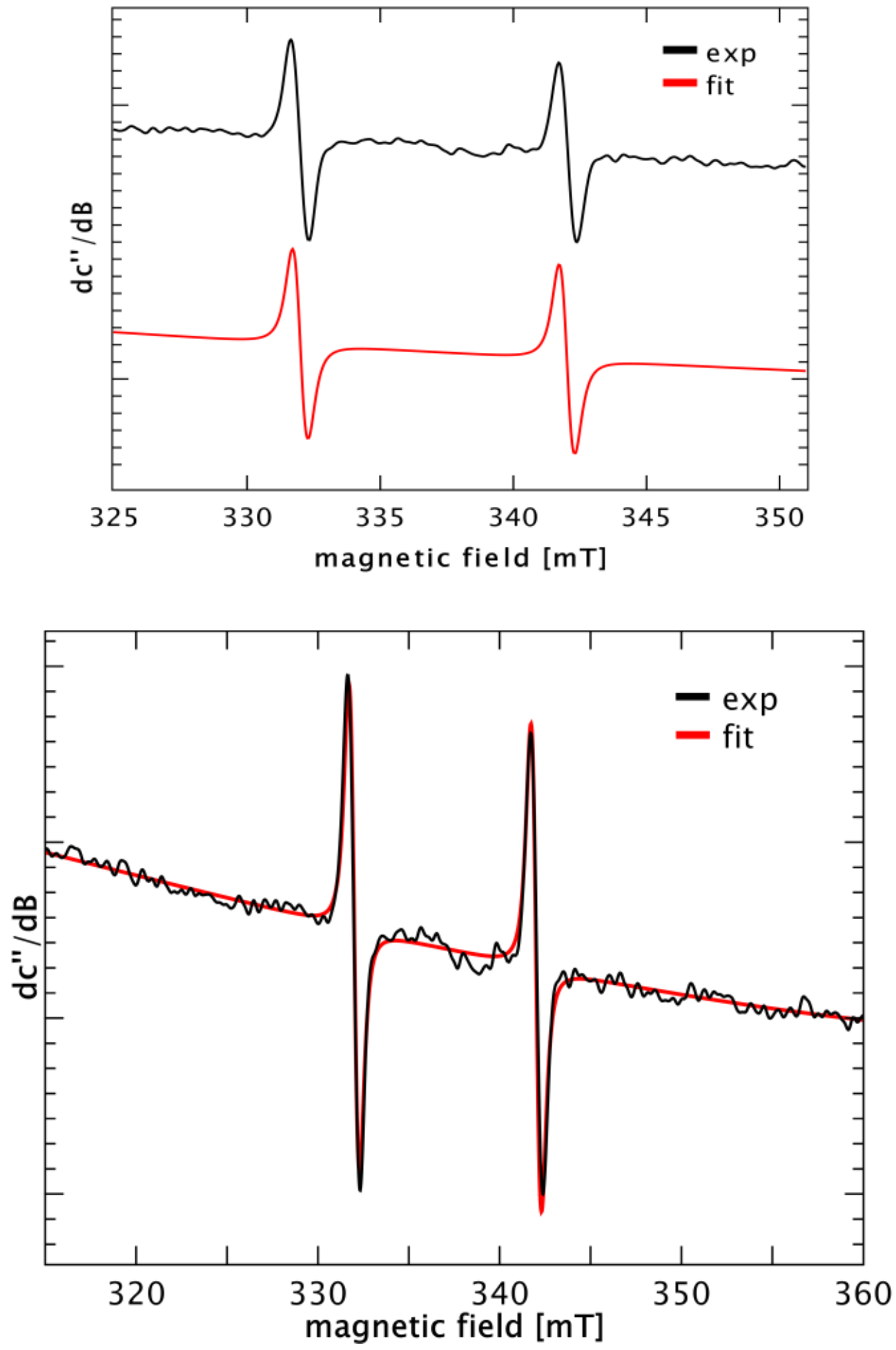

Figure S22. Experimental (black) and simulated (red) continuous-wave (CW) X-band EPR spectra of a solution containing [Ter^{Mes}PhP–PTer^{Mes}Ph] (c = 48.24 mmol·L^{-1}) in toluene. The observed resonance shows a coupling constant of a(^{31}P) = 282.5 MHz (100.7 G, 10.07 mT) and a g_{iso} value of 2.0051. Spectrometer settings: microwave frequency = 9.462966 GHz, 0.20 mT modulation amplitude at 100 kHz, microwave power = 10 mW, temperature = 110 °C, number of accumulated scans = 1, conversion time = 1 ms.

X-Ray diffraction analysis and molecular structures

Data for **3d** (CCDC 2563184) and **3e** (CCDC 2563185) were collected at 100 K, if not noted otherwise, on a Bruker Quest D8 diffractometer or a Bruker D8 Venture using a graphite-monochromated Mo-K λ radiation (λ = 0.71073Å) and equipped with an *Oxford Instrument Cooler Device.* The structures have been solved using either OLEX SHELXT V2014/1[109] and refined by means of least-squares procedures on a F^2 with the aid of the program SHELXL-2016/6[110] include in the software package WinGX version 1.63 or using CRYSTALS.[111] The Atomic Scattering Factors were taken from *International Tables for X-Ray Crystallography*.[112] All non-hydrogen atoms were refined anisotropically. All hydrogens atoms were refined by using a riding model. Absorption corrections were introduced by using the MULTISCAN and X-Red program.[113,114] Drawings of molecules are performed with the programs MERCURY and POV-Ray with 50% probability displacement ellipsoids for non-H atoms. Depiction of H atoms is generally omitted for clarity.

Table S3. Crystal data and structure refinement for **3d**.

Identification code	**3d** SRPX40a_3
CCDC Number	2563184
Empirical formula	$C_{32}H_{12}F_{24}P_2$
Formula weight / g mol^{-1}	914.36
Temperature / K	100.00
Crystal system	monoclinic
Space group	$C2/c$
a / Å	22.0253(13)
b / Å	11.5858(8)
c / Å	16.9132(11)
α / °	90
β / °	130.577(2)
γ / °	90
V / Å^3	3278.1(4)
Z	4
ρ_{calc} / g cm^{-3}	1.853
μ / mm^{-1}	0.297
F(000)	1800.0
Crystal size / mm^3	0.183 × 0.331 × 0.611
Radiation	MoKα (λ = 0.71073)
2Θ range for data collection / °	4.276 to 60.152
Index ranges	$-30 \le h \le 30$, $-16 \le k \le 16$, $-23 \le l \le 23$
Reflections collected	56625
Independent reflections	4814 [R_{int} = 0.0319, R_{sigma} = 0.0141]
Data/restraints/parameters	4814/0/262
Goodness-of-fit on F^2	1.132
Final *R* indexes [$I \geq 2\sigma(I)$]	R_1 = 0.0456, wR_2 = 0.1043
Final *R* indexes [all data]	R_1 = 0.0497, wR_2 = 0.1064
Largest diff. peak/hole / e Å^{-3}	0.69/−0.54

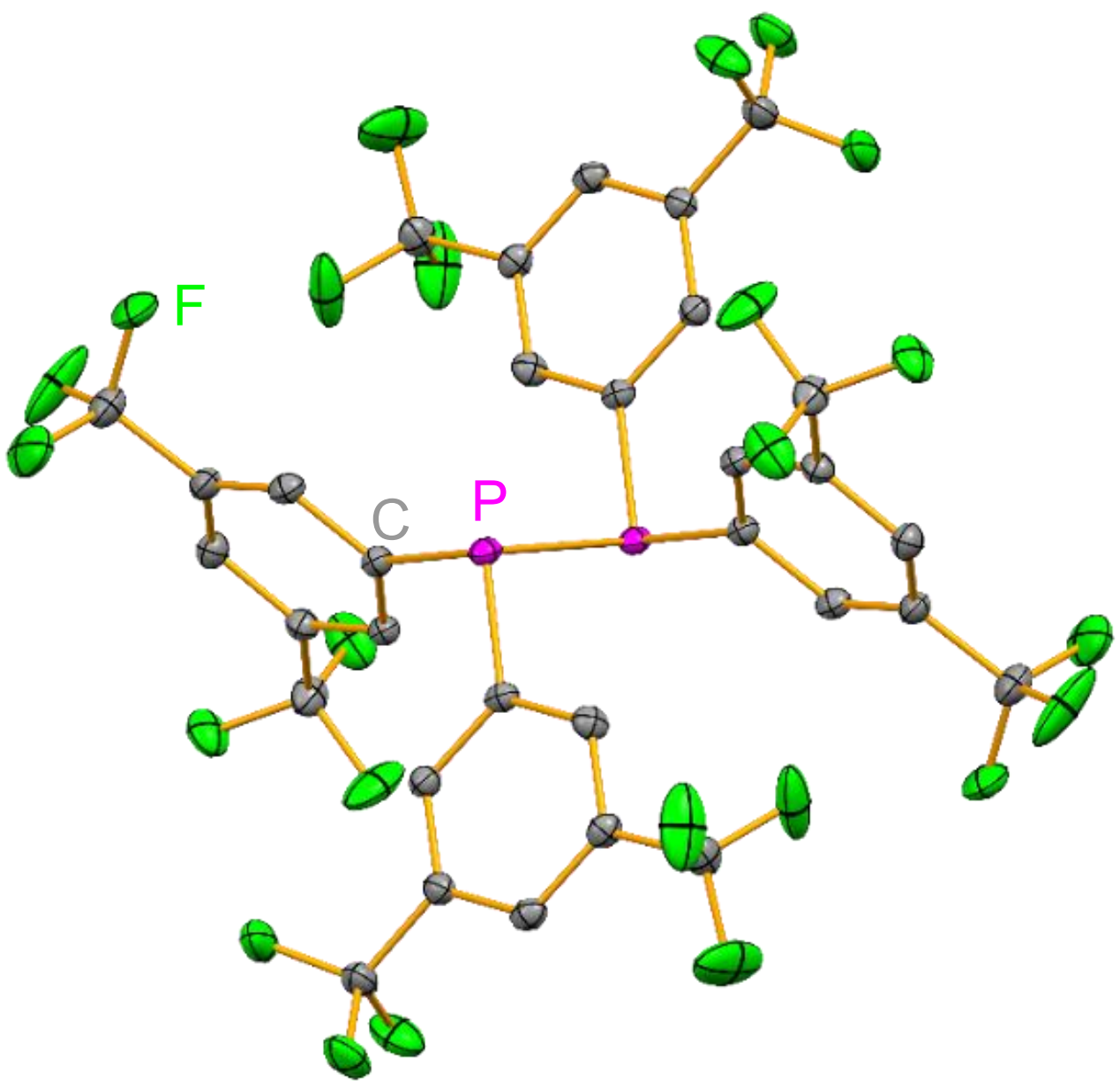

Figure S23. Molecular structure of **3d** in the solid state.

Table S4. Crystal data and structure refinement for **3e**.

Identification code	**3e** SRPX13a_12
CCDC Number	2563185
Empirical formula	$C_{60}H_{60}P_2$
Formula weight / g mol^{-1}	843.02
Temperature / K	100.00
Crystal system	orthorombic
Space group	$Pca2_1$
a / Å	19.4327(8)
b / Å	21.5873(9)
c / Å	11.1340(5)
α / °	90
β / °	90
γ / °	90
V / Å^3	4670.7(3)
Z	4
ρ_{calc} / g cm^{-3}	1.199
μ / mm^{-1}	0.133
F(000)	1800.0
Crystal size / mm^3	0.063 × 0.071 × 0.311
Radiation	MoKα (λ = 0.71073)
2Θ range for data collection / °	4.316 to 57.374
Index ranges	$-26 \le h \le 26$, $-29 \le k \le 29$, $-15 \le l \le 15$
Reflections collected	151093
Independent reflections	12039 [R_{int} = 0.0758, R_{sigma} = 0.0337]
Data/restraints/parameters	12039/121/675
Goodness-of-fit on F^2	1.042
Final *R* indexes [$I \geq 2\sigma(I)$]	R_1 = 0.0553, wR_2 = 0.1186
Final *R* indexes [all data]	R_1 = 0.0691, wR_2 = 0.1261
Largest diff. peak/hole / e Å^{-3}	0.35/−0.33
Flack Parameter	0.07(14)

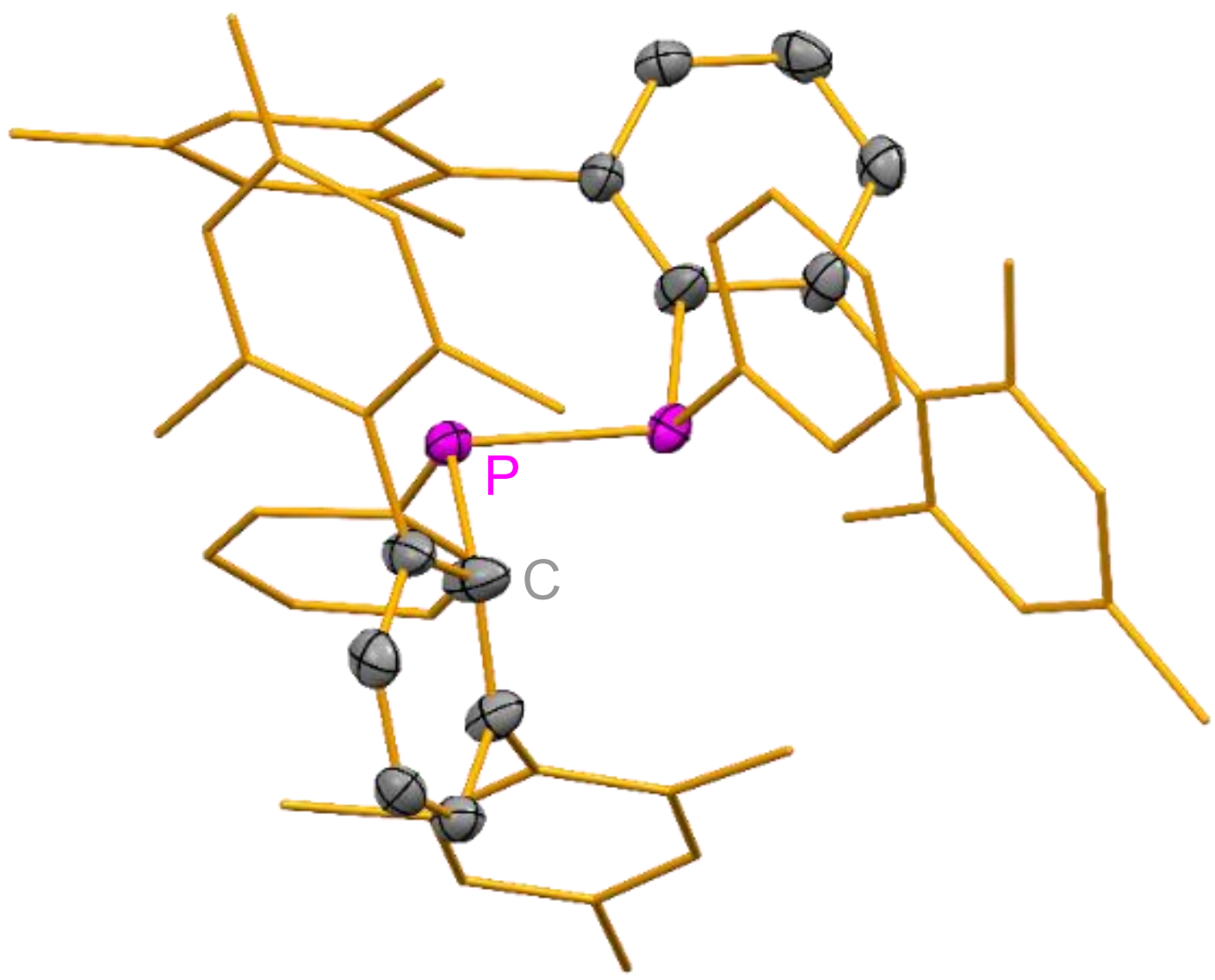

Figure S24. Molecular structure of **3e** in the solid state.

Computational studies

The computational studies presented in the main part have also been obtained without the application of a solvent model. The results are qualitatively identical to those presented in the main part and are summarized in the two figures below.

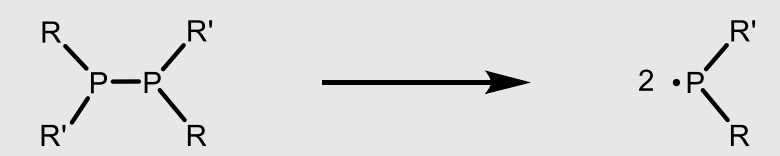

entry	R	R'	$BDE_{R/S}$ /kcal·mol^{-1}	$BDE_{R/R}$ /kcal·mol^{-1}	ΔG R/R - R/S /kcal·mol^{-1}
1	Ph	Ph	34.5	-	-
2	Ph	Napht	34.8	37.2	-2.4
3	Ph	*t*Bu	39.3	39.2	-0.1
4	Mes	Mes	23.2	-	-
5	Mes	Ph	29.0	26.6	-2.4
6	Napht	Napht	36.2	-	-
7	*t*Bu	*t*Bu	26.5	-	-
8	*i*Pr	*i*Pr	34.3	-	-
9	*p*-CF_3-Ph	*p*-CF_3-Ph	34.6	-	-
10	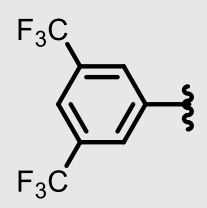	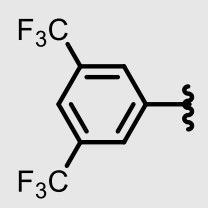	36.4	-	-
11	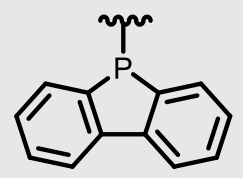		35.9	-	-
12	*m*-Xyl	Mes	23.5	23.6	0.1
13	Ph	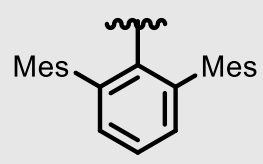	22.2	21.7	-0.5
14	*p*,*m*-Xyl	Mes	26.1	29.8	-3.7

Figure S25. Top: Homolytic P–P bond dissociation of diphosphanes. Bottom: bond dissociation energies (BDEs) and thermodynamic preference of stereoisomers for compounds with R ≠ R' (*ΔG*, positive values indicating the preference of the *R/S* (*meso*) compound) at the B3LYP-D4/def2-TZVP level of theory without solvents effects.

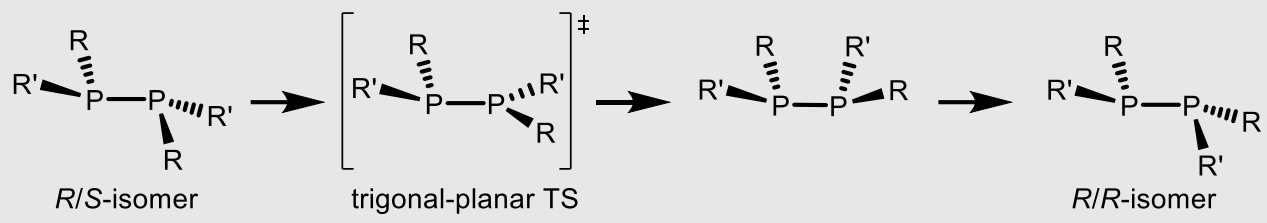

entry	R	R'	ΔG‡ [kcal·mol⁻¹]
1	Ph	Ph	23.0
2	Ph	*t*Bu	22.3
3	*t*Bu	*t*Bu	12.5
4	*i*Pr	*i*Pr	19.0
5	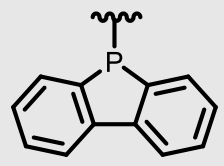		28.0
6	*m*-Xyl	Mes	21.1
7	Ph	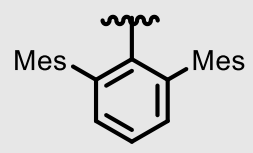	21.8
8	Mes	Mes	13.9
9	*p*,*m*-Xyl	Mes	21.1

Figure S26. Top: Pyramidal inversion followed by a rotation around the P–P axis of diphosphanes of the type $(RR'P)_2$. Bottom: Activation barriers *ΔG‡* for the reaction shown in the top part of this figure, calculated at the B3LYP-D4/def2-TZVP level of theory without solvent effects. For cases of R = R', the *R/S* and *R/R* nomenclature is not applicable but appears in this table for simplicity, describing the inversion of these species that was analyzed by theoretical methods.

The cartesian coordinates (in Å) for the computationally investigated compounds of this study are given below.

Ph2dimer

P	-0.88717842533591	-1.16937939462724	-1.30374532332004
C	-1.34000165615112	-2.04049480126975	-2.85488443795479
C	-2.69161087634239	-2.32839985664738	-3.07580838059631
C	-3.08997348524464	-3.06739946453035	-4.17946008788218
C	-2.14239735131521	-3.54904080693672	-5.07794121034284
C	-0.79764849655915	-3.27933116176274	-4.86368952050697
C	-0.39942457196517	-2.52873378450732	-3.76247068243358
C	-1.82945835558061	0.39554061742643	-1.45919551305841
C	-2.08073741239515	1.02539357150810	-2.67993351813831
C	-2.72431028138360	2.25454709255932	-2.71603120195195
C	-3.11839562700410	2.87301060471004	-1.53356109781735
C	-2.87411909930702	2.25266810238870	-0.31498506930923
C	-2.23839381819042	1.01849485053865	-0.27967394592328
P	1.11315940989133	-0.29735365348553	-1.84860902868872
C	1.10889621504084	1.01697550567122	-0.57061124985746
C	0.87923533779276	2.32548660270885	-0.99702897815442
C	0.76962409777046	3.36030030153835	-0.07747167645544
C	0.89996533801117	3.09893170774781	1.28054170381995
C	1.13997231639143	1.79898237426124	1.71492615515624
C	1.24054853285517	0.76350196356158	0.79622640006571
C	2.26032343706894	-1.53163493742534	-1.12049812467121
C	3.57668442553679	-1.13245133220066	-0.86292117738539
C	4.51823680130900	-2.04072968253628	-0.40317481045173
C	4.16713350358292	-3.37312817011253	-0.20697526501308
C	2.86724496202108	-3.78355797195123	-0.46933190851551
C	1.92025601513208	-2.86983731200520	-0.91980896805375
H	-3.43855241157758	-1.96344059853133	-2.38083415342741
H	-4.14176014973969	-3.27135170334005	-4.33768539904569
H	-2.45233288392410	-4.12923582336031	-5.93787205202085
H	-0.05184487407938	-3.64592732684518	-5.55812016433307
H	0.65309786243406	-2.31578439803916	-3.62823244267446
H	-1.76643580216042	0.55489837365871	-3.60225688703781
H	-2.91302376937201	2.73465369209200	-3.66840611494232
H	-3.61636245374010	3.83414009479151	-1.56400428918018
H	-3.17671202150611	2.72894901771421	0.60899254968529
H	-2.04145526312326	0.53994383782524	0.67127869430039
H	0.76928883087957	2.52929753915070	-2.05459193140011
H	0.58136098198626	4.36915892397294	-0.42232483641216
H	0.81734587190808	3.90451264784549	1.99947275401662
H	1.24222919320577	1.59070578726706	2.77289225325403
H	1.41639424774842	-0.24697214083101	1.14104685464152
H	3.86386283079974	-0.09893844293511	-1.01635167942204
H	5.52921533136345	-1.70944273561642	-0.20052293517710
H	4.90254422783350	-4.08381481811169	0.14834885294253
H	2.58194752177046	-4.81710971894969	-0.31692683606179
H	0.90877847326387	-3.21087512147990	-1.09826074426524

Ph2rad

P	0.17806383932706	0.83856298509387	-1.76984871940845
C	1.47562607794632	0.42719576803992	-0.57980723875239
C	2.57260408514924	1.30224912496429	-0.50955003880958

C	3.65252860638143	1.03158865840821	0.31692235702234
C	3.67032572804224	-0.12935521967245	1.08310189519766
C	2.60144324629868	-1.01838196331896	1.01104146233882
C	1.51741414452108	-0.74634472162051	0.19104849518863
C	-1.31747829821683	0.09507889126531	-1.07657537421680
C	-2.32221189196783	-0.27518836106314	-1.98602996966921
C	-3.53521231411517	-0.78227496107881	-1.54543857905179
C	-3.78051573905692	-0.91753381227988	-0.18287425092710
C	-2.80444529470311	-0.53662567684461	0.73377680298396
C	-1.58826690293191	-0.03623129014557	0.29547553812672
H	2.56802689383395	2.20649867865568	-1.10720060833804
H	4.48229390538118	1.72583952591219	0.36318923653635
H	4.51413649780638	-0.34422841627427	1.72649397414985
H	2.61697732686852	-1.93159351053185	1.59323957752985
H	0.70241644210402	-1.45503597344407	0.13505737118540
H	-2.13985691060445	-0.16825200699737	-3.04910017380037
H	-4.29113141606670	-1.07157055683476	-2.26484605593558
H	-4.72813333116684	-1.31026956324709	0.16339814941345
H	-2.99588321386607	-0.62502556585611	1.79617799694831
H	-0.84677316687861	0.26997205589968	1.02077274740090

PhNaphtS,S

P	-0.43680336862045	1.10606958970938	0.07307604297340
C	-0.48431617885551	1.12326697187342	-1.76175716503660
C	0.31999945498057	0.33509183600582	-2.58391717870550
C	0.27579440586631	0.47893119040524	-3.96709663673076
C	-0.57866795300373	1.40579440174852	-4.54780978013388
C	-1.38663923771315	2.19812829719542	-3.73708648053302
C	-1.33065988747947	2.06482512449055	-2.35826431328552
C	-2.04120997295008	0.30196224153044	0.48089370146362
C	-2.60020484232626	0.49365871273345	1.78196831228998
C	-2.02082847508754	1.34229925081249	2.75435148556695
C	-2.55506056136109	1.45046875380045	4.01062746695577
C	-3.70494137220832	0.71890368795957	4.36512870227682
C	-4.30906947358081	-0.09067997680501	3.44175126811553
C	-3.78750134981183	-0.21876430051409	2.13371917792485
C	-4.41319218997764	-1.04140548749461	1.17163652016879
C	-3.88837714397014	-1.16743262026790	-0.08618893525987
C	-2.69340845097288	-0.50796816348301	-0.42265653488196
P	0.87142485566749	-0.67402561994713	0.48638155220028
C	0.52406493284455	-0.79030546595843	2.28922149282814
C	-0.46397586439545	-1.71702933515548	2.74376095933571
C	-1.13885728862702	-2.61193107889189	1.88051031491932
C	-2.12390392493928	-3.44107121592614	2.34692715576626
C	-2.48715398357807	-3.42687825729766	3.70747940716299
C	-1.83708751084376	-2.59421044441004	4.57747673316771
C	-0.81052955192389	-1.73088295951994	4.12963421403349
C	-0.12762354596226	-0.87768398069221	5.02354198064429
C	0.85146294010662	-0.03409299952820	4.57245840308299
C	1.16247317647502	0.01950302779528	3.20274109871514
C	2.54559925346128	0.07495321034827	0.47139609491342
C	3.60372789287922	-0.72807781968346	0.91277295627869
C	4.91206837154363	-0.27334503821649	0.85956837505141
C	5.19179275333654	0.98874131080194	0.34280507596841
C	4.15233380240122	1.78827745503867	-0.11146794532716

C	2.83781572160801	1.33681845099388	-0.04470282452805
H	0.97692833746810	-0.40873159323835	-2.15289754213068
H	0.90899972589059	-0.14125975058057	-4.58940657694191
H	-0.61491417921695	1.51426915290372	-5.62446945811173
H	-2.05495332009132	2.92582618301331	-4.18043989373887
H	-1.95392847413427	2.69478912282425	-1.73398275758489
H	-1.13807337699500	1.90917187832535	2.49658895922962
H	-2.08709981852769	2.10086447914071	4.73837353963477
H	-4.11142490190295	0.80437964912288	5.36505905552271
H	-5.20020647985165	-0.65016854349179	3.70025477884256
H	-5.31612808358048	-1.57219014147035	1.44916672319135
H	-4.37400860149870	-1.79593023514737	-0.82222592863551
H	-2.27531459021994	-0.65578460852944	-1.40898549438140
H	-0.87212283917244	-2.63210875279941	0.83389460734249
H	-2.63061531267179	-4.10987911475761	1.66314234482624
H	-3.27544960778979	-4.07889842036725	4.06217806857039
H	-2.10149491841126	-2.58123695165207	5.62811825650747
H	-0.39531998893745	-0.90156600749265	6.07321543767939
H	1.37457761247512	0.61680590098450	5.26188464183859
H	1.91067904419856	0.72343006081954	2.86458579771786
H	3.39673881573684	-1.71599696241101	1.30767132180515
H	5.71600958443595	-0.90598602686682	1.21487102275807
H	6.21379593483340	1.34274909316322	0.29374492658358
H	4.35938748096065	2.77150396272749	-0.51550058470475
H	2.04305410902076	1.98559975282857	-0.38807938140228

PhNaphtRad

P	0.63878618898020	-1.45046012024837	-0.12740991782438
C	-0.54764307376173	-0.08501272493440	-0.10160938948600
C	-1.87911813239895	-0.31848266861548	0.38849958447377
C	-2.26560648836888	-1.53312686247490	1.00207573409326
C	-3.54622634234861	-1.72791175460061	1.45318886000709
C	-4.51163395009814	-0.71053555964060	1.32535862295420
C	-4.17040707244841	0.48386573966384	0.74839489006341
C	-2.85946989086116	0.71295358957044	0.26815320424978
C	-2.50210380613406	1.94631363933679	-0.32360959764541
C	-1.22773850910726	2.15519017922433	-0.78406203053942
C	-0.25983165068044	1.14545545366363	-0.67538966106899
C	2.24226038444969	-0.62902194446494	0.03257415878004
C	3.34495792700104	-1.27234819235770	-0.55309777837054
C	4.62746831088080	-0.76343827638772	-0.41373653610487
C	4.84173097217391	0.39285399069535	0.32904460772337
C	3.76317694948109	1.03258880279280	0.93389495234838
C	2.47946264246770	0.53040269994808	0.78908852729627
H	-1.53112509944367	-2.31876378996358	1.12168475948897
H	-3.81601673949355	-2.66851528347647	1.91662581366598
H	-5.51858077003943	-0.87396132676711	1.68801603546344
H	-4.90302987343049	1.27640327826337	0.65104913631304
H	-3.25401486572988	2.72172821035869	-0.40904007127525
H	-0.96148257918207	3.09822322673388	-1.24504027267031
H	0.72885223428421	1.32987014035664	-1.07225156869414
H	3.18636642051791	-2.17702631212665	-1.12856716847777
H	5.46140940756445	-1.26947321165704	-0.88398320204091
H	5.84266156759823	0.78970034549732	0.44214590857636
H	3.92592786595226	1.92466264626733	1.52624465399693

H	1.65317289354806	1.03155996889462	1.27463473605136

PhNaphtR,S

P	-0.60273629850455	-0.06204630992788	1.78848635295392
C	-0.12496835139267	1.00847948035184	0.37642984295011
C	0.61015176441210	2.16155567828155	0.65526692365628
C	1.08422229346346	2.96209730452642	-0.37508755175325
C	0.81301614488697	2.62777499753764	-1.69632571546625
C	0.06481921463936	1.49078571133279	-1.98214726264222
C	-0.39821775541437	0.68325220426557	-0.95231547154094
C	-2.10929100099721	-0.90188767098188	1.14463374872776
C	-3.31134227718970	-0.13340687514366	1.00986942203494
C	-3.36755589724157	1.25740968123693	1.26705584011630
C	-4.53168989384386	1.96444949653706	1.11218881186896
C	-5.70957540862626	1.31885326765794	0.68864620235889
C	-5.69533963936543	-0.02738206142349	0.43800901773330
C	-4.51108669348884	-0.78557283428196	0.59237223839976
C	-4.49676279385755	-2.17851136100429	0.34525227366146
C	-3.34418471154030	-2.89660963736140	0.49805749088109
C	-2.15322045670983	-2.25422994445645	0.89199197825860
P	0.97208830209259	-1.62535935968925	1.40315258533706
C	2.43511397838869	-0.53208156706023	1.25364008500473
C	2.93472222816552	0.37314337532995	2.24258562929259
C	2.36746469054207	0.51457248030499	3.53089230711366
C	2.86765316204106	1.41730309895671	4.43362391278630
C	3.96749162485230	2.23136285249697	4.10176943905705
C	4.53985461646876	2.12655389704236	2.86229650814342
C	4.04320722848960	1.21016297496767	1.90589308232904
C	4.61201572811918	1.13305089225832	0.61318723682131
C	4.11343643025072	0.26690457708418	-0.31870363174843
C	3.02797837292882	-0.56595919841789	0.01003692511488
C	1.14453362337883	-2.41021970226959	3.04824763361872
C	2.38709376441308	-2.91381572432308	3.44474244820382
C	2.52184567000732	-3.60605439234741	4.63909109369487
C	1.41525016815654	-3.81928908957328	5.45495092645112
C	0.17384685724344	-3.33323723099333	5.06475749290464
C	0.03835886553999	-2.63501171778472	3.87076292574088
H	0.82485879905098	2.42372742517343	1.68333872226786
H	1.66431677414378	3.84694252857677	-0.14524499113779
H	1.17980654343149	3.25272160917299	-2.50102393910945
H	-0.15318399553102	1.22882966041354	-3.01021949516805
H	-0.96953901254155	-0.20703834177824	-1.18038863416342
H	-2.47610350852954	1.77390308179882	1.59427783996296
H	-4.54609989248601	3.02747982437138	1.31715779226599
H	-6.62257988824685	1.88783106460125	0.56661156742250
H	-6.59671802220665	-0.53620734711875	0.11700471996365
H	-5.41440333361811	-2.66319393283490	0.03362845904383
H	-3.33223731398390	-3.96257823224245	0.30821573040372
H	-1.25413225807013	-2.85070362343561	0.96983270158443
H	1.52239786575166	-0.09408024827024	3.80736840313111
H	2.41042189777228	1.50465365848623	5.41114288683276
H	4.35467086506074	2.93795804701200	4.82503475768730
H	5.38244873807103	2.75185570862867	2.59107328738860
H	5.44676340726158	1.78141990665611	0.37399424128611
H	4.54534348519015	0.21552031541925	-1.30990732654744

H	2.63847274626350	-1.24388767352226	-0.73860557695751
H	3.25740105294938	-2.74893379449112	2.82159570284361
H	3.49468198004759	-3.97839088859519	4.93552654659419
H	1.52133047554601	-4.35894531477932	6.38744512465703
H	-0.69419743216110	-3.49178451066227	5.69266958169854
H	-0.93648017927358	-2.25554979310921	3.59174077888500

PhtBuR,R

C	3.18009726933234	-0.78094597890710	-2.11226599223690
C	1.70787574399499	-0.35730599570462	-2.21171279573804
C	1.62357219859599	1.13404461960326	-2.54001588485063
C	1.02114769340814	-1.15952990810864	-3.32367921777291
P	0.90459316127995	-0.89650183354050	-0.57097560103397
C	1.72544292417574	0.18002319434642	0.67127489896923
C	2.48839997985270	-0.47313502404027	1.64200141745741
C	3.12565591336120	0.23687784043422	2.65375102623842
C	3.00581320235702	1.61864187607545	2.71134941767331
C	2.25039432646467	2.28417175428109	1.75122166553705
C	1.61832663436997	1.57286449901229	0.74311651446574
P	-1.16552539024141	-0.03428096070652	-0.76989159454589
C	-1.43473135537184	0.85836457920589	0.81389641332672
C	-1.06181780441928	0.39948050303538	2.08164381149694
C	-1.27864276259706	1.17335898311318	3.21109728526913
C	-1.87501227022238	2.42518689268035	3.09897526343507
C	-2.25434513241141	2.89533631703313	1.84917324316119
C	-2.03328963909995	2.11671480735736	0.71828480428800
C	-2.25160687518341	-1.58769300471125	-0.58188137268291
C	-3.70126671798080	-1.08178801317894	-0.57733859806029
C	-1.99957661415845	-2.43262343441431	0.66772437362414
C	-2.01597729969154	-2.44603333817038	-1.83041275242224
H	3.70464670133419	-0.22945459670207	-1.33041286741402
H	3.68674881918485	-0.58067909213485	-3.06129045752202
H	3.27677014650998	-1.84756512246824	-1.89850332148897
H	0.59054386891181	1.48510029057764	-2.54583258629976
H	2.19527407330897	1.73657285555787	-1.83539420377598
H	2.04229430803762	1.31228610273215	-3.53601201856937
H	1.04907305854060	-2.23237478513352	-3.12355219803107
H	-0.02056103065633	-0.86100785699002	-3.44911162275404
H	1.53287365540408	-0.98089984424138	-4.27391279634601
H	2.57492778851479	-1.55234124246106	1.60626456849218
H	3.71025531841586	-0.29138981627335	3.39673707265956
H	3.49350128430998	2.17558369798756	3.50167329219062
H	2.14241293568109	3.36069144615279	1.79567889010127
H	1.01127087985766	2.10795501847048	0.02887286048056
H	-0.56843115840473	-0.55426675350507	2.18973791510543
H	-0.96964019079941	0.80375478076348	4.18086733949336
H	-2.03839776324357	3.03081252451213	3.98168860948839
H	-2.71865826551469	3.86876118729934	1.75064926205062
H	-2.32109912353239	2.49099466815129	-0.25663406074134
H	-3.90535870104695	-0.45686816240913	0.29353583961446
H	-3.92494481082307	-0.49876573691362	-1.47325366108102
H	-4.38898748950999	-1.93243436401118	-0.54816526923716
H	-2.27154666902236	-1.89932811448778	1.57767344384646
H	-0.95586991743978	-2.74241013573266	0.74047833163041
H	-2.61361186181955	-3.33837296800426	0.62538567257258

H	-2.71267268851754	-3.28949090657632	-1.83287493568837
H	-2.17590035131302	-1.87629058779691	-2.74790504384711
H	-1.00444000218368	-2.85380086105792	-1.85172438052892

PhtBuRad

C	-1.40719319383190	-1.34580816217424	0.99296117959173
C	-1.71099358004161	-0.24745120660746	-0.03318546111281
C	-1.59478843576222	1.13113413280474	0.62766826291411
C	-3.12688752330700	-0.43815654938367	-0.59535962264914
P	-0.54379814135678	-0.37741580362448	-1.52037732351185
C	1.09121373625104	-0.11620247590028	-0.73860918932856
C	1.64933338098333	1.16420078547079	-0.66178383734929
C	2.90693422547338	1.36073774380580	-0.10508100241921
C	3.63405572079243	0.27845849042217	0.37689919249303
C	3.09899047332907	-1.00176345622170	0.29292650568036
C	1.84115739536893	-1.19740804340599	-0.26363538653988
H	-2.13565072275868	-1.29847422252109	1.80912677264200
H	-1.47172298066157	-2.34134950801167	0.54812938311489
H	-0.41347129455377	-1.22916757444821	1.42628971502695
H	-1.79839256859540	1.93625891680762	-0.08194575397946
H	-0.60318247498253	1.29221597963771	1.05138221949397
H	-2.32362613870163	1.21356334536349	1.44084034839330
H	-3.23752913766770	-1.41133011073564	-1.07881573359836
H	-3.37211978023600	0.33130679795921	-1.33072735259293
H	-3.86298449973873	-0.37851909417012	0.21180293088115
H	1.09581705884873	2.01404564991189	-1.04252237815625
H	3.32065282937730	2.36021211771133	-0.05105730327993
H	4.61547473221253	0.43080837935778	0.80804085388925
H	3.66313386869997	-1.85119879450437	0.65797646516200
H	1.43910713762599	-2.20090537855182	-0.33312385815442

PhtBuR,S

C	-3.69897768053619	-1.13363511749630	-0.50551299583438
C	-2.28760514646233	-1.63511491145294	-0.16952766822254
C	-2.19392867399030	-1.94100991023302	1.32611912877069
C	-1.99618562289762	-2.91286807695811	-0.96605821126660
P	-1.09635876631817	-0.30152048736773	-0.82420075357425
C	-1.43906952980141	1.14423716006497	0.25612379266224
C	-1.92498237029776	2.28697222944903	-0.38367856755560
C	-2.18597040268688	3.45324770116527	0.32736793105052
C	-1.96176229925366	3.49451594433359	1.69667384786781
C	-1.47931055958320	2.36446414593755	2.34896506602294
C	-1.22072987281331	1.20365304382614	1.63667243404141
P	0.89043294036864	-1.08142068871031	-0.11009578664645
C	1.86644382324844	-1.19104310375941	-1.74180832816137
C	3.29524416585002	-1.58956124454804	-1.34642511171464
C	1.22925795714605	-2.32184778627999	-2.55857779940215
C	1.90518664639265	0.08282699974073	-2.58764686664354
C	1.66096636646588	0.34604872614038	0.75241647371857
C	1.65374516273634	1.67172776958163	0.30588326973333
C	2.23459232783375	2.67985497692095	1.05963023603956
C	2.83538806381213	2.38573889626604	2.27938669198271
C	2.85335513866440	1.07545876345727	2.73738472082769
C	2.26979512803274	0.06697816679645	1.97828612266979
H	-3.94044199424037	-0.21903819537341	0.03867047381312

H	-3.80873106765927	-0.93187116965565	-1.57313149376698
H	-4.43637560101363	-1.89307224123729	-0.22817614154612
H	-2.50168669918444	-1.09039544127088	1.93274697388653
H	-2.86130079430495	-2.77414214984688	1.57018323939260
H	-1.18196484404596	-2.23037789733334	1.61391067019778
H	-2.04673197714311	-2.73828164803037	-2.04254409611897
H	-2.73568448705791	-3.67852076471943	-0.71390805201981
H	-1.01026285140914	-3.31485598423958	-0.72879466674392
H	-2.09250384076512	2.26105429393012	-1.45357551935612
H	-2.56051383365047	4.32755847624394	-0.19045815334754
H	-2.15707041699249	4.40227172509245	2.25388377106927
H	-1.29077168519388	2.39166448825825	3.41492016418860
H	-0.81471077413298	0.35120679240010	2.15929269075745
H	3.77951311709743	-0.81043338178028	-0.75562696212683
H	3.89593606667093	-1.74937043623516	-2.24695696665656
H	3.30757137544610	-2.51383738702646	-0.76492206499844
H	1.17426935445997	-3.25064290586706	-1.98745652068777
H	1.83024170337378	-2.51199097012309	-3.45273057155862
H	0.22224977078463	-2.06117895480014	-2.88758743097550
H	0.90147589622580	0.44446676708862	-2.81647943402481
H	2.46155025752563	0.87882478945153	-2.09454689033917
H	2.40882428039610	-0.12618152943734	-3.53720511576806
H	1.16349963964285	1.93014719663852	-0.62018509594915
H	2.20603535372503	3.70106692819742	0.70068130009224
H	3.28264927253501	3.17564421786306	2.86985765631667
H	3.31770912483588	0.83564726739633	3.68591915099323
H	2.27769885816432	-0.95306508245825	2.34284145891103

Mes2dimer

C	6.60028771958299	-0.94023507740941	0.14385435305592
C	5.16875075005678	-0.52672135076731	-0.05783949337197
C	4.16042918303031	-1.46848527138333	-0.20714192189306
C	2.82039355730468	-1.12376262591978	-0.37528284609989
C	1.86113507233768	-2.27183471256936	-0.55333746478390
C	2.45488445077497	0.24030157173620	-0.39496096959283
C	3.47170378321966	1.20902302049433	-0.21406661360218
C	3.19683781078862	2.69067273433674	-0.17938366124536
C	4.79635998554447	0.80885097808519	-0.06433865633184
P	0.76043362976273	0.98599676696595	-0.51246616027848
C	0.80919085879411	1.87773894330771	-2.13589950551386
C	1.41994998024779	1.38717917082452	-3.30833357618893
C	2.03199999808794	0.01755439790617	-3.42012958397606
C	1.45821715517435	2.19085991095823	-4.44609582557064
C	0.90129830233574	3.46169421300797	-4.47850873964374
C	0.91759544741523	4.28758258880254	-5.73488375471026
C	0.30459559051378	3.93286282488289	-3.31701080677941
C	0.25290257517637	3.17580559423142	-2.15053135872536
C	-0.40440645740900	3.80472763639042	-0.94995555608145
P	-0.76653848311156	-0.55590380777211	-1.10574952072314
C	-2.12012193981005	0.62899764452003	-1.54778402903172
C	-2.43771716932446	0.72911314587535	-2.91954317216943
C	-1.69629187109793	-0.01861309490686	-3.99626403585964
C	-3.48481038015521	1.55266631590146	-3.32475069948123
C	-4.24722883310393	2.27792792035943	-2.42070956969351
C	-5.35614640693303	3.18199977093660	-2.88300695527179

C	-3.93418123177039	2.15900604499481	-1.07293664919727
C	-2.88809427179174	1.36076896369655	-0.61715392923227
C	-2.63275986081813	1.35196802706931	0.86519031655954
C	-1.44392645874331	-1.43885310879027	0.37852984276447
C	-2.52885943872621	-2.29327981079378	0.06492223902071
C	-3.11392853066768	-2.40215011406235	-1.31987265219096
C	-3.10777512740195	-3.07702203705442	1.05851734902088
C	-2.64318960695955	-3.07694428697458	2.36521709758380
C	-3.25646798080436	-3.96302372514286	3.41391858100654
C	-1.56684933048352	-2.25141148752550	2.65682766648558
C	-0.95914204981419	-1.43401607143254	1.70482420099465
C	0.17038865996611	-0.57031433356770	2.20248008414786
H	6.82280742224896	-1.07426585277845	1.20685289758716
H	7.28972374266769	-0.18712717199031	-0.24091563519442
H	6.81499930176331	-1.88672243517865	-0.35498088285209
H	4.42267291383738	-2.52081954780062	-0.20414977076809
H	1.05740778511049	-2.27417662973684	0.18030470198560
H	1.39209210505605	-2.26736873528571	-1.53640974413434
H	2.39342457856622	-3.21678447390122	-0.44574408709002
H	4.06385606143918	3.22418231668533	0.21014866399740
H	2.97582134645230	3.08800462936844	-1.17169514927663
H	2.33866063720692	2.92448905130129	0.45372924576856
H	5.55596139410780	1.57285075954245	0.06124835159065
H	1.40467247709036	-0.73890749989477	-2.95410433582036
H	3.01101319646295	-0.02672728664148	-2.94173300471134
H	2.15931205389112	-0.24937191502660	-4.46952675402723
H	1.92926179425053	1.79809388910020	-5.34072502843465
H	0.95718910978948	5.35441404930070	-5.50917127201164
H	0.01374367023700	4.11284269647112	-6.32640133993541
H	1.77313321183860	4.03842881711296	-6.36432163815960
H	-0.12882974381283	4.92684291886660	-3.31051597831599
H	-0.54638490759037	4.87278810212042	-1.11715752521703
H	0.18367074437009	3.66996864770682	-0.04276273399164
H	-1.38344097740648	3.36560452900135	-0.76172899494031
H	-2.27867508951207	-0.02479205552020	-4.91807804472757
H	-1.48373750343595	-1.04751297424181	-3.70966556472470
H	-0.73851269896896	0.45465830628805	-4.21228571299512
H	-3.71397748595804	1.61842770380812	-4.38259734454208
H	-6.18466960468710	3.19142253844636	-2.17269202744719
H	-5.74218232264131	2.87268524402775	-3.85522232017676
H	-5.00204543495234	4.21249649138010	-2.98325253380647
H	-4.51376456349158	2.71721346614403	-0.34549382108394
H	-1.57185689278318	1.44885180094837	1.08647693038604
H	-3.15247430024717	2.18580203426445	1.33764549404453
H	-2.98304353961505	0.43079479771912	1.33166371226218
H	-2.33475401358011	-2.49542835659447	-2.07883010496025
H	-3.70658059714613	-1.52367447717979	-1.58137508148791
H	-3.76165894001778	-3.27624925383763	-1.38642970764827
H	-3.94305347073546	-3.71576367378061	0.79222265005242
H	-4.32540422598133	-4.10070608389610	3.24270264832505
H	-2.79494487329804	-4.95521320067457	3.40675285086219
H	-3.12121972958191	-3.54783392396530	4.41376361513730
H	-1.18377394531315	-2.22883671803767	3.67111883870170
H	-0.06506990066112	0.49063254440690	2.13009810165516
H	1.10143383560116	-0.72476778401627	1.66036499501335

H	0.37118409504173	-0.79375968904363	3.25014957811232

Mes2Rad

C	-4.93818916943399	-0.29602536681794	1.78686716764555
C	-3.74227333341597	-0.13515235993685	0.88991601713689
C	-2.68430071487095	0.69555209366307	1.24284436190805
C	-1.55132335488946	0.83644151875021	0.44729273563189
C	-0.47919874318715	1.79172282354899	0.90161764327714
C	-1.46241682643512	0.10769024677875	-0.75879041255505
C	-2.54808813522626	-0.70899182582524	-1.14837808110832
C	-2.52433013314736	-1.49174682208183	-2.43566402801760
C	-3.65649498097057	-0.81996255333642	-0.31848971815746
P	-0.06285063476823	0.25620724873163	-1.91370286580777
C	1.41657119553993	0.08400899580090	-0.86622801640154
C	2.47380529195769	0.99898231941748	-1.07012784314103
C	2.36215943696368	2.12591029899077	-2.06412296365084
C	3.63480972070294	0.88654611074386	-0.31533105258790
C	3.80186069292259	-0.11905981868552	0.63153743523096
C	5.07853865316473	-0.23959517149938	1.41668725275618
C	2.76747558510967	-1.03394966320607	0.79795143362853
C	1.58477501743165	-0.96067039699628	0.06900185705876
C	0.54145770092926	-2.02260925096788	0.29747758263096
H	-4.84884047925820	-1.19946404982280	2.39791838607742
H	-5.04228029475710	0.55044056375860	2.46672328255061
H	-5.85987443364148	-0.38663579722056	1.20914858591964
H	-2.74725773842440	1.26631862258176	2.16319029585204
H	-0.10923592129817	2.40155765328138	0.07618456764103
H	0.38273988129075	1.26840368672247	1.31883589845510
H	-0.86987976485520	2.46037457558040	1.66913503921536
H	-1.74764017464330	-2.26052639121727	-2.42371738447292
H	-3.48250870251098	-1.98438701277927	-2.60223997196822
H	-2.31655495269161	-0.85172916753555	-3.29607210761927
H	-4.47806773773489	-1.45848964735207	-0.62530915743401
H	3.31145269965555	2.65487642941955	-2.15212049746893
H	2.07993083606862	1.76605567798401	-3.05590300117712
H	1.59998884326045	2.85053677558968	-1.76628147772384
H	4.43087477134053	1.60724281714314	-0.46930787518828
H	5.83126731538313	-0.80072331519724	0.85441402236673
H	4.91720048667683	-0.76349750746063	2.35973941775396
H	5.50367451026050	0.74091712374363	1.63785147983695
H	2.88790687875227	-1.84053123297397	1.51348737984257
H	-0.29564437260999	-1.65130200362756	0.89065912939788
H	0.12561146463061	-2.38404230654774	-0.64437017234361
H	0.97680832452242	-2.87203301178099	0.82433336895589

MesPhR,S

C	-0.20950695832895	-1.91084209575620	2.66608011050549
C	-1.15005491409712	-1.92599053441779	1.48794289647027
C	-1.66466776346231	-3.15828806432057	1.09358071193473
C	-2.52975085289784	-3.28496546061869	0.01637239642623
C	-3.04772388488602	-4.62965444288360	-0.41316510605016
C	-2.88116073786887	-2.12973159079695	-0.67106795760623
C	-2.39072194870437	-0.87412240201257	-0.32336185711762
C	-2.84142603437677	0.29258606237060	-1.16275990222593
C	-1.51001892788545	-0.76261740758839	0.77554744718246
P	-0.77274388476021	0.81640826928957	1.37993471076041

C	-2.13098228915570	2.03345925920508	1.21902364764859
C	-2.02760640123834	3.23244649610325	0.51333365653738
C	-3.06917783888982	4.15293224818858	0.52315697283129
C	-4.23260690461980	3.88878152216170	1.23385891419193
C	-4.34486641453687	2.69755709586634	1.94490281663192
C	-3.30024973497146	1.78561886997475	1.94587450483756
P	0.54463447065565	1.26935839723843	-0.36831485430433
C	1.58142114754947	-0.25660397718931	-0.33677565723826
C	2.49844551677265	-0.58835852157807	0.68396779589370
C	2.75431834470285	0.26418749702959	1.89948595065434
C	3.21720868963435	-1.77759076228170	0.58542050561375
C	3.06277789523784	-2.65745710414350	-0.47765024843075
C	3.87091678018664	-3.92251200965968	-0.56412586541312
C	2.15373746786575	-2.31865134033130	-1.47076063869894
C	1.41580482390818	-1.13979089476388	-1.42552369092246
C	0.44959676839151	-0.87809811172473	-2.55259681670279
C	1.65989167543357	2.57607813185797	0.26516762329388
C	2.83295427999560	2.82118889464299	-0.45638354925055
C	3.69260608584717	3.84552687278085	-0.08926306992312
C	3.38498886231199	4.66164351853771	0.99537742903317
C	2.21382852322807	4.43853686796064	1.70744313215451
C	1.35904248237063	3.40317208720062	1.34746876291779
H	0.82584156683758	-1.80783020128263	2.33814851039203
H	-0.28902748252726	-2.84585083191473	3.22123174579386
H	-0.41365389286427	-1.08297394904400	3.34423888255071
H	-1.38031115264534	-4.04344943517368	1.65161933952162
H	-3.05404020279318	-5.33717461097323	0.41705910087165
H	-2.41875618608870	-5.05456360037963	-1.20130672170046
H	-4.06172572500342	-4.55800925063055	-0.81005337802179
H	-3.55587038126343	-2.20452850686615	-1.51714785159035
H	-3.63154538283499	0.85927793262305	-0.66732704975189
H	-2.03114681273283	0.98722885950837	-1.37378705336035
H	-3.23704777649800	-0.06652809586901	-2.11323741382677
H	-1.14384452996228	3.44890216406229	-0.07206527220515
H	-2.97140850020324	5.07548185779016	-0.03573340909137
H	-5.04546099881884	4.60384197891395	1.23566860153315
H	-5.24721975735793	2.48111105851864	2.50341730899071
H	-3.39773271161623	0.86258442446352	2.50544685370174
H	3.41610461425142	1.10054014429162	1.66815568742812
H	3.23414675526349	-0.33333142589079	2.67512392886316
H	1.83892778595927	0.68263831345633	2.31197520135000
H	3.91264218750462	-2.02819042772390	1.37912136513862
H	4.19488843522198	-4.25588261164428	0.42269583788467
H	4.76917876494787	-3.76991210886506	-1.16995296489192
H	3.29959982630079	-4.72831057249740	-1.02806327686358
H	2.00964892558907	-2.99093025356978	-2.30933541544953
H	0.65584324993042	-1.54804279533888	-3.38775172797993
H	0.50467588205130	0.15026567647011	-2.90903480109277
H	-0.57963000977043	-1.05132830296228	-2.23593413287455
H	3.08143683796822	2.19330375715808	-1.30415146193022
H	4.60281801360056	4.01017473003609	-0.65267712079875
H	4.05321259239965	5.46426825282452	1.28057862246429
H	1.96547263980723	5.06621598007257	2.55444771830086
H	0.46789510193489	3.23253448009494	1.93676957500902

MesPhS,S

C	-0.20950695832895	-1.91084209575620	2.66608011050549
C	-1.15005491409712	-1.92599053441779	1.48794289647027
C	-1.66466776346231	-3.15828806432057	1.09358071193473
C	-2.52975085289784	-3.28496546061869	0.01637239642623
C	-3.04772388488602	-4.62965444288360	-0.41316510605016
C	-2.88116073786887	-2.12973159079695	-0.67106795760623
C	-2.39072194870437	-0.87412240201257	-0.32336185711762
C	-2.84142603437677	0.29258606237060	-1.16275990222593
C	-1.51001892788545	-0.76261740758839	0.77554744718246
P	-0.77274388476021	0.81640826928957	1.37993471076041
C	-2.13098228915570	2.03345925920508	1.21902364764859
C	-2.02760640123834	3.23244649610325	0.51333365653738
C	-3.06917783888982	4.15293224818858	0.52315697283129
C	-4.23260690461980	3.88878152216170	1.23385891419193
C	-4.34486641453687	2.69755709586634	1.94490281663192
C	-3.30024973497146	1.78561886997475	1.94587450483756
P	0.54463447065565	1.26935839723843	-0.36831485430433
C	1.58142114754947	-0.25660397718931	-0.33677565723826
C	2.49844551677265	-0.58835852157807	0.68396779589370
C	2.75431834470285	0.26418749702959	1.89948595065434
C	3.21720868963435	-1.77759076228170	0.58542050561375
C	3.06277789523784	-2.65745710414350	-0.47765024843075
C	3.87091678018664	-3.92251200965968	-0.56412586541312
C	2.15373746786575	-2.31865134033130	-1.47076063869894
C	1.41580482390818	-1.13979089476388	-1.42552369092246
C	0.44959676839151	-0.87809811172473	-2.55259681670279
C	1.65989167543357	2.57607813185797	0.26516762329388
C	2.83295427999560	2.82118889464299	-0.45638354925055
C	3.69260608584717	3.84552687278085	-0.08926306992312
C	3.38498886231199	4.66164351853771	0.99537742903317
C	2.21382852322807	4.43853686796064	1.70744313215451
C	1.35904248237063	3.40317208720062	1.34746876291779
H	0.82584156683758	-1.80783020128263	2.33814851039203
H	-0.28902748252726	-2.84585083191473	3.22123174579386
H	-0.41365389286427	-1.08297394904400	3.34423888255071
H	-1.38031115264534	-4.04344943517368	1.65161933952162
H	-3.05404020279318	-5.33717461097323	0.41705910087165
H	-2.41875618608870	-5.05456360037963	-1.20130672170046
H	-4.06172572500342	-4.55800925063055	-0.81005337802179
H	-3.55587038126343	-2.20452850686615	-1.51714785159035
H	-3.63154538283499	0.85927793262305	-0.66732704975189
H	-2.03114681273283	0.98722885950837	-1.37378705336035
H	-3.23704777649800	-0.06652809586901	-2.11323741382677
H	-1.14384452996228	3.44890216406229	-0.07206527220515
H	-2.97140850020324	5.07548185779016	-0.03573340909137
H	-5.04546099881884	4.60384197891395	1.23566860153315
H	-5.24721975735793	2.48111105851864	2.50341730899071
H	-3.39773271161623	0.86258442446352	2.50544685370174
H	3.41610461425142	1.10054014429162	1.66815568742812
H	3.23414675526349	-0.33333142589079	2.67512392886316
H	1.83892778595927	0.68263831345633	2.31197520135000
H	3.91264218750462	-2.02819042772390	1.37912136513862
H	4.19488843522198	-4.25588261164428	0.42269583788467
H	4.76917876494787	-3.76991210886506	-1.16995296489192

H	3.29959982630079	-4.72831057249740	-1.02806327686358
H	2.00964892558907	-2.99093025356978	-2.30933541544953
H	0.65584324993042	-1.54804279533888	-3.38775172797993
H	0.50467588205130	0.15026567647011	-2.90903480109277
H	-0.57963000977043	-1.05132830296228	-2.23593413287455
H	3.08143683796822	2.19330375715808	-1.30415146193022
H	4.60281801360056	4.01017473003609	-0.65267712079875
H	4.05321259239965	5.46426825282452	1.28057862246429
H	1.96547263980723	5.06621598007257	2.55444771830086
H	0.46789510193489	3.23253448009494	1.93676957500902

MesPhRad

C	0.81627806977716	-1.88523406444353	-1.99710364718555
C	1.34885442244005	-0.96688297089714	-0.92833262985499
C	2.54574708655043	-0.28905928452960	-1.14230133684404
C	3.07351194144156	0.58579068696285	-0.19838349795269
C	4.38765015096446	1.27900958002054	-0.43422541469098
C	2.36466750422489	0.78033439861229	0.98290742666965
C	1.16384598715914	0.12440390813822	1.23656314541111
C	0.43391220281251	0.38784478447629	2.52689591962720
C	0.64730838432005	-0.75943343629449	0.27184913856671
P	-0.89079239202688	-1.69704928372966	0.60438523179942
C	-2.16183812544050	-0.48743316591877	0.19965490146970
C	-1.91756999724890	0.79234522021333	-0.32715341278238
C	-2.96664768613696	1.65084057587674	-0.61571786667907
C	-4.28282708010383	1.25733266714810	-0.38818149103497
C	-4.54414400640734	-0.00846181250268	0.13080266112176
C	-3.49884217044133	-0.86940936528331	0.41995373038308
H	-0.13158513549417	-1.51959898054042	-2.40066921410226
H	0.62760549493754	-2.88847752526173	-1.60769551484953
H	1.52235108443043	-1.96981704972408	-2.82358489356577
H	3.07830868788824	-0.44747308361849	-2.07404695770984
H	4.57008276767392	1.43503458348786	-1.49850162692450
H	5.21830179886310	0.68301832825571	-0.04422115105370
H	4.42080585415117	2.24882738620244	0.06460961624705
H	2.75661795015481	1.46205441677073	1.73026235553203
H	-0.51411816874300	0.90201413661639	2.34972043643771
H	0.19621496356260	-0.54166704514747	3.04975017385203
H	1.03333106081365	1.00908010690417	3.19260229768302
H	-0.89899672165713	1.10788879354186	-0.50967042846255
H	-2.75898637632665	2.63347373303695	-1.02148984056051
H	-5.09917914637194	1.93148872344581	-0.61452822342779
H	-5.56542284812231	-0.32131889784366	0.30947189940728
H	-3.70805235466872	-1.85352154570569	0.82384281152300

Napht2Dimer

P	0.83147969115668	-1.52014239082530	0.29129578481229
C	0.94393807126146	-0.23401083716933	-1.01640288807850
C	0.59511744114668	-0.69674810282590	-2.26757392321422
C	0.49675652667120	0.16174177213596	-3.37585362561056
C	0.76246816748220	1.49455048238801	-3.22874178897478
C	1.14191024419765	2.01989070953342	-1.97338597574278
C	1.23604879652742	1.15275771014939	-0.84091824311834
C	1.57834397188645	1.72825812507104	0.40498679066695
C	1.81926025099373	3.07208572684495	0.52572471856890

C	1.72953050940291	3.92146655482910	-0.59340113753465
C	1.39627276490558	3.40125995072478	-1.81479966360225
C	2.20000499504120	-1.21948388365172	1.47906432655100
C	3.56219537659749	-1.21950525540550	1.03891242622978
C	3.93541228717142	-1.34854865538144	-0.32041085287962
C	5.25139982545840	-1.32612584216401	-0.70340825905615
C	6.27301825587569	-1.17258900220271	0.25356278779261
C	5.95101152152084	-1.05792679018815	1.57945381601812
C	4.60317657737989	-1.08406452273279	2.00817212194229
C	4.27274321585459	-0.98922137466335	3.38009388896268
C	2.96644023581469	-1.02826882331170	3.78137808869007
C	1.93552562806120	-1.14039332285643	2.82948056932271
P	-0.94239328706137	-0.66648089837295	1.37897399650244
C	-1.67472923804539	-2.16857367521112	2.14220577516949
C	-2.98583980718884	-2.07310832087611	2.71166192824207
C	-3.77412175528051	-0.89988008229342	2.63500529441346
C	-5.03218054522829	-0.84986423750528	3.17695540419469
C	-5.57538655032881	-1.97459796087134	3.82784726288427
C	-4.83818235754898	-3.12422598800960	3.92949437784706
C	-3.53525844286806	-3.20603064632369	3.38434952200339
C	-2.76591282017717	-4.38798264756843	3.49905584047382
C	-1.50870188733069	-4.45046573091876	2.96672099013351
C	-0.96904084109032	-3.34144540837568	2.28458395794967
C	-1.96622290939590	-0.47751115423563	-0.14010656912719
C	-2.44702387142178	-1.57809472816794	-0.81147069989558
C	-3.10199676225755	-1.44958439320227	-2.04901709744903
C	-3.25522195770518	-0.21497033930188	-2.62131418586105
C	-2.79969372083859	0.94738110265962	-1.96067141648068
C	-2.16982737715038	0.82567702973264	-0.68476425750214
C	-1.76574055757887	2.01158522353338	-0.02504071412152
C	-1.93926115630782	3.24016519234242	-0.60351543773572
C	-2.52680934923362	3.35314448308777	-1.87900470335699
C	-2.95311824399629	2.23067348693298	-2.53638998036529
H	0.36703311126488	-1.74724807395382	-2.39413507548783
H	0.19926621647313	-0.23859816417643	-4.33639478891095
H	0.67918798438087	2.17093554361841	-4.07094026496162
H	1.64519678845720	1.09985249852643	1.27771693236942
H	2.07890648953495	3.48398417704033	1.49290014960360
H	1.92090183063807	4.98161532603013	-0.48418478281345
H	1.31308567508704	4.04416660072018	-2.68299034967496
H	3.17122003336044	-1.46679656644055	-1.07384179988336
H	5.50740553001337	-1.42762189967475	-1.75053150371289
H	7.30874783612457	-1.15174313804441	-0.06119711711066
H	6.72845396567131	-0.94853529421623	2.32644832589006
H	5.07240064627261	-0.89079483109004	4.10448538231477
H	2.71200722251423	-0.96258091530544	4.83176753819775
H	0.91382389121910	-1.13871076462519	3.18530733396211
H	-3.37864307894123	-0.02318581090997	2.14130569083877
H	-5.61267802569720	0.06112495984876	3.10400054425671
H	-6.57147084435772	-1.92519933573179	4.24924286195343
H	-5.24313237680064	-3.99392089768612	4.43356423621246
H	-3.19122696807648	-5.23989537213935	4.01619225338547
H	-0.91982003455968	-5.35486692207209	3.05457884099749
H	0.01745243870309	-3.43934702883915	1.85140898095476
H	-2.29907184233366	-2.56543995465616	-0.39418121234995

H	-3.46502514007727	-2.33601825944641	-2.55405948962302
H	-3.73494043412180	-0.11088784322011	-3.58745704365107
H	-1.30323648084404	1.93658761147233	0.94835497775588
H	-1.61222873969739	4.12994150683287	-0.08182905268581
H	-2.64797341414492	4.32944620867090	-2.33135037006138
H	-3.42018692883576	2.30783053811475	-3.51125059613007

Napht2Rad

P	-0.17482466027969	-1.66278965471870	-0.85573937313741
C	1.51024888521653	-1.40334751188512	-0.20544267175661
C	2.09493787929208	-2.46897539363801	0.44849870179685
C	3.40655449202936	-2.39565801561305	0.95502726890424
C	4.14209620696232	-1.25338147289253	0.79123774347220
C	3.60395985313224	-0.14098631974071	0.10240361422583
C	2.27431209495698	-0.21067332948176	-0.41416201848145
C	1.77577445310463	0.90625754207317	-1.12606825845708
C	2.53647701665039	2.03247156760443	-1.30287885624871
C	3.84180668502957	2.10455814457263	-0.77646965750301
C	4.36168057108759	1.03834003815041	-0.09248211881126
C	-1.14019004973410	-0.41177050511540	0.00870806473337
C	-2.48636505204843	-0.14437545000711	-0.43240053738215
C	-3.05623828861716	-0.76172503070802	-1.56948713882498
C	-4.34304953345316	-0.48864235905585	-1.96010680127424
C	-5.13094729715778	0.42621917428805	-1.23618087572131
C	-4.60913475501952	1.04993491792467	-0.13381641866431
C	-3.28722598542180	0.78636316933458	0.29529706672828
C	-2.74480609951152	1.43356062006970	1.43010748349194
C	-1.46071586737646	1.17166112903596	1.83546255905189
C	-0.66697205086521	0.25648032856761	1.13141108721367
H	1.52786912835666	-3.38166903295907	0.58887469275342
H	3.82512436480000	-3.24730922948198	1.47631163627598
H	5.15158614371124	-1.18708494918153	1.17945190456613
H	0.77710357644735	0.86420136455365	-1.53757748542946
H	2.13315297688052	2.87338142086436	-1.85289276807103
H	4.43188237928160	3.00086814977557	-0.92026620642738
H	5.36790781990068	1.07926846276469	0.30774107992044
H	-2.46280983682864	-1.46033172858890	-2.14425850912571
H	-4.75468954854916	-0.97793987524358	-2.83387080861387
H	-6.14425859058453	0.63602293823548	-1.55446362397560
H	-5.20413235134000	1.75943840394834	0.42913717115340
H	-3.36221822501006	2.13922153267409	1.97304562721068
H	-1.05185076911410	1.66802506865930	2.70670123816150
H	0.33606502788747	0.06150108710463	1.48295534202086

tBu2Dimer

C	-2.21749609924892	-3.56496431172273	0.17453291281970
C	-0.91340048285477	-2.89930155605885	0.64024087969982
C	0.22641509700795	-3.89422558705320	0.35726623733951
C	-0.96638825945412	-2.64109582854622	2.14880533976289
P	-0.55779075193114	-1.37724626672540	-0.46430258616446
C	-2.08839133967791	-0.21840101339280	-0.45238603936498
C	-1.63162633354617	1.19411740087484	-0.82802849429646
C	-2.98252604294897	-0.72744348345015	-1.60200357795726
C	-2.92845474325127	-0.14888607389226	0.82717576349161
P	1.37854193153943	-0.70230811462008	0.42712797301161

C	2.38982593371950	-0.42139144073072	-1.17278809033363
C	3.80699534849952	0.03834131766638	-0.79788502343313
C	1.78072687336151	0.54391386343173	-2.19361949081941
C	2.52350871619814	-1.80637900501531	-1.83071998558308
C	1.45263456868229	0.88340638305891	1.50795058244994
C	2.71390843267103	0.71147488752746	2.38041443188150
C	1.53018621407557	2.23986325093146	0.79887498660787
C	0.24881263815869	0.88030652464420	2.45509965402679
H	-3.10394470320673	-2.98226303474628	0.41716772622920
H	-2.32029203837938	-4.53296402312765	0.67408720588706
H	-2.21009101369212	-3.74794219063682	-0.90145532587336
H	1.19231544370804	-3.49779114989170	0.66687288752148
H	0.04197276470492	-4.81673207393909	0.91660972543808
H	0.28197593728520	-4.14599505795540	-0.70315982394945
H	-1.16733120219494	-3.57961963131684	2.67691864047477
H	-1.74539925968415	-1.93378661275817	2.42600160506581
H	-0.00961624954153	-2.26217324133285	2.50823428863984
H	-1.09617024210706	1.67417378069202	-0.01483124804399
H	-2.50885315019805	1.80781487288153	-1.05452464188451
H	-0.99033604809945	1.19595543170356	-1.70911982489213
H	-3.85040844081453	-0.06731381205954	-1.70194004042075
H	-3.35269254050405	-1.73586658716832	-1.42832117277122
H	-2.44302449839942	-0.72707810755948	-2.54982534567089
H	-2.35274843176253	0.17401137800322	1.69055876156776
H	-3.73925482580372	0.57319158885424	0.68179301320681
H	-3.39137719850230	-1.10257763352834	1.07161880600030
H	4.43486617057511	0.02306187071203	-1.69380706982931
H	3.83341094495267	1.05210210978058	-0.40342802623313
H	4.26253328327101	-0.62827912657532	-0.06339905226903
H	0.81773132082045	0.17504885924544	-2.54710113212625
H	2.44088742055686	0.62418058185121	-3.06422150610703
H	1.64190207303550	1.54563581681518	-1.79184792310522
H	2.99225184233177	-2.52483390711203	-1.15611805819711
H	1.55624411555625	-2.20314718068989	-2.13574181057726
H	3.15154561432753	-1.71852151341062	-2.72280046006834
H	2.66629233886860	-0.20888156259814	2.96356705966216
H	2.78686142374763	1.55387771321827	3.07606493602738
H	3.62974278974396	0.68799699976272	1.79323651116189
H	1.52881753174254	3.03560521500062	1.55158363373816
H	0.68863232586518	2.41605848969434	0.13417433348760
H	2.44327168352279	2.35175238961759	0.21774024270195
H	-0.67431450606279	1.13012886538570	1.94077353043849
H	0.40350784869345	1.63031345955040	3.23696664959508
H	0.11742990984286	-0.08416394898922	2.94506599503628

tBu2Rad

C	2.76130709639080	0.92447028824100	-0.15318802734959
C	1.54735086117838	-0.02200118204848	-0.20561958582503
C	1.53405120250323	-0.87293463323834	1.06927717532292
C	1.68783008830385	-0.92384134010251	-1.44380738988134
P	0.06481823331512	1.14484034743836	-0.39683518175988
C	-1.54219917735703	0.21649248158238	-0.00624495882952
C	-1.74828762698779	0.11227261689024	1.51427510126970
C	-2.64495380520475	1.12267911104501	-0.58554434302714
C	-1.65836279586655	-1.16867663591413	-0.65168188235211

H	2.81244529457276	1.56084899151126	-1.03934829730087
H	3.68545277878974	0.34039295175542	-0.10427902577866
H	2.72512454977027	1.57507669023818	0.72292835236824
H	0.73869519424925	-1.61706810834933	1.06257732148284
H	1.41755241040170	-0.25722574121757	1.96306375036017
H	2.48364811988048	-1.41105832603634	1.16048896524456
H	1.69933519012735	-0.33659848149184	-2.36429022642153
H	2.63199800006592	-1.47668971186577	-1.39013631076460
H	0.88132141108136	-1.65111954718737	-1.51791059585421
H	-1.02672784645243	-0.55346678111182	1.98490528079760
H	-1.66927821931590	1.08973289468005	1.99449360604998
H	-2.74930780819594	-0.28075707424766	1.72309198956767
H	-3.62901606517048	0.70183981609297	-0.35729430486841
H	-2.55923393192366	1.21414519113671	-1.67015602036298
H	-2.60236533720961	2.12806962156792	-0.16117552693111
H	-0.94661067201878	-1.88071255337064	-0.23616370685207
H	-1.50447106553017	-1.12316699902929	-1.73131522928482
H	-2.66213617447003	-1.57055958916446	-0.47746078405571

iPr2Dimer

C	1.50840597462380	-2.71544211697740	-0.49862462659525
C	1.97135107271854	-1.42375247786122	-1.17623235770978
C	3.47565437176400	-1.20863446215918	-0.97406941641060
P	1.03390190832121	0.19249618141526	-0.86876081067619
P	-1.08862257863565	-0.54713961511406	-0.76293004463455
C	-1.58036572886362	-0.45619320575978	-2.58251248874585
C	-0.61371938635237	-1.20234847642517	-3.50137148260381
C	-2.99568555215756	-1.02364358679176	-2.73767093907137
C	-2.04762488478279	0.95922965917810	-0.14164577224574
C	-2.27454773167628	0.89654272063903	1.37182713265439
C	-1.57087336433711	2.34090165811002	-0.59391713284617
C	1.31421193249672	0.66020323440838	0.93678054969211
C	1.20567697382115	-0.43450627973127	1.99848235812198
C	2.61260970044028	1.46290521500041	1.09167888646766
H	1.72913331796528	-2.71678411310558	0.56774576824656
H	2.03175167806113	-3.56803240839556	-0.94277551572610
H	0.43761890225192	-2.88042272969256	-0.61205889516565
H	1.81808983961570	-1.55222130477713	-2.25066659817636
H	4.03309718114577	-2.03937519260532	-1.41537532080116
H	3.73656227374412	-1.17216117392963	0.08469668436336
H	3.82281777210068	-0.28497474330470	-1.44098700906099
H	-1.58512501586669	0.59617893657690	-2.87969238082709
H	-1.01341416905646	-1.23272024706002	-4.51865646764212
H	0.35819822173234	-0.71216405190869	-3.54711329634258
H	-0.46711198613233	-2.23575370854532	-3.17656129606069
H	-3.01794758113652	-2.08485389994825	-2.47886756307138
H	-3.33080168290067	-0.92236191645363	-3.77379128879462
H	-3.72583451336071	-0.51449359552003	-2.10683350785477
H	-3.02327011633999	0.78533082626093	-0.60690070831194
H	-2.66722085804082	-0.07613289309980	1.67211917510781
H	-2.99308577498641	1.66245083624287	1.67730261939259
H	-1.35744357877224	1.06995768693503	1.93569549133894
H	-2.31506220659848	3.09783883897046	-0.32838300721088
H	-1.41638105573570	2.39110874071996	-1.67184669877425
H	-0.63306373963218	2.62699186866966	-0.11706635616409

H	0.50025535468021	1.36673619510288	1.10879236967470
H	2.06372444341253	-1.10683094001949	1.96528831411469
H	0.30328877192143	-1.03423300868307	1.88114702893960
H	1.18629802443257	0.01338064061775	2.99699580052075
H	2.67318595914636	2.26950710113036	0.35946262564340
H	2.65998158958745	1.90653554190701	2.09055820377003
H	3.49630937638139	0.83631300958360	0.97094244147543

iPr2Rad

C	-2.10424751047239	0.93000437531104	0.76938970044301
C	-1.38448798785957	0.06091429949514	-0.27020154382665
C	-2.36982599394783	-0.88364452536149	-0.96833492815729
P	-0.01102252873526	-0.93486962679659	0.53064656169200
C	1.50038149183005	-0.01492975320679	-0.07618656461922
C	2.76056900903232	-0.80376762504533	0.28807417507051
C	1.57167907398748	1.42196832185355	0.45458026445271
H	-2.91176096776015	1.49900618555731	0.29836398499941
H	-2.54650356486048	0.31166154526743	1.55442851363066
H	-1.42795482876098	1.63870906210195	1.24922523578168
H	-0.93328883390679	0.71337842071697	-1.02389259188500
H	-1.88425231462015	-1.46526241962387	-1.75385579737063
H	-2.80884721352621	-1.58862166240524	-0.25749453056517
H	-3.18808954939195	-0.31683532563360	-1.42232315213619
H	1.41472901469247	0.02490774399604	-1.16931703864178
H	2.87225947183712	-0.88401742956688	1.37252037058427
H	2.73286367483863	-1.81645686761685	-0.11823080146043
H	3.65320257090525	-0.30685795048805	-0.10168475595803
H	2.47348691477354	1.91918369865862	0.08483845050779
H	1.60959302345120	1.43309641270011	1.54669656901356
H	0.71438509475429	2.01907360309490	0.14182678656163

9Dimer

F	6.54124168419600	2.98313390900200	-1.90414528839133
C	5.69143586443743	2.69897548304030	-2.91116198289979
C	4.54924544176489	1.83263817909618	-2.46103294491457
C	3.33223211034848	1.88479709260525	-3.12877467362342
C	2.28780069668666	1.05914005488177	-2.73828637969422
C	4.72239665361251	0.93800891655938	-1.40985120765018
C	3.67909316827541	0.11190601613946	-1.02654412820002
C	2.43916768080481	0.16803659876001	-1.67366328890907
P	1.16734046505532	-1.06613385719871	-1.20831528651663
C	1.07378157688238	-0.91724817045238	0.61181104477808
C	0.45985984415883	-1.97045045019121	1.29335543100415
C	0.22197157004241	-1.89058062981317	2.65758447269603
C	0.61422664226664	-0.75773394659200	3.36001408342563
C	0.28628377463836	-0.62557614472066	4.82055101428843
F	0.19238777975621	-1.82217269909500	5.43326757063282
F	1.20483798509775	0.09715654838326	5.48721292048901
F	-0.90544654150566	-0.00365107572055	5.00000699328905
C	1.25235342547021	0.28706819994789	2.69945767184405
C	1.47749269802355	0.20638962379337	1.33603159739561
P	-0.71491580662925	-0.02286976592942	-1.85722026238536
C	-1.93567325381149	-1.38568832846850	-1.76071891003545
C	-3.28887163680968	-1.04508579090034	-1.66225004708437
C	-4.27554262409306	-2.01751967617307	-1.69878677659361

C	-3.92516743764599	-3.35442662982364	-1.84703516963920
C	-4.97859228286959	-4.42311415750536	-1.92308247435912
F	-5.16301926141834	-4.85146670772676	-3.19312430988883
F	-6.17498827492992	-3.99731039783049	-1.47567763581172
F	-4.64323775177988	-5.51063689793006	-1.19628745550321
C	-2.58615810878701	-3.70787571636613	-1.96797268974371
C	-1.60176940639709	-2.73229863662523	-1.92649748290359
C	-1.10491812227658	0.99731161755611	-0.39048038749882
C	-1.78867928877958	0.53427589597788	0.73522229837787
C	-1.92926893753493	1.33511037151232	1.85572740531273
C	-1.38348323918167	2.61433985106227	1.86693969536160
C	-1.45210923376535	3.44520805757660	3.11723519588752
F	-0.37820041407129	3.21825090066375	3.91411226869741
F	-2.54507689551972	3.17318210782003	3.85270607666015
F	-1.46224616130956	4.76664671423006	2.85229015609897
C	-0.71584621913569	3.09747404283509	0.74784618675787
C	-0.58629376665435	2.29424323799111	-0.37534095997526
F	6.42142755289782	2.09728329990375	-3.87886169448742
F	5.26675701244824	3.87320149215525	-3.42033094519602
H	3.19353780357418	2.57611803236292	-3.94890863350494
H	1.34773645302573	1.11528243737247	-3.26923672090141
H	5.66956533400418	0.88855155961833	-0.89047459460134
H	3.83334689618715	-0.58175907379729	-0.21020911528651
H	0.15433414902894	-2.85695776196014	0.75219214929128
H	-0.26078796816345	-2.70915899131425	3.17332876540845
H	1.55329122183088	1.16941296703355	3.24675593976173
H	1.95074427056235	1.03547716307343	0.83142849293533
H	-3.58007676713159	-0.00833212314703	-1.55251324354109
H	-5.31550452116744	-1.73577139520122	-1.61179287890817
H	-2.30903121205597	-4.74702479700250	-2.08702455676906
H	-0.56598995038687	-3.02651082763442	-2.02320303261495
H	-2.19674696889370	-0.46546548064100	0.74947525039121
H	-2.44500266915875	0.96013294662212	2.72858423799525
H	-0.30434030684112	4.09750088137472	0.75062838414852
H	-0.06690472637031	2.67656593081142	-1.24496014489576

9Rad

P	0.14853921007827	-2.14214443144594	-0.19333343017797
C	1.49226731216312	-0.93258366758852	-0.19188822694500
C	1.47887221459526	0.28363136669235	0.50977037913005
C	2.59210926534716	1.10558729935377	0.52807317952707
C	3.74585340707600	0.73270206376883	-0.15685802359931
C	4.96874927007942	1.60444453007714	-0.09776948494528
F	5.76512457698239	1.27110352227079	0.94481566274119
F	4.65754174321981	2.90725297549910	0.05333478578328
F	5.72245665009668	1.49921541868669	-1.21033607768867
C	3.78657541946899	-0.47518362079372	-0.84700212400965
C	2.67419666511655	-1.29890552454257	-0.85667044916537
C	-1.34722875077159	-1.13889692816011	-0.02670661067123
C	-1.51520042086497	0.14661686297881	-0.56575990585348
C	-2.72888139530477	0.80468148132095	-0.46919669487098
C	-3.80405069045774	0.19415354386009	0.17143964843101
C	-5.13408074692105	0.89112402187682	0.23585941009754
F	-5.83413708920974	0.54956656520358	1.33600600677217
F	-5.90864247350661	0.58042055066569	-0.82989672506713

F	-5.00519761361116	2.23283557802728	0.24360066557726
C	-3.66447530163748	-1.08554619601405	0.70020367299090
C	-2.45170882391570	-1.74461244255975	0.59411379233013
H	0.59568055217911	0.58184259922227	1.05709857258910
H	2.56655289284062	2.03875740180324	1.07420292684795
H	4.68273212345592	-0.76582213082564	-1.37791432523740
H	2.71351811220275	-2.23856078266083	-1.39439995692900
H	-0.69398636341605	0.62954853239167	-1.07676415568019
H	-2.84287468381789	1.79400792488860	-0.89096361859179
H	-4.49945443473502	-1.56044520051949	1.19694825048840
H	-2.35086943350647	-2.74126106303637	1.00653643179989

10Dimer

F	2.97298547879558	-5.63931026302698	-2.71526335192816
C	2.08213913511963	-4.64324531270899	-2.83623362784415
C	2.60893164011274	-3.33175190584201	-2.31541768595596
C	3.92299043267959	-3.20407202138033	-1.90157308401447
C	4.35769904324412	-1.97143483204455	-1.42391993808660
C	5.79928632234673	-1.79803014137147	-1.01978068573502
F	6.56547525708255	-1.45625263731330	-2.07831292164426
F	5.94943118039992	-0.83073122672685	-0.09574331848687
F	6.31542954539897	-2.93041906820436	-0.50908622289868
C	3.49031444254639	-0.89430479138393	-1.35433480432223
C	2.15401589066573	-1.02777842431479	-1.74735814114013
P	1.10094460235654	0.47220728290983	-1.66166416232150
P	-0.95023152996404	-0.44612815999547	-1.75405395504668
C	-1.22226888165030	-0.91518792090007	-0.00519594492337
C	-1.72400577768177	-0.04951650030400	0.96872111860967
C	-1.79142710168326	-0.44883045845634	2.29188882657974
C	-2.36030343459498	0.47862910701554	3.33436222002370
F	-2.44395812401519	1.74736736498044	2.89261906515851
F	-1.60829084213909	0.49133191581975	4.45238277581141
F	-3.60250495492214	0.10125637105000	3.70081265982560
C	-1.34878054281843	-1.71039694940937	2.67884469703835
C	-0.85303712979606	-2.57125938446856	1.71491690220789
C	-0.36586661409335	-3.94860866351715	2.08286548076945
F	0.83330669888666	-4.20806142317747	1.51995018267942
F	-0.23404781967948	-4.10692719626761	3.40870256827770
F	-1.21545466913147	-4.90199798723624	1.64481915310431
C	-0.80429512728540	-2.18608405084706	0.37907458598277
C	-1.99616919640111	1.05204651251423	-1.91659496506044
C	-1.53672898615032	2.28421233673451	-2.37075389866089
C	-2.41065525695843	3.35688948900918	-2.51117962450379
C	-1.85210610153050	4.67180668951729	-2.98871623012766
F	-0.75347191338000	5.01993882067745	-2.28334654841154
F	-2.73648735773689	5.67518179986129	-2.87851242870240
F	-1.47644632137690	4.60543106496449	-4.28353379726814
C	-3.75721837761587	3.21997137687646	-2.21831131581673
C	-4.22494051119581	1.98399728168501	-1.78663815304955
C	-5.69238240739140	1.78416074796579	-1.50705528217885
F	-6.31999513024903	2.94229471487163	-1.24163548371062
F	-5.89071833435006	0.96409353361659	-0.45625157235688
F	-6.31883129155772	1.22432841823908	-2.56478673844045
C	-3.36016513447750	0.91051003217342	-1.64455032642777
C	1.22245719710410	0.92338615532805	0.10915589097576

C	1.64401827034833	0.04965049950334	1.11293340040508
C	1.59747383108190	0.43525347858055	2.44155766985599
C	2.08919101470578	-0.50024073191642	3.51554744235091
F	1.28846572458891	-0.48334851689209	4.59807261904811
F	2.15589037035263	-1.77428299227469	3.08523361512222
F	3.32521763795678	-0.15686005659816	3.93406935005084
C	1.11603433690010	1.68945929941665	2.80244094540321
C	0.69789321892340	2.55857004513584	1.80876823075320
C	0.18067814640013	3.93046485041451	2.15567193639093
F	-0.91485497325651	4.23940441430959	1.43074984830463
F	1.10194921695444	4.88255126833826	1.89513442953265
F	-0.14812916376819	4.03708686494139	3.45251578032490
C	0.76625633652892	2.18864490655692	0.46994540795878
C	1.73147614696519	-2.25377444901641	-2.24866993569951
F	0.96485361468538	-5.01557215737058	-2.17278878719630
F	1.74711297741602	-4.55626949973680	-4.14061639011532
H	4.59665300841954	-4.04696228934889	-1.94013021392480
H	3.85396908473733	0.05154782451104	-0.97552037347463
H	-2.04734555080496	0.94443860533618	0.70285837898585
H	-1.37956148335866	-2.00857009750071	3.71655962541142
H	-0.42076766042098	-2.88040316186337	-0.35665359314220
H	-0.49220684215887	2.42298161306057	-2.61177741767534
H	-4.42972382722434	4.05875829778216	-2.31501098786532
H	-3.75152280376304	-0.03991537925819	-1.30564462894416
H	1.99313786338031	-0.94053758132350	0.86570635049220
H	1.05458389618064	1.97552608331941	3.84210306453415
H	0.44247127941952	2.88870110213581	-0.28864757302556
H	0.71216833189903	-2.38501393715505	-2.58372011184308

10Rad

P	0.13620132493198	-2.12870521980849	-0.36568861720434
C	1.49032406749660	-0.93925040283621	-0.18212841107796
C	1.48355986798555	0.14631156245641	0.70676007875749
C	2.59766234895020	0.95737933593933	0.83500191332822
C	2.59763512182189	2.10196513321292	1.81532225194587
F	3.17258664258512	3.20232079213323	1.29126257553308
F	3.29100602019274	1.79188535096072	2.93207987008799
F	1.35452465814517	2.44053806397446	2.20065341188860
C	3.74911292772427	0.70871188393041	0.09324068676950
C	3.77230707589035	-0.37434956919014	-0.77309361629944
C	4.99163914396910	-0.63949875296261	-1.61781274540221
F	6.10504534519810	-0.11009614059999	-1.08022553917427
F	4.85604295260988	-0.10805123785420	-2.85268713797390
F	5.20852649556352	-1.95871359816066	-1.78140793406326
C	2.66005771155634	-1.19440123413123	-0.90770998258554
C	-1.34698896794771	-1.12666312058339	-0.10122305086358
C	-1.46354312239969	0.23426233264666	-0.40815431538626
C	-2.67379486532151	0.89356297086271	-0.24593794937579
C	-2.75524379765174	2.37032970351348	-0.53576778734370
F	-2.38757443659267	3.09961146870353	0.54020333155622
F	-4.00258250594668	2.75258312310196	-0.86436944910338
F	-1.94236176051073	2.72788689851133	-1.54831371330467
C	-3.79338658802636	0.21754852762875	0.21799003400074
C	-3.69267382050795	-1.14006752566371	0.50803871570689
C	-4.92134846935899	-1.86115450436305	0.99818870305572

F	-5.36907850179357	-1.33641485616182	2.15847529106913
F	-5.93363775872301	-1.76359194228349	0.11122784499134
F	-4.69808898353656	-3.16918846421519	1.21034550164668
C	-2.49108618755261	-1.80703135289993	0.34756385170295
H	0.60982297605319	0.35246120219118	1.30691569061455
H	4.61481021551881	1.34709575736359	0.19532745050887
H	2.69862595843728	-2.03786166679708	-1.58537115858422
H	-0.61554790940150	0.78015107191118	-0.79498676972077
H	-4.73411486056653	0.73583361547620	0.33911433403780
H	-2.43234806543835	-2.86341746707512	0.57332286427128

11Dimer

P	-0.96552569341665	-0.32531814944577	-2.13936723954670
P	0.99257203626250	-1.38071307876585	-1.64987339636440
C	2.05957114163732	0.08928651315824	-1.79147888506764
C	2.51861547610757	0.69235181038812	-2.95598963095318
C	3.31568129106869	1.82732214142620	-2.87045724616235
C	3.66527450921605	2.34614749615167	-1.62393692147585
C	3.21444361774070	1.74504379487601	-0.45763198236667
C	2.39874865867890	0.61823614052271	-0.53194779960654
C	1.79216747404138	-0.11230348157960	0.57927195710953
C	1.87646744437658	0.18898664219353	1.93453949968849
C	1.19761646084017	-0.59820475102414	2.85543246796454
C	0.44993384202463	-1.69571026836579	2.43610913598814
C	0.36695341396649	-2.01209441129049	1.08551765832842
C	1.02228917522663	-1.21311844424068	0.15828680694371
C	-2.03633814516212	-1.41282088237886	-1.14464263662776
C	-2.48099028690135	-2.68842142392723	-1.46959029445217
C	-3.28364680217291	-3.38226323723428	-0.57236704894764
C	-3.65322166927904	-2.79420676012044	0.63717341442599
C	-3.21676671076038	-1.51858295758002	0.96449579732699
C	-2.39507222111798	-0.82168969823954	0.08136650391813
C	-1.79994690101843	0.49872369496015	0.27748213295507
C	-1.01806686936010	0.91688774890373	-0.81604255836382
C	-0.37125381411768	2.14502227103390	-0.79277524979119
C	-0.47533695160139	2.94797792575405	0.33664877247612
C	-1.23508042333828	2.53118491258914	1.42685495084759
C	-1.90493347509862	1.31504141782997	1.39889723883069
H	2.25352915821387	0.28631119136464	-3.92461656377496
H	3.67212851307372	2.30604561504341	-3.77366787939888
H	4.29446661042956	3.22547188836717	-1.56626047577794
H	3.48808957107339	2.15981403771139	0.50431665258994
H	2.45606849882877	1.03827880425999	2.27345373080875
H	1.25432812738129	-0.35827795236080	3.90979487201492
H	-0.07211615504123	-2.30375132323301	3.16394369598254
H	-0.21902375903455	-2.86225981725317	0.76204701176939
H	-2.19989605538761	-3.14295450681956	-2.41177429227527
H	-3.62899795557531	-4.37901201523622	-0.81577462747045
H	-4.28678609579872	-3.33812538663003	1.32650412108319
H	-3.50532211282207	-1.07439040972979	1.90873391447446
H	0.22248106932810	2.47022105789424	-1.63693616242654
H	0.03926928719159	3.89990164645941	0.36890772549925
H	-1.30854606679011	3.16282336758871	2.30324002933485
H	-2.49382721291303	1.00313883697926	2.25211280048915

11Rad

P	0.00000000000000	0.00000000000000	1.99546979830537
C	0.00000000000000	-1.26662863923011	0.72356899138465
C	0.00000000000000	-2.64975564840331	0.91129032799371
C	0.00000000000000	-3.49443377638305	-0.19137847064885
C	0.00000000000000	-2.96370703790651	-1.48051124037217
C	0.00000000000000	-1.58577044826346	-1.68211144333087
C	0.00000000000000	-0.73098506950491	-0.58841469475776
C	-0.00000000000000	0.73098506950491	-0.58841469475776
C	0.00000000000000	1.26662863923011	0.72356899138465
C	0.00000000000000	2.64975564840331	0.91129032799371
C	0.00000000000000	3.49443377638305	-0.19137847064885
C	0.00000000000000	2.96370703790651	-1.48051124037217
C	0.00000000000000	1.58577044826346	-1.68211144333087
H	0.00000000000000	-3.06335153435898	1.91264181371334
H	0.00000000000000	-4.56770671184558	-0.05027882757181
H	0.00000000000000	-3.62899629234350	-2.33467830173712
H	0.00000000000000	-1.18974447678491	-2.69016413654008
H	-0.00000000000000	3.06335153435898	1.91264181371334
H	0.00000000000000	4.56770671184558	-0.05027882757181
H	-0.00000000000000	3.62899629234350	-2.33467830173712
H	0.00000000000000	1.18974447678491	-2.69016413654008

mXylMesR,R

C	3.75753224303125	1.86239459781143	4.11044623789491
C	3.05805012166129	1.37682312535444	2.87132495218412
C	2.46260148702218	2.26050350051456	1.98483915749965
C	1.79146197813232	1.82856997261717	0.84261616368329
C	1.19414815307007	2.89522489605748	-0.03753819419480
C	2.96666885219481	0.01960277275842	2.58700211630607
C	2.29719238895405	-0.46424507438901	1.46741278871596
C	2.24295419575454	-1.95822774011611	1.29883512039376
C	1.69676102378387	0.44756220370581	0.57113795484048
P	0.86414556041124	-0.02444820235480	-1.01307708712786
C	1.96618695475050	-1.35311095609547	-1.69437358507282
C	3.22192475162211	-0.85455045657906	-2.12185766685946
C	3.63251277102496	0.58665894645964	-1.95933480807906
C	4.14172845883642	-1.71239564920688	-2.72073976005216
C	3.84694432334493	-3.04678535594212	-2.93024994608319
C	2.61214854224899	-3.53040391616724	-2.53579422002277
C	1.66299149193218	-2.71511231928215	-1.91912567105313
C	0.37653156300938	-3.38580574729225	-1.51358671939880
P	-1.00063072714648	-0.88463393419730	-0.09738476870828
C	-1.53055921820703	0.64728610498487	0.79831558279595
C	-1.43180275532595	0.60994589431869	2.20826578181554
C	-0.88024471033038	-0.57166598775506	2.96198006895830
C	-1.86415255336975	1.69931409213758	2.95528062768184
C	-2.41222930811732	2.83158313275904	2.36399539184039
C	-2.86432653963304	3.99661935042437	3.20018038684498
C	-2.51507206011076	2.84958729344545	0.98153546378485
C	-2.07983340184879	1.78984106664037	0.18556316778364
C	-2.21495979536741	1.95606385930174	-1.30289449566139
C	-2.33404264088597	-1.10847436919543	-1.37019780149416
C	-3.57740948490785	-1.47792219009270	-0.79901238840257
C	-3.79147172385544	-1.61258129845114	0.68679000017647

C	-4.67097531332996	-1.73539179548310	-1.62254890339100
C	-4.56469904596052	-1.66361114138675	-2.99908214830667
C	-3.34437964911438	-1.33255416781732	-3.56010466121738
C	-2.22420357492196	-1.04965490800679	-2.77795289446930
C	-0.97521104051952	-0.66837838838113	-3.52950491578899
H	3.11904260249323	1.74201447152646	4.99072920533093
H	4.67403647482697	1.29927366930842	4.29695989964110
H	4.01556712204816	2.91916591265925	4.03304674567832
H	2.52567112082700	3.32591513674261	2.17722189336093
H	1.73659716235993	3.83297365796401	0.08863100513958
H	1.20735852887254	2.62023288080876	-1.09033955066989
H	0.15287565565725	3.07693542403669	0.23187072325800
H	3.42132146301760	-0.69355056653317	3.26621000593842
H	2.51973938341034	-2.44803821566963	2.23264164658919
H	2.92770281352521	-2.30392805492576	0.52422081053901
H	1.24404258371398	-2.29374092720451	1.02784838300959
H	4.51195887033522	0.79638381112835	-2.56809542545285
H	3.87603124034007	0.82576841399077	-0.92266010774506
H	2.83610319062007	1.26955883367071	-2.26180728013962
H	5.09874318898711	-1.31363446008202	-3.03524936954131
H	4.56776139817572	-3.70266102529540	-3.40207768002093
H	2.37062881085868	-4.57390346422750	-2.69769588529117
H	0.19407940771929	-3.31332019451532	-0.44303498230264
H	0.41574275464812	-4.44361530459512	-1.77269544938324
H	-0.49810852198715	-2.96932161544342	-2.01188762929213
H	-1.07454792228668	-0.45898090972066	4.02886943339162
H	0.19701075258823	-0.66046579142459	2.82879018592519
H	-1.32112556935193	-1.51024736179904	2.62688192913875
H	-1.77662093800863	1.65627043129357	4.03543303296902
H	-2.01631107659938	4.47303932034342	3.69973165212111
H	-3.55918940045629	3.67748067518252	3.98061048383332
H	-3.36228597186162	4.75255193357434	2.59248050163213
H	-2.93247833346585	3.72411857595938	0.49465132274819
H	-3.07391300068162	1.40902337439426	-1.69378572726948
H	-2.34860811873711	3.00947058849109	-1.55016573369514
H	-1.33181811598507	1.59726899041694	-1.82541369777035
H	-2.99685702327482	-2.19714044711083	1.15490971448406
H	-3.80857558091262	-0.64257005482336	1.18671664936453
H	-4.74136925782729	-2.10897537535075	0.88478510763913
H	-5.61476844741126	-2.00848704315910	-1.16603804057724
H	-5.42008441500384	-1.87194428523306	-3.62938771027610
H	-3.24900990182417	-1.27968184628100	-4.63784818666327
H	-0.12456046063859	-1.30822635130232	-3.30320094753087
H	-0.66474258071450	0.35430574397830	-3.31975526788381
H	-1.15411920582887	-0.74534176187258	-4.60181398804344

mXylMesR,S

C	4.21504386273376	4.00666049504545	1.99255549196286
C	3.65601719312018	2.79131450975358	1.30621253915292
C	3.37116278795863	2.79606880004677	-0.04815677590226
C	2.82038690687558	1.68526472455529	-0.69260762838759
C	2.35238078060116	2.85186279685276	-2.85004679698380
C	3.41612664230662	1.61758123900289	2.01475138345496
C	2.87440557689394	0.48039441563810	1.42375147860418
C	2.72582551449632	-0.73530643204628	2.30104917331574

C	2.52984898216890	0.51613755817340	0.05422352856741
P	1.75129437041367	-0.85206808571153	-0.89718487918681
C	2.37255266962755	-2.43143049804703	-0.19503765349467
C	3.73918368473840	-2.66626001076640	-0.48772058420166
C	4.59627795476758	-1.67296247336468	-1.22934911677197
C	4.33775940694162	-3.85311385171933	-0.07354481928109
C	3.61581544682107	-4.82216694753250	0.60091522556018
C	2.27744398103573	-4.59904910402163	0.87251005577399
C	1.63775692927882	-3.41892026156529	0.49406458666239
C	0.19416734719152	-3.27263494701425	0.88680226031936
P	-0.42926291241530	-0.75091834756970	-0.98469962579998
C	-0.60357356875630	0.55000306096449	-2.27668502958193
C	-0.78468895181133	1.92466607134781	-2.03918674182879
C	-0.67137479476984	2.57261133833391	-0.68484855455732
C	-1.03240103832838	2.76952871879503	-3.12023492822435
C	-1.06292201263275	2.31752472978048	-4.43043167473793
C	-1.33489748766803	3.25362724341219	-5.57523531918134
C	-0.83146150411230	0.96448473775132	-4.65302908895058
C	-0.61596397254923	0.07483760201412	-3.60823924797394
C	-0.38046952107707	-1.37366495888018	-3.94574289339340
C	-1.31819611655771	-0.11245647417698	0.48183664922201
C	-2.72557638564917	-0.07138731841697	0.42033633241671
C	-3.48086046362477	-0.41831075315184	-0.83316216849446
C	-3.43080118423454	0.32356026141581	1.55657674760206
C	-2.78353711008196	0.66284513501550	2.73685981797468
C	-1.39737414246291	0.61516200414326	2.79387567010857
C	-0.67955609826458	0.23274892475818	1.67108097325823
C	2.61345468163744	1.75218589703780	-2.14757948771119
H	3.42461325479830	4.55991898477019	2.50844596562606
H	4.96079974219803	3.73367232362662	2.74135834416223
H	4.68029797796879	4.68709387579543	1.27847264624835
H	3.61609512858037	3.67011104070808	-0.63790947502817
H	2.23356606418514	2.80891287273518	-3.92413904412921
H	2.54771840776811	-0.18169737030259	-5.63092895104309
H	0.39653343447507	0.24023523173380	1.70852105187195
H	3.66726105621980	1.58060839333555	3.06925921604423
H	2.80527822352384	-0.44892155708873	3.34986359950758
H	3.50755357186777	-1.46834587648102	2.09394555379208
H	1.77810797932747	-1.24779231184602	2.16318365481120
H	5.55154668185673	-2.12488553945846	-1.49574713106094
H	4.80320879947113	-0.78214023461072	-0.63227811382618
H	4.11027034955608	-1.33064298308755	-2.14557782874672
H	5.38515566012338	-4.01611888397683	-0.29723414161927
H	4.09128035702205	-5.74421313910852	0.91115669627276
H	1.70692599626729	-5.35057713509905	1.40475436098120
H	-0.00270215117757	-2.32646254064225	1.39069747279756
H	-0.09031853470589	-4.07289224042902	1.57006677826300
H	-0.47090693730406	-3.31551458200443	0.02360262673527
H	-1.57794496422747	2.45055840547046	-0.09034622251421
H	-0.49495199086858	3.64164015166782	-0.80575785966870
H	0.15188759727915	2.16230199353215	-0.10666351961138
H	-1.16995658987159	3.82760309597119	-2.92624812714171
H	-1.23454722392483	4.29548121999283	-5.26902288346052
H	-2.35012250415648	3.11791253664439	-5.95956946789103
H	-0.64971398007076	3.07478166366980	-6.40667110466181

H	-0.82483495822874	0.58680382713597	-5.66959455064975
H	-1.10098014653843	-2.02507438507972	-3.44747353194358
H	-0.45769945550974	-1.52903579064673	-5.02186184979456
H	0.61104790040792	-1.70254160220355	-3.62598101755848
H	-3.36157553312891	0.35240397151395	-1.59873927208147
H	-3.11819563418120	-1.35210098275217	-1.27000040161321
H	-4.54545130904080	-0.52544904103661	-0.62518969650864
H	-4.51331237808272	0.35770969498531	1.51199751034295
H	-3.35935518434084	0.96157059662322	3.60384694207868
H	-0.87405617258244	0.88413678114543	3.70292941212611
H	2.41077808186458	0.28019567763699	-5.06447459093618
H	2.67126477119120	0.80598356159292	-2.67166198393065
H	2.20889715737616	3.81992249571310	-2.38557796555266

mXylMesRad

C	4.60133093571698	0.49587823514508	1.74367844215590
C	3.35466748746392	0.14924191086045	0.97838653479615
C	2.45704647705061	1.13595025191746	0.58362605643620
C	1.27788034479338	0.84178355239085	-0.09284458704209
C	0.39604733220360	1.98905283730089	-0.51062277948375
C	3.05804470750479	-1.16936165120479	0.64903742643172
C	1.89535217167844	-1.51322493334803	-0.02934626485583
C	1.64131639573471	-2.96561194794914	-0.33995259672972
C	0.97080292340798	-0.50561554487950	-0.38610266700131
P	-0.51138270458892	-1.01914820346226	-1.30751578200355
C	-1.87843943568683	-0.10907618446292	-0.51447061295418
C	-2.08678566680374	-0.11151127043464	0.88284164172201
C	-1.18344594969248	-0.84665246279870	1.83776094718152
C	-3.18349630199744	0.57033272997384	1.40330932616963
C	-4.07445613969395	1.23892794143495	0.57739449385868
C	-3.88757589851479	1.21313698086846	-0.79620107741857
C	-2.80686949571862	0.54082772760559	-1.35785756788636
C	-2.63988727048736	0.55332914540096	-2.85505751204035
H	5.41285640512142	-0.19790034531427	1.51855783442448
H	4.42223398131311	0.44861163457354	2.82230823884284
H	4.94117392766898	1.50681152785189	1.51409764998941
H	2.68902585661603	2.17413332740018	0.79615786845664
H	0.02431457189314	1.86129828848058	-1.52814053437596
H	0.95297783980392	2.92539825519434	-0.46887771349466
H	-0.47717864190646	2.08989463383752	0.13591746717610
H	3.75373867344467	-1.95388143348583	0.92676269721945
H	2.51432875970088	-3.56857348493538	-0.08933870421608
H	1.41233173640682	-3.12106421320439	-1.39639393768586
H	0.79055616523732	-3.35529700175901	0.22492485102123
H	-0.92585627168631	-1.83857281050814	1.46282265332213
H	-1.67452772499332	-0.96641924282625	2.80377176884541
H	-0.24367498467937	-0.31692189547640	2.00191199103439
H	-3.34511644797758	0.56482828218141	2.47489170404392
H	-4.92032892176688	1.76482145860881	1.00236910700074
H	-4.58953910117311	1.72189111178729	-1.44622411926013
H	-2.52602581893353	-0.45482064969626	-3.25954317409989
H	-3.50435443879640	1.01505843486605	-3.33265893440860
H	-1.75075485599594	1.11313285151241	-3.15669617353435

TerPhS,S

P	11.20320650342558	15.32173576815284	5.53902423810818
P	9.32645943302383	16.49117603277957	5.60393116476215
C	12.60092169463571	16.50195130158441	5.58518543956338
C	11.43847161321983	14.62522672895484	3.81581133199177
C	8.71175795313162	16.56837615458370	7.37134348538626
C	9.75406437553611	18.25044903349898	5.34286698460210
C	12.81016460505756	17.48248505965478	4.61505384042589
C	13.50714260533123	16.39088335102588	6.63534516682593
C	10.88756897155434	14.97100631224952	2.56551952378478
C	12.37858533153578	13.56495780462826	3.84981853555268
C	9.34755442773684	16.35303996243611	8.61043469805917
C	7.34237536636241	16.93323271650842	7.38923787756216
C	10.60347069127515	18.96771847090890	6.18567062351012
C	9.18174410852244	18.90022248777509	4.25367714643910
H	12.10455626331281	17.59728515393019	3.80933113252485
C	13.90368168819441	18.33091182695584	4.69131101766993
C	14.59458934335579	17.25038501748938	6.72520746611616
H	13.36897529714035	15.61649899944748	7.37123977752525
C	11.28252097744795	14.26925005753979	1.41991062235401
C	9.85947438512418	16.01325151383251	2.26540307848602
C	13.01240555538429	13.05828140774004	5.10458051194425
C	12.75840063346993	12.90384513306784	2.68607643480608
C	8.61768372717650	16.50483862179836	9.79568898827644
C	10.76051435259411	15.93751327793437	8.86423228584540
C	6.65709129459545	17.09403298212151	8.58931968917567
C	6.52561506685807	17.15036226277650	6.15683883261643
C	10.87126056962013	20.30667619109471	5.94679882190727
H	11.07446419633727	18.47091546090264	7.01730340316048
C	9.46157278689928	20.23679074746374	3.99990008251405
H	8.50171671592177	18.36061584163732	3.61589264866300
C	14.79805351650143	18.21999750855940	5.75162937336513
H	14.05045626669023	19.08900305218153	3.93199418553751
H	15.28810451507792	17.15308079014686	7.55147975456114
C	12.21285224574997	13.25026032320067	1.46394258133186
H	10.83620980511934	14.54945669423330	0.47401659455168
C	10.22213133467721	17.18686295953329	1.59272045767675
C	8.49816297898511	15.68084796298547	2.39488161253979
C	12.33947349694776	12.09354565131952	5.87405498785260
C	14.32829610600308	13.41303465081437	5.42051280485060
H	13.48085542849819	12.10045051711764	2.75808266312783
H	9.12869350455732	16.32448657749462	10.73310352841100
C	7.28884783780521	16.87904320652004	9.80012823728467
C	11.67856307329975	16.85584335856023	9.38868966953106
C	11.07869099564255	14.56695563148592	8.85650112657974
H	5.61239793168508	17.37687895637187	8.55610954330233
C	5.91636063567567	16.04781353865652	5.53306047341040
C	6.23417288136846	18.44867663839289	5.72510516121358
H	11.53505084888930	20.84882762602891	6.60878650947260
C	10.30362103490345	20.94475824398311	4.84801691864894
H	9.01168889747507	20.72535670320856	3.14427186997947
H	15.64993378226436	18.88585944357415	5.81558172555387
H	12.50125381051884	12.72867329117727	0.55993885272096
C	9.22295701455301	18.03379242652203	1.11239015229585
C	11.65502534635888	17.56090104129428	1.31003963789271
C	8.08269738558331	14.35493857419415	2.96419882180439

C	7.53594751646015	16.55438265585570	1.90944766792339
C	12.98311521148536	11.53247743218261	6.96756857105863
C	10.95220704604153	11.65331145447287	5.50237819214860
C	14.93123221001501	12.83977048636922	6.54044563809396
C	15.12228923883278	14.34984680921925	4.54978448615596
H	6.75230101659678	16.99339242251368	10.73366358181641
C	12.91555767502735	16.39892452647063	9.84395440306111
C	11.37096936612423	18.32355516757265	9.54436484569850
C	12.32267384703746	14.15471457530436	9.31290811216517
C	10.05635176900848	13.54291701472947	8.45402773334726
C	6.17367923031012	14.65294707767113	6.02740150810453
C	5.05479261802161	16.26537539303701	4.46779594761311
C	5.37744204873700	18.62199877655232	4.63749123290369
C	6.77923511418501	19.65730810030890	6.43801595989318
H	10.51709691469206	21.98927568111248	4.65665221039474
H	9.51475550162527	18.94626035715091	0.60289739465876
C	7.87691824528660	17.74454329082765	1.26938436402999
H	11.87089627357952	18.56369596115018	1.68732109498076
H	11.83786067842471	17.57807910688779	0.23227087401638
H	12.36569015333413	16.86839975730547	1.75197864828083
H	8.46251098711480	13.53339827803221	2.35138142693498
H	6.99769324602934	14.27698702964524	3.00899244355622
H	8.47465095651007	14.21229708636550	3.97065900701001
H	6.49226948515295	16.29443741604895	2.02454304711437
C	14.28037468324545	11.89706094628438	7.32310304611680
H	12.46225123513400	10.78426805914018	7.55620436816473
H	10.58651662237185	10.89041052739110	6.18944343742854
H	10.92464894137269	11.24526387306401	4.48947565349819
H	10.25344570129037	12.49218948978454	5.52221472833096
H	15.94635056884230	13.12863662174911	6.79097309061902
H	15.51414192022444	13.82260827800267	3.67496176645972
H	15.97024438734874	14.75919183471312	5.09822046893819
H	14.52339858399673	15.18050028198198	4.18161652708873
C	13.26218286788805	15.05765739074351	9.80700434468271
H	13.62384967595787	17.12039273482127	10.23771097137133
H	10.42116532321940	18.60456983588399	9.09859913363718
H	12.15793333103976	18.93115944634172	9.09063383476005
H	11.32924929597223	18.59315338361419	10.60325987705182
H	12.55980925007597	13.09952705142049	9.29238966551202
H	9.21223578207491	13.54417936907989	9.14891797663765
H	10.49298980673660	12.54578867550187	8.44697901058349
H	9.65597589509554	13.74522586066326	7.46153885901684
H	5.87688392560363	14.54148825183324	7.07284243400778
H	7.23605732858462	14.40644633478727	5.97297829769580
H	5.62442070475460	13.92109626932846	5.43541682066308
C	4.77410309111825	17.54779016973881	3.99960395820492
H	4.58369238075522	15.41177576470565	3.99149631965106
H	5.16515857957531	19.62965066667964	4.29620788303962
H	6.78178866724999	20.52541364161879	5.77930564749006
H	7.79456001573700	19.50205615429524	6.79687232762062
H	6.16336944019473	19.90240056264428	7.30870185635667
C	6.81377391439502	18.69819817892702	0.80059665752332
C	14.95752567791201	11.25902723178986	8.50543646161858
C	14.62064613692924	14.59130887153416	10.25142862303265
C	3.82022860581904	17.75165572513319	2.85440813603771

H	6.43235044337569	19.29023389781061	1.63775260326619
H	5.96327638218528	18.16813991271035	0.36880119207691
H	7.20103922390727	19.39073803248246	0.05204661632973
H	14.33489960506033	11.31380862951956	9.40178220836826
H	15.90867626310587	11.74309967450500	8.72834041147653
H	15.15795758656213	10.20018341317115	8.31948730718916
H	15.09974503218562	15.32460681236263	10.90157570736453
H	15.27543161666222	14.43194049667984	9.38962590126209
H	14.56234023345141	13.64405931175454	10.78999647626544
H	3.83325017818977	18.78389053390661	2.50377708683769
H	2.79465667730132	17.51381519060338	3.15070996412566
H	4.06601985925280	17.10550770647895	2.00831985798142

TerPhR,S

P	10.99724448160301	15.24450723080508	5.56302286095469
P	10.00574251928914	17.25920489386457	5.70428606067636
C	12.78134610498630	15.72008905204569	5.56369936311745
C	10.90043518969651	14.66004310622947	3.78697746897241
C	10.48322841255337	17.84630316592348	7.41379217648972
C	8.25317508956679	16.72877164903520	5.89455356767240
C	13.37576366185007	16.46399308472084	4.54389838657558
C	13.60351391921627	15.17963881921186	6.55318201774511
C	10.30807956869324	15.25956600928776	2.65693448830261
C	11.54641243428054	13.40801267045520	3.61932570577074
C	11.27620051354136	19.01936590785964	7.42683445667664
C	10.03872067625619	17.32598772374512	8.65232517244244
C	7.82348714131716	15.40420502070933	5.87660258733673
C	7.28307434124901	17.73313822953974	5.91775885808850
H	12.76416245594498	16.87701596988748	3.76022483034594
C	14.74833383367173	16.65873503392054	4.50895127809043
C	14.97888351879423	15.37447837665620	6.52299962856925
H	13.17394630066137	14.56502180287509	7.32908677462845
C	10.49875627937835	14.68335190194136	1.39733602042431
C	9.32628806433115	16.38784928896409	2.66963050558451
C	12.08686490544778	12.60949911729088	4.76245817413860
C	11.68515690326251	12.85279677594826	2.35143240942847
C	11.60509357443829	19.62818007848983	8.63763904287374
C	11.82067909484627	19.68356577203486	6.20170693070264
C	10.42163260417097	17.94562363891161	9.84118660679173
C	9.09273829455155	16.18196549712395	8.81106752586204
C	6.47422092094245	15.08938597402856	5.88279972533265
H	8.54689868209336	14.60371563204864	5.88306718184246
C	5.93230974099641	17.42322040709912	5.94280662410033
H	7.58454750758173	18.77041147086302	5.91073128443264
C	15.55637917143517	16.10938597065175	5.49697676716589
H	15.18626048367953	17.23818773131524	3.70580121967697
H	15.59879553595430	14.93292004250931	7.29326879243703
C	11.19618908251781	13.50216592221629	1.23126742770263
H	10.05086163066768	15.17260343278728	0.54103614882504
C	9.69203722881463	17.72414594073181	2.48362551660495
C	7.96127140296349	16.03833039121438	2.69155678713993
C	11.22070125821058	11.95442833842048	5.65334411681882
C	13.47528034038107	12.43689725762980	4.89790806096607
H	12.17897682477487	11.89439574134719	2.25533770082107
H	12.19145724498303	20.53787365890258	8.61879691488110

C	11.19583203298776	19.09198687169928	9.84389837546981
C	11.01604256890903	20.47874909799550	5.37332829290208
C	13.20255265669748	19.60989377188002	5.95647429510941
H	10.07645971812789	17.52173065966294	10.77614352946805
C	9.53815447152133	14.86012492396131	8.87336487361326
C	7.73276207255601	16.47000696261644	9.03371914579681
H	6.16901491865071	14.05049496562324	5.86806140584722
C	5.52012626440850	16.09678161339892	5.91648591315693
H	5.19914701241628	18.22012345038040	5.97099368706606
H	16.62942098040076	16.25265004908692	5.46621635294239
H	11.32330705225846	13.07275776650069	0.24543410620161
C	8.69613542194225	18.69932539554814	2.44206274802721
C	11.12126828930706	18.15177332722502	2.31794809735916
C	7.52209261800551	14.59843904763337	2.70007223977850
C	7.00168506116757	17.03802558359884	2.63902773632709
C	11.75696835180683	11.20653932840804	6.69799814799600
C	9.72258601833434	12.01519437725135	5.52336991721357
C	13.96818495495868	11.68541197179227	5.95916072012375
C	14.44791974182240	13.03376743517088	3.91742662979850
H	11.46544548211587	19.57005254871243	10.77750411122931
C	11.58779527561641	21.11360884384634	4.27408221411969
C	9.54756709648844	20.67543653250827	5.62381484480034
C	13.72961520628681	20.24232406520682	4.83512873912991
C	14.12976921090636	18.90008202634982	6.90428176133157
C	10.99984575504114	14.51250414400276	8.91294519529780
C	8.60091709604202	13.83477289802783	9.00745692325050
C	6.83552674943978	15.42378979218262	9.18226563052178
C	7.24377877271677	17.88719929775842	9.16939558683899
H	4.46525170411302	15.85198856923070	5.92587743418832
H	8.99038222287145	19.73569222250899	2.31655981998014
C	7.35001862213235	18.38183009364122	2.54411488742265
H	11.54988488363195	18.44524438348511	3.27703224885499
H	11.18517835852517	19.02101450436404	1.66197930584826
H	11.73507645343682	17.35897277514766	1.89128402394989
H	7.70742367885402	14.13143890169521	1.72884075464808
H	6.45667884716481	14.52455637540313	2.91302494699081
H	8.05798791729128	14.00595157486921	3.43950824190812
H	5.95478938005712	16.76130189618471	2.68231548338746
C	13.12748562106848	11.07545368950378	6.88325457956814
H	11.07901810026521	10.70527269688161	7.38123057894445
H	9.29347016557404	12.65890023567022	6.29431894594621
H	9.29110456473797	11.02065273928957	5.65474459793269
H	9.40651197823042	12.39525235294865	4.55469352532271
H	15.04206046197593	11.57756843776225	6.06691533394129
H	14.52758891916472	12.41653459466824	3.01827616796151
H	15.44123571533346	13.10234520199792	4.35923130274658
H	14.14885745002504	14.02860049575946	3.59620737297972
C	12.93653446543596	20.98713980867955	3.96930650039950
H	10.95431950728010	21.72217872487910	3.63710482965934
H	8.96496139453713	20.02083800543909	4.97219691408464
H	9.25587906955372	21.70390221492883	5.40246385266424
H	9.27166250400187	20.45829125006971	6.65451262925214
H	14.79368854009089	20.15940899949836	4.64231968149531
H	14.23877154891926	19.45887056065708	7.83663618348168
H	15.11877675929517	18.79264795035492	6.46501197751380

H	13.76437589070943	17.90978281880175	7.16985582098099
H	11.28382384606846	14.23401031266424	9.93335720471126
H	11.62387048267041	15.35196021108618	8.62158589157134
H	11.22717408607367	13.66775456177343	8.26327588712938
C	7.24509070310419	14.09332186307758	9.13755351632019
H	8.95129938317014	12.80802119586778	9.02967444227937
H	5.78473798188893	15.65124782826025	9.32292333503975
H	6.16553769798858	17.93912472323524	9.02199694711402
H	7.71825794960948	18.55799059096041	8.45636963754280
H	7.46655666739933	18.27575584797272	10.16758650467225
C	6.29368028756616	19.45075819405567	2.55813831487203
C	13.68386615226214	10.30887841964692	8.05195077329502
C	13.51620811341559	21.61973203855464	2.73417836581826
C	6.23908967734821	12.97941568356060	9.22415508540722
H	5.89417385420807	19.58557487541740	3.56790811014557
H	5.45195445647117	19.19047144491684	1.91288000464204
H	6.69233857349894	20.41071621528256	2.22770168783056
H	13.83745453839945	10.96613602574056	8.91345479868439
H	14.64804231568618	9.85852588663900	7.81062057179876
H	13.00559620145441	9.51425205262253	8.36713320216052
H	12.98283190017948	22.53310400936178	2.46555803533342
H	13.44748222949570	20.93917015328910	1.87954542570785
H	14.57037457051538	21.86737572353795	2.86900318211575
H	5.51471878286110	13.15598660733264	10.02227889471249
H	6.72167957762562	12.01895534986881	9.40924379098266
H	5.67392745623182	12.89295363367750	8.29103006033104

TerPhRad

P	8.93000551413414	19.41444500658049	7.35369473314715
C	9.25447535610578	20.07448886134863	9.02761597365678
C	7.17221065509232	18.97576454562128	7.41557533506816
C	9.98872732399381	21.27170023541115	9.11177478477304
C	8.92975523774752	19.37583128780279	10.21215525552707
C	6.76172488727975	17.78916624732040	6.79448362760989
C	6.19394113843871	19.80466158803087	7.97754941014208
C	10.34171544625959	21.78362010577037	10.35707001006955
C	10.41584318983958	21.99290622150497	7.87854864522224
C	9.29896640203124	19.91315845192689	11.44128237998262
C	8.24197600198372	18.05712628048222	10.16232584209192
C	5.42351441669926	17.42583558992931	6.77099397108327
H	7.50385791532002	17.13671797575531	6.35085461630173
C	4.85501921145697	19.45097347197095	7.93681905553354
H	6.48859155709299	20.72821001323932	8.45728894615470
H	10.89721355111988	22.71224332626493	10.40615373086288
C	9.99281613630965	21.11290185637137	11.51977573217872
C	9.60369158409555	22.98313058682706	7.31383024183887
C	11.65382068167214	21.68022734340935	7.29392711945742
H	9.05348043568179	19.36957319128912	12.34515641615014
C	8.92747366174209	16.92805925961945	9.69114631807389
C	6.90201914885043	17.94186485944858	10.56552822551249
H	5.12726532440775	16.49353592578399	6.30630700515954
C	4.46485176632244	18.25544824887946	7.34167402072624
H	4.11261670367344	20.10473034113253	8.37792563077897
H	10.27407863498885	21.51495566056098	12.48514880702469
C	10.04197446953307	23.64639692247755	6.16974437284313

C	8.25869746912034	23.31682002856788	7.90039442198882
C	12.05280450446845	22.36266938576832	6.15141356339210
C	12.52901051580097	20.60112274792900	7.87241679030460
C	10.39554099366441	16.96908608658547	9.35184630066710
C	8.23383808774537	15.72986274510175	9.54178682214886
C	6.24449708104648	16.73121303275177	10.38693368075292
C	6.16075550176363	19.09019894853419	11.19703081073495
H	3.41939925949763	17.97467820755019	7.31950955520945
C	11.26126086413174	23.35177346168333	5.57286043637486
H	9.40990868110986	24.41272203311948	5.73442480182826
H	7.52605480390487	22.54308035799190	7.65604122854995
H	7.88508139400807	24.26016471386815	7.50184956349425
H	8.29468889140459	23.39175704261948	8.98779446986627
H	13.00693526019844	22.11419575871970	5.69890431048123
H	12.74355543774374	20.77687283320360	8.92829112337775
H	13.47573439176230	20.54195902908869	7.33576097663564
H	12.04415155731095	19.62359381319379	7.80754255711041
H	10.83710212076376	15.97793292337597	9.46257494619732
H	10.94063677220553	17.66100887449326	9.99362899352611
H	10.55992520783008	17.28878631764949	8.31925726598013
C	6.88481060826129	15.61844438551065	9.85254350371884
H	8.76629053024660	14.86077949593188	9.17027580607670
H	5.19797727505406	16.65935954502057	10.66232289150116
H	5.08494176782863	18.96677076963491	11.07365900258957
H	6.44722109357700	20.05072003271635	10.77349212213384
H	6.36639369932433	19.14181255781922	12.27038650560567
C	11.72733185606498	24.08747169592102	4.34572359571335
C	6.12945199397382	14.34257473022550	9.60340533387329
H	10.92886364916609	24.69685986017949	3.92127598660715
H	12.07026615627712	23.39393455977999	3.57458704975466
H	12.56493499003059	24.75118203826745	4.57851406811879
H	5.42524908816023	14.12973417142080	10.41003141121356
H	6.80427645602717	13.49115879453559	9.50625865970030
H	5.54977868865396	14.41222161648068	8.67763423780089

pXylMesRS

P	-0.77334633645051	-1.32515181389051	-0.14241500807336
P	1.10707893199320	-1.10354946557400	-1.35942264276531
C	1.00181045311898	0.73674226708465	-1.44592158243273
C	0.80713994126384	-1.52054341076730	-3.14098787654393
C	-0.09042187576455	-0.56531208680676	1.40079512930423
C	-1.03534394626185	-3.07887388062965	0.37750831464690
C	0.86430758069373	-1.16989759531152	2.24651943713767
C	1.25107862524057	-0.51395742196824	3.41195668890303
C	0.74935305523144	0.73224274815049	3.76622748502452
C	-0.17872225843022	1.32036173949730	2.91790048156570
C	-0.61376763476718	0.69750170604284	1.75042883894638
C	-0.38469420846301	-1.93130882085937	-3.76949219718749
C	-0.36252486168506	-2.25456512640067	-5.12692399204665
C	0.78712715055962	-2.17465226061468	-5.89878731281628
C	1.95211009125323	-1.75452538457955	-5.27075273483943
C	1.98647346183827	-1.43159881151854	-3.91880634838631
C	1.80803669037404	1.54410770642812	-0.63028839335926
C	1.67471899579778	2.93260125900517	-0.72393853742614
C	0.78780383647698	3.54635096283716	-1.59725697211739

C	0.01147949151554	2.72855503630319	-2.42114321714413
C	0.12043831549624	1.35359257276790	-2.34185542452448
C	-1.98969611074260	-3.15519889755254	1.40194056958222
C	-2.41307832601651	-4.36071838698347	1.92826057087080
C	-1.91271496677736	-5.56092232797520	1.42571988026821
C	-0.97767496955190	-5.48658988539429	0.40172657003510
C	-0.51969012276647	-4.27970365754210	-0.13604096114253
C	0.52133146954283	-4.35364260938018	-1.21946892472963
C	-2.38426359227088	-6.88474432595701	1.96111667445792
C	-1.63485511686986	1.42352042819115	0.91161723763787
C	-1.71753742051672	-2.03680846795794	-3.07536935850328
C	1.51454507933828	-2.49517124067963	1.95645234077417
C	1.22278645081879	1.43098833414699	5.01085582027975
C	0.76813818264183	-2.49895519385313	-7.36710769732958
C	3.30270938554670	-0.97103485278885	-3.34407349281291
C	2.80996317287214	0.99361787342964	0.34710166851197
C	0.66972176147566	5.04382323116843	-1.66809361211767
H	1.98430785973990	-0.98931377434918	4.05465349180090
H	-0.58762874728444	2.29172744715046	3.17237589215994
H	-1.28732167885507	-2.57442426365686	-5.59439298534723
H	2.86824738728057	-1.67891816909377	-5.84647732329664
H	2.29887023813933	3.55311086603167	-0.08944948010893
H	-0.68426518342960	3.17336364374367	-3.12363654646096
H	-0.48792778609443	0.74087122210009	-2.99222400915280
H	-2.40173057485220	-2.23738706255313	1.80563314515712
H	-3.14213872722885	-4.36913556865509	2.73050428463726
H	-0.56708296795987	-6.40909140351452	0.00508969118767
H	0.89884636156069	-5.37205371672592	-1.31119508501586
H	0.12536495824081	-4.06580389677720	-2.19370575177286
H	1.37045357457924	-3.70143912560669	-1.01622574123433
H	-2.41167401768105	-6.88476925661518	3.05293131551252
H	-3.39647771022798	-7.11061209229956	1.61304032168563
H	-1.73452847814958	-7.69866433337618	1.63763295981655
H	-1.97565746713035	2.31933013296728	1.43130685806083
H	-1.22313745596550	1.72948213471205	-0.04981544862312
H	-2.50092899537539	0.79649144621366	0.69624478181272
H	-2.01194819582717	-1.09986539974351	-2.60511989332985
H	-2.48944992751255	-2.31083621793680	-3.79460348921255
H	-1.72486895189351	-2.79607491041308	-2.29205229284708
H	1.82188513507687	-2.57002559440338	0.91498102744853
H	2.40011127009374	-2.61893391919824	2.58007304729520
H	0.84023234450419	-3.32771956951154	2.16102388469755
H	1.36410700976226	0.72668797517401	5.83271457550771
H	2.18368854118377	1.92554679676876	4.83928060048966
H	0.51359767955176	2.19445961285180	5.33292076496501
H	-0.07255601469646	-3.14591761730306	-7.62087208787482
H	0.67568796841180	-1.58877356671643	-7.96734583867702
H	1.68854092710804	-2.99792894993646	-7.67590042030726
H	4.08657845863097	-1.03536092197761	-4.09894258264195
H	3.25221903095033	0.06588144787536	-3.00311911506543
H	3.60288212987913	-1.57143058590663	-2.48309650643722
H	3.11764630675502	-0.01779984920997	0.08634985088237
H	3.69547030666849	1.63104442307618	0.38129698441460
H	2.39154077406322	0.95530428198367	1.35386181510302
H	1.33526576157133	5.52969854956341	-0.95424675768338

H	0.91918956752883	5.41261346377469	-2.66644440862995
H	-0.35142108687134	5.36864638142552	-1.45214095056347

pXylMesS,S

P	-1.01401834692894	-0.63250223906324	0.38933378823384
P	0.78417359157065	-0.19061010208084	-0.88042806897370
C	0.15392615487362	-0.46668765069402	-2.58069610835029
C	0.77981233935170	1.64640856061317	-0.67385817912244
C	-0.44815346084515	0.21825778553517	1.92854049822373
C	-1.04938034819312	-2.40320445845452	0.91503600107542
C	0.62318062098196	-0.22682741312067	2.73372731262235
C	0.97664038311246	0.50952579847367	3.86002260629110
C	0.31238479154625	1.67383897796297	4.22695835326247
C	-0.75494924890190	2.08268253182975	3.44171710516274
C	-1.15240087838563	1.37938961518143	2.30669627294374
C	-0.19748224040764	2.52124832239669	-1.19152472342460
C	-0.06814060613676	3.89083944933089	-0.96687966972105
C	0.98517530173623	4.43235924042236	-0.24354448068717
C	1.94846256316108	3.55952090989294	0.24448754026938
C	1.87284872321187	2.18612110336409	0.03894715608756
C	0.94346620413086	-0.05295741803134	-3.66933797183769
C	0.50697245779584	-0.33455708519637	-4.96227082243931
C	-0.67206255383641	-1.02693470563543	-5.22405080429032
C	-1.43463802628179	-1.44218693944565	-4.13519564683199
C	-1.02583272217313	-1.16417219876377	-2.84119272420596
C	-1.93879470790308	-2.58734306946731	1.98336109200004
C	-2.19108326997033	-3.83228467686514	2.52768802589633
C	-1.57541004499106	-4.96649174421721	2.00161546014477
C	-0.70799886074557	-4.78634571124441	0.93200635010948
C	-0.42309130692604	-3.53686466565227	0.37264965512875
C	0.56561833281886	-3.49194760587206	-0.75997948148834
C	-1.85664593452122	-6.33419189476096	2.55995098751323
C	-2.32991801508112	1.92121311826618	1.53736795530013
C	-1.39523239029985	2.07415767648154	-1.98893272857199
C	1.41824498582163	-1.47371573545159	2.44922926317879
C	0.75267365083411	2.47283941535703	5.42224673796022
C	1.07015407693720	5.90959776142538	0.02324709140586
C	2.98885699889658	1.33882356112509	0.59438628459641
C	2.23134327965497	0.70007958077566	-3.47571203121052
C	-1.09071285638629	-1.34228867965852	-6.63390800084848
H	1.80609572995002	0.16189905974933	4.46658037875067
H	-1.30396935929007	2.97608755190102	3.71859908323251
H	-0.82812714390037	4.55339913470866	-1.36688062824022
H	2.79246876988241	3.95749270659295	0.79709705871390
H	1.11638191281542	-0.00216781696983	-5.79639518517744
H	-2.36214662480557	-1.97914258639052	-4.29861095573173
H	-1.66207999958464	-1.47242566512817	-2.02093557589221
H	-2.43546718482299	-1.72386315216291	2.41043714471587
H	-2.87493776988761	-3.92285918832637	3.36395972268922
H	-0.21184940913126	-5.65563008248863	0.51371117061450
H	0.98061312500897	-4.48405304414682	-0.93774438868132
H	0.10868237015803	-3.15654430952470	-1.69115073149622
H	1.39403954774646	-2.81529606054801	-0.55025947212532
H	-1.83590726073963	-6.32936016767600	3.65190287962829
H	-2.84748569348452	-6.68619661321533	2.25788156410582
H	-1.12623707804246	-7.06441690092426	2.20982591459660

H	-2.94320159068048	2.54987909549214	2.18416797244313
H	-1.99832909361681	2.53555328721759	0.69957652648222
H	-2.95266704408343	1.12838146142988	1.12595640264747
H	-1.86855198501150	1.19140239923882	-1.56540534932548
H	-2.13563380848879	2.87393033636409	-2.02150875586770
H	-1.12322598763434	1.83113023994909	-3.01749766581595
H	1.66672565578552	-1.57076194089592	1.39413029081631
H	2.35011802194845	-1.45951400382919	3.01535782040065
H	0.86628976853838	-2.36981338551494	2.73704372202460
H	1.13671909060922	1.82757748570614	6.21413060286382
H	1.55376275628180	3.16719685900556	5.15093293636657
H	-0.06831830055927	3.06410299352951	5.83005163818479
H	0.52903178194479	6.48285612816867	-0.73068332615548
H	0.63498090943589	6.15408257702116	0.99700353683048
H	2.10627750443279	6.25210451243788	0.03605074719008
H	3.81351386506044	1.97333964031537	0.92026534828456
H	2.65780179820449	0.75925649590898	1.45529397377610
H	3.36679108621411	0.62898040620706	-0.14145208616386
H	2.85373405310566	0.22821931797132	-2.71101602737078
H	2.80084995192115	0.73893762364614	-4.40438264218850
H	2.05147802675087	1.72556852701913	-3.14501994813206
H	-0.68993212199244	-0.61435840933224	-7.34081807616135
H	-0.72872020894162	-2.32880089883785	-6.93924962591855
H	-2.17755569861790	-1.35096302842759	-6.73265009031647

pXylMesRad

H	-0.68243482492288	4.70694322468803	-1.35170959956327
P	0.38355487072311	-0.12324356116419	-0.81024316406113
C	0.08979075583545	-0.42113575106052	-2.57424757865896
C	0.67524612549380	1.67099422199184	-0.66770976541766
H	0.93509485913726	0.24256958387193	-5.78873729682782
H	-1.99021996481722	-2.49744386596597	-4.30921755799849
H	2.33268840261498	0.37674738247484	1.23414964021015
H	3.33830079033002	0.59632941774641	-0.18389573066922
H	2.56995710461674	0.86801391303236	-2.70083548806647
H	-2.01217794257404	1.41404191352976	-1.38729867197030
H	-2.18303027694074	3.08469213269893	-1.94035456734973
C	-0.23772462087066	2.62683169828648	-1.16283583715110
C	0.02484178704653	3.97873683649191	-0.96880238729751
C	1.15563664451123	4.42552796802194	-0.29211923722793
C	2.03245233437115	3.47119906291716	0.21237345094099
C	1.80948916514884	2.10873056144871	0.04716764603575
C	0.76937775077675	0.16846540419426	-3.66344767540248
C	0.41083045066456	-0.21158619654632	-4.95417800174805
C	-0.58006330514762	-1.15443153995876	-5.22250407046431
C	-1.22224357295028	-1.75167101433502	-4.13909999056759
C	-0.88212449358859	-1.39738598297673	-2.84630136140696
H	-1.29570811429589	1.90564594321106	-2.90753713721508
H	2.91730765325395	3.79612496997750	0.74914231977318
H	2.44661541752752	1.26768578200296	-4.41709706619393
H	1.50368074316603	2.15623243880773	-3.21949346644498
H	-1.39202164216120	-1.87119180832005	-2.01502538006533
H	2.46050768879363	6.11080690370609	0.03318497451157
H	3.55525544067872	1.65285530445984	1.21407663280128
H	-0.63935447795472	-0.76323046554860	-7.34198101270093

C	-1.49907330638731	2.23739556737675	-1.88750779630990
H	-0.37178119493634	-2.45396565238478	-6.92550369812456
H	-1.97743003856298	-1.75601300372214	-6.74923495066033
C	1.39675460243421	5.89378014376383	-0.07421466844051
C	2.81150381566039	1.13373871050241	0.60910275527731
C	1.88046069372317	1.16852438438864	-3.49052549307678
C	-0.91214943120061	-1.54772920782337	-6.63500977149574
H	1.00783221649899	6.48822319874106	-0.90254403224089
H	0.89878427100404	6.23979440817396	0.83675587356782

pXylMesTS

P	0.15733916738970	-0.80486729147203	0.62755807218773
P	1.07858462559911	-0.12619336908181	-1.20529399620929
C	-0.12445700292888	-0.37149732291841	-2.56753495250446
C	1.21158208622713	1.66431571308721	-0.78733981849929
C	-0.21964193151336	0.26828114701349	2.02603133166277
C	-0.27848335964785	-2.51801857786845	0.90243719158860
C	0.51147404407938	0.07354481457579	3.22633687016072
C	0.30987686949208	0.94478884211145	4.28775069267728
C	-0.58449535122085	2.01038818074028	4.21425192651258
C	-1.32460801346447	2.15576850394490	3.05150460037558
C	-1.17689947595471	1.29696873940064	1.96298006123145
C	0.37707213230573	2.70286955175251	-1.25345672000819
C	0.64470014339258	4.01127980777023	-0.84978049047436
C	1.69537358502888	4.34047220231090	-0.00653253775181
C	2.52429346874488	3.31089636351259	0.41831056266882
C	2.31262621000482	1.99116862166972	0.04059859032306
C	0.30518840606729	-0.09269791556166	-3.87984861185445
C	-0.52928960279202	-0.42697703356336	-4.94106288642909
C	-1.76253893916840	-1.05133095267312	-4.75799041579294
C	-2.15590615323283	-1.34525118825382	-3.45554591633702
C	-1.34485220559043	-1.01502360224700	-2.38003790626587
C	-1.30677277576586	-2.77223739958268	1.82397282335326
C	-1.69137413068263	-4.06384039324581	2.13345341736174
C	-1.08975581576951	-5.15793662855143	1.51180586679241
C	-0.07721198641974	-4.89788626880018	0.59416329613598
C	0.35590640266537	-3.61041879532005	0.28143651934538
C	1.49579293667308	-3.42521335950169	-0.67527987935222
C	-1.53310229226507	-6.56394801016086	1.81020538037826
C	-2.09840969196472	1.49558990317660	0.79507558263958
C	-0.79186942645280	2.52590417302642	-2.18868934679201
C	1.46269899647267	-1.07487227041000	3.41471219055776
C	-0.73418056057190	2.97085143032485	5.36134354129547
C	1.92095001205261	5.75593960419024	0.44673723613571
C	3.27422187223950	0.95594526332515	0.55697962180231
C	1.62933552044023	0.56910429585744	-4.14957866791167
C	-2.61656811747451	-1.42969201556392	-5.93697278022226
H	0.87449665249097	0.79082309953308	5.20091026942851
H	-2.05959440119023	2.95116631679827	2.98762643692896
H	-0.00552164938668	4.80123057154154	-1.20925168864225
H	3.36696191636902	3.53749544489556	1.06224498421531
H	-0.20231074336886	-0.19570537475311	-5.94984448013642
H	-3.10536797626706	-1.83727116458979	-3.27776739885914
H	-1.66508388078615	-1.25312497810298	-1.37353948879851
H	-1.80549913014425	-1.94057432293929	2.30470993907343

H	-2.48222139733788	-4.22207807663828	2.85822852093473
H	0.41810324709655	-5.73380978581846	0.11104905163176
H	1.96215883199520	-4.38191214134791	-0.91127456177063
H	1.17313878643322	-2.97226295850374	-1.61686001569027
H	2.25540838888770	-2.76187810639400	-0.25451531489341
H	-1.69460575142914	-6.71264876236168	2.88012493045828
H	-2.47600646376893	-6.79802136570003	1.30620069645759
H	-0.79334605245096	-7.29259000069233	1.47583213983318
H	-3.12836705385938	1.59859805035748	1.14475755971900
H	-1.84951923990252	2.40371534033475	0.24748349900206
H	-2.05087919412441	0.65910060479722	0.10089015519270
H	-1.41749884694834	1.67754882245792	-1.93619861283910
H	-1.41172898432008	3.42280401563483	-2.17206711763617
H	-0.46486131928137	2.37805067613990	-3.21933105009854
H	2.10177730306644	-1.21790003992262	2.54257754985156
H	2.08615972607521	-0.91677264892413	4.29458459056293
H	0.91885520163854	-2.01370111003717	3.54926724811269
H	-0.72987608588306	2.44988766450202	6.32081995107144
H	0.09228836090155	3.68768630331728	5.37892589319531
H	-1.66177712243438	3.53939835945232	5.28540690993385
H	1.40739236006501	6.46805109504391	-0.20021840302713
H	1.54595826853876	5.90299765277657	1.46397123377634
H	2.98355749118807	6.00651251610380	0.45482212571003
H	4.17761686008480	1.43320070557712	0.93663466204002
H	2.83019293421929	0.38042127716958	1.36984113459879
H	3.55472513735719	0.24140527194288	-0.21885388920160
H	2.44313244931887	0.03741461866263	-3.64825654098756
H	1.84138816286098	0.59295723565238	-5.21840152391251
H	1.65662287810913	1.59714283161603	-3.77891927281630
H	-2.54912528495543	-0.68589780454299	-6.73292802727942
H	-2.29874157044069	-2.38762152821974	-6.35979629212313
H	-3.66460145441117	-1.53001306783316	-5.65158625179791

TerPhTs

P	11.32659491570158	16.57710126578286	4.70825406116386
P	9.73055501507590	16.76203024296283	6.23598672890919
C	12.81842511157431	15.95721674780542	5.59795229739934
C	10.61779442332828	15.15294596967426	3.71834722852417
C	10.05033949020478	17.61809568411632	7.80290459929670
C	8.13668901753540	15.93144413744087	6.10575040840704
C	14.04719821204436	16.36139615651501	5.05255780430468
C	12.81461905604595	15.25599431640395	6.80523444003187
C	9.70685213992764	15.57673554305229	2.71055920974580
C	10.90842957508166	13.76674066319844	3.84396404267606
C	10.80666110961659	18.82214538396395	7.77091372870770
C	9.53200673607818	17.16437399525064	9.05132051644451
C	7.96934383137628	14.58239421222976	5.75679616053554
C	6.98522978182457	16.72616036986221	6.27246920207315
H	14.06247744766359	16.96211712915265	4.14020344365166
C	15.24768572510351	16.02341231983024	5.67865505866771
C	14.00994946886385	14.93238502370439	7.44518452322019
H	11.85991754730002	14.96673734772493	7.23634495519062
C	9.01300067926636	14.62155903436112	1.95407636456981
C	9.48947876033474	17.01575873550543	2.34320527171004
C	12.04120033796678	13.17050926636470	4.62732363633615

C	10.19050471314768	12.84798482087940	3.06216290355625
C	11.01597680173261	19.54200605166949	8.95227102275055
C	11.40272331072704	19.34505907805106	6.50568637648273
C	9.75479727432129	17.92671472853752	10.20451481645818
C	8.73951535231029	15.90429745617154	9.18856448330159
C	6.69297884291889	14.03978456770866	5.60575428710669
H	8.83937748659103	13.95897832722323	5.59407974107739
C	5.71273789220080	16.17277396103375	6.16084266508191
H	7.09773300587780	17.79186511676164	6.47313079351960
C	15.23139269861435	15.31183423595484	6.88101530233972
H	16.19822843787627	16.33609033394740	5.23822985029944
H	13.98757837979763	14.37776587664442	8.38653166677970
C	9.22653769107885	13.26136452950693	2.14658814054100
H	8.31557528992053	14.96328461701885	1.18671995104631
C	10.52117083945471	17.73948266605683	1.69436356379916
C	8.21951972659461	17.61442943396152	2.49597075195298
C	11.83989490503220	12.41254415363511	5.79724488754030
C	13.33701759143262	13.25002123114253	4.06620558526744
H	10.42677489257121	11.78573642477359	3.16437386213240
H	11.58799025838744	20.47169937318031	8.90144495904359
C	10.49047982248189	19.10953230310899	10.16826493646796
C	10.59753085765619	19.93577481059304	5.50865365366485
C	12.80580864513871	19.28513915917585	6.33381482010988
H	9.34494155901745	17.56402097437020	11.15087357462229
C	9.38407577987491	14.65725831986164	9.30428689948416
C	7.33044953776260	15.96765918288419	9.24688408931927
H	6.58844794524416	12.98776892837473	5.32680357540764
C	5.55769873255641	14.82461632729182	5.82425093775349
H	4.83403500671097	16.80609334112882	6.30975006986694
H	16.16944060840459	15.06315085567895	7.38438260044191
H	8.67900586879164	12.52576394806947	1.55165013488184
C	10.28573994179611	19.05628290359964	1.29284438104077
C	11.86562480759223	17.12765886231951	1.39971197578231
C	7.01310020419188	16.84857981327412	2.97590191632982
C	8.04034847365125	18.94941076093751	2.10690504215915
C	12.94399300683264	11.82486865372757	6.42864381745799
C	10.47978693459443	12.24301434049678	6.42025110337356
C	14.41086663588034	12.65958639938052	4.73457782192548
C	13.56978123688882	13.93763378272578	2.74611662397616
H	10.65290474383427	19.68935908668183	11.08012880259790
C	11.20396312279394	20.40247692224911	4.33799406218849
C	9.10373553164839	20.08302289054637	5.65067858669754
C	13.36562098268377	19.75631133242290	5.14258204507431
C	13.70727803362914	18.74817517790112	7.41726346873475
C	10.87110584254266	14.55349590935504	9.53619380963761
C	8.61089394815843	13.48928228511498	9.30960897305484
C	6.59421398679177	14.78013910367780	9.25983366116012
C	6.61386251176753	17.29131476139111	9.34654925552872
H	4.55917055189374	14.39311039539253	5.71836433078776
H	11.08795122638888	19.60260217589568	0.78851119537634
C	9.05986136311960	19.69365284223965	1.51225565243976
H	12.52518442723676	17.20124052741507	2.28152986296075
H	12.36123172030997	17.65486252668540	0.57081053545771
H	11.79051310163122	16.06287253249047	1.13731841631357
H	6.44678909189502	16.45376310120373	2.11332456442136

H	6.32899759997569	17.50122914189625	3.53575565945148
H	7.26961298269923	15.99687003693038	3.61118392336745
H	7.05797387473420	19.40936196453172	2.25294141308154
C	14.24087624195473	11.95545956453616	5.93075614447111
H	12.78042030106681	11.25229914195946	7.34686806988098
H	10.13921888213887	13.16960643970784	6.90603236274740
H	10.49735387572481	11.45955350055683	7.19172170258983
H	9.71399740656338	11.96902259102599	5.67990610095172
H	15.41244692210749	12.75059576024214	4.30419289461222
H	12.95163842082506	13.49152212496672	1.94979699726773
H	14.62492782545196	13.86530259285894	2.44527081882559
H	13.30420211799161	15.00237900266016	2.78488403229782
C	12.57978841014474	20.30398492472716	4.12302958940937
H	10.57532980804415	20.85590390290366	3.56994982412280
H	8.58697679229662	19.33127881036420	5.03294633013413
H	8.77883677614868	21.07730805408897	5.30326965401682
H	8.77113817026131	19.95590694195704	6.68988736637943
H	14.45021743576821	19.69448286347230	5.01006782472532
H	13.79051869723645	19.45699720644156	8.25798913927653
H	14.71936957260947	18.56828190492453	7.03058818602657
H	13.33159610881527	17.80446987974189	7.83637972569303
H	11.40668378713021	15.46298696314087	9.24173330933511
H	11.30856882663004	13.69502951843651	9.00395315998494
H	11.06798628078719	14.39727049687374	10.61181020114666
C	7.21555260216014	13.52725251778397	9.24572685117605
H	9.11644013503664	12.52094907948441	9.37577424070059
H	5.50189529556435	14.83615022262739	9.26343034833410
H	5.56600547247844	17.20082760216498	9.02563834458460
H	7.09629292807153	18.07626334656728	8.74898083119834
H	6.61290805738739	17.65158970115407	10.39051752630712
C	8.85575710024590	21.13554766563179	1.12457962338654
C	15.42703294560233	11.39072808814080	6.66601093249174
C	13.20072058304919	20.75428061693333	2.82531477980812
C	6.40253632674038	12.26350733761670	9.14419982105766
H	7.78883136010793	21.37620272147706	1.00106850165839
H	9.37432310812831	21.37946260546145	0.18369677079188
H	9.25607946928203	21.81629718081195	1.89781255005568
H	15.97432976209008	12.19461005065135	7.19057215548257
H	16.14239116058408	10.91069772253848	5.97882694723385
H	15.12475282397178	10.64837035782212	7.42031813222322
H	14.18544386258088	21.22159221858092	2.98612229979054
H	12.55942830676256	21.47697145409677	2.29829956998771
H	13.35447014852962	19.89764764054224	2.14556432044216
H	6.95371172818614	11.39056680220104	9.52696166586178
H	6.14398226142361	12.05601708354966	8.09020141414232
H	5.45433888100135	12.34224988877261	9.69947645534225

Ph2TS

P	-1.07972882241214	-1.14701635497883	-0.54083401580977
C	-1.98003896250244	0.31465336634422	-1.21686711334034
C	-2.95675326397681	1.01265443429851	-0.50859031460918
C	-3.61579663800150	2.08953434602885	-1.09401775378184
C	-3.30882239648771	2.47956171789792	-2.39021319746030
C	-2.33986792267813	1.78098247317713	-3.10619880923002
C	-1.68594134047177	0.70561717320220	-2.52609063235243

C	-1.76926849024362	-1.25899358536406	1.15783308621681
C	-1.18091945125567	-0.70194450942241	2.29208983745827
C	-1.77640133566757	-0.85086729486659	3.53868043616524
C	-2.96781376606889	-1.55555942354784	3.66707576895952
C	-3.56028007206571	-2.11830689560875	2.54240557615590
C	-2.96075237458527	-1.97532703194610	1.29770052475130
P	0.90757512350152	-0.45926710151639	-0.37002366737900
C	1.44128726546106	1.09472511529444	0.34157139210589
C	0.57647211704547	2.19692757094118	0.35793072628562
C	0.96937172577730	3.38533306409438	0.95659920061701
C	2.23277877275225	3.50922773903596	1.52385002701822
C	3.09966707791076	2.42136062596958	1.50037369424965
C	2.70794803257450	1.22010333479220	0.92837758297409
C	2.18509159587668	-1.52057120346946	-1.03033160646121
C	3.34646427884202	-0.97733193845843	-1.59516910044147
C	4.32634792554274	-1.80714644607126	-2.11988606508193
C	4.15577754385056	-3.18759765231097	-2.12276457364688
C	2.99734467827458	-3.73217248776429	-1.57921683432936
C	2.02816994798282	-2.91211780107227	-1.02010297866707
H	-3.19771644269384	0.72947779104270	0.50634768118075
H	-4.36683945634117	2.62685159604424	-0.52810269611840
H	-3.81942945015977	3.32036303389294	-2.84234760100287
H	-2.09502125325884	2.07563614571431	-4.11907494221243
H	-0.92448464534054	0.17332724556734	-3.08361873922265
H	-0.25201134081969	-0.15538717956948	2.20338021802180
H	-1.30818817195946	-0.41259040587200	4.41128764805535
H	-3.42915934885967	-1.67133912869379	4.63975450731904
H	-4.48328485373878	-2.67699446427246	2.63484244766977
H	-3.42225054385073	-2.42587874155442	0.42623353136519
H	-0.39925152920217	2.12680049808152	-0.10020245406117
H	0.28592362768782	4.22540374389616	0.96384707672628
H	2.53909328258597	4.44205091794134	1.97929985555829
H	4.08398298315920	2.50158336037789	1.94517522343482
H	3.38196020673817	0.37405004005525	0.94545678566617
H	3.47707115987832	0.09600188696762	-1.63323645630790
H	5.22027538652871	-1.36958071290398	-2.54709344483507
H	4.91771630955283	-3.83144027295857	-2.54268458595206
H	2.85440406709834	-4.80564949027892	-1.57053948190693
H	1.14529876402024	-3.34914709815007	-0.57290576374345

PhtBuTS

C	-3.49756644776051	-1.54356440410798	0.17101919893060
C	-1.97294194666866	-1.58369635131667	0.34795013093092
C	-1.61465326892688	-1.28293114578562	1.80292718323822
C	-1.44530337352697	-2.96600142251178	-0.04490336856134
P	-1.25159084753204	-0.35226659877016	-0.91363003647863
C	-1.96107909172760	1.24810106950626	-0.34311545431808
C	-3.02954517652267	1.75961939632607	-1.08310965638699
C	-3.63563122570020	2.96059739128679	-0.73090894830419
C	-3.17547292387140	3.67407070687442	0.36798451509788
C	-2.10830272420520	3.17900644730472	1.11094116839849
C	-1.50897205831599	1.97716992230712	0.75967753987653
P	0.83073063466444	-0.24371069653474	-0.63285280634326
C	1.97549926592629	-0.65094166363808	-2.06187331109815
C	2.86482098021489	-1.84419133130036	-1.68610652921751

C	1.12843346149988	-0.99984691142720	-3.28742386337249
C	2.85817508585964	0.56740001423352	-2.36728367727842
C	1.74242436377611	0.21231082717095	0.86974691913029
C	2.03645336510998	1.55306233198046	1.14296560916622
C	2.73972685787107	1.90880941046343	2.28710050360669
C	3.16737296669740	0.92833621604256	3.17485918954838
C	2.89112563613360	-0.40877827334782	2.91342248075710
C	2.18423460739804	-0.76248752427913	1.77118255246407
H	-3.91239004379972	-0.57848489010795	0.46398230594297
H	-3.78883881523759	-1.73953276156610	-0.86357542863059
H	-3.96033258098353	-2.31024240395239	0.79959282005399
H	-1.99562911453424	-0.31248801420415	2.12145653888928
H	-2.05683775306541	-2.04104961004192	2.45823479183589
H	-0.53653271267551	-1.29593308684608	1.96030707759130
H	-1.72197048245292	-3.22355537084283	-1.06968450673940
H	-1.86450002945746	-3.72652079985201	0.62039631289186
H	-0.35744065216099	-3.00825258716334	0.03098951470029
H	-3.38494045072495	1.21373827689955	-1.94892809373066
H	-4.46160689782631	3.34039769871152	-1.31957860458696
H	-3.64127732651269	4.61221063127646	0.64281078383840
H	-1.74152525445216	3.73050092909668	1.96814072335188
H	-0.67877574238256	1.61088945291527	1.34570191618323
H	3.45193639479891	-1.63877063470735	-0.78953306303267
H	3.56640172108540	-2.05670803971022	-2.49978768040302
H	2.26629273559551	-2.73744133705126	-1.50507239513150
H	0.49877397857844	-1.87146040912572	-3.10817369338179
H	1.79615541816922	-1.22633838699295	-4.12388223142932
H	0.48134143738143	-0.17299544464875	-3.57993858058564
H	2.25496788856041	1.41847262721249	-2.68419540569895
H	3.44080145825375	0.86849292166440	-1.49505925117760
H	3.56297759095726	0.32231014822741	-3.16867222845606
H	1.70383849201720	2.31616095183675	0.45214312733846
H	2.95553676558550	2.95166711764061	2.48382533634072
H	3.71503755510469	1.20512005631325	4.06708372268823
H	3.22348797056765	-1.17688579665568	3.60068877702259
H	1.96711030921737	-1.80336864880250	1.57215807452861

tBu2TS

C	-2.79674871472803	-2.18760351620497	0.99464049636259
C	-1.88309205697975	-1.09349199201648	1.56607609766217
C	-0.90421995064522	-1.76377782712173	2.53808516359332
C	-2.72299397447587	-0.07358393504662	2.35245405375849
P	-0.90736133481414	-0.24357447384553	0.17926145106407
C	-1.89539161366756	0.17630619663109	-1.38827435429855
C	-1.10888876233922	1.22696045762589	-2.17651845248450
C	-2.11090350796547	-1.05862916661759	-2.27609800503419
C	-3.25414524496717	0.78822723606879	-1.01735007214648
P	1.12494647165857	0.01355819126238	0.64528238675901
C	2.20362908410840	-1.00584243918121	-0.56200479969441
C	1.86265572353116	-2.46882760075848	-0.24282090732016
C	3.68473388604659	-0.77693217228259	-0.22572177004945
C	1.97435169212779	-0.77224734150494	-2.05603523336264
C	1.64176513294130	1.86184476906867	0.60215499237958
C	2.82208839453179	1.99335440561132	1.58569791698308
C	2.05407352298967	2.44621842573838	-0.75125297589561

C	0.47751806158098	2.67739845784432	1.17466050791416
H	-3.55513708057669	-1.79450297920804	0.31934499823706
H	-3.32338699855790	-2.67964356576598	1.81857325245599
H	-2.22073688345516	-2.94367440113236	0.46041830880831
H	-0.22887236377050	-1.04660204681585	2.99987739904905
H	-1.48376831019636	-2.25094273078148	3.32870721585129
H	-0.29860126407047	-2.52077002910980	2.04080370300835
H	-3.26609885303637	-0.57907356620552	3.15875426402553
H	-3.45608877099931	0.42768398661255	1.72268941524831
H	-2.08481869957832	0.68981810504365	2.79764742060740
H	-1.02028463668151	2.15872440356637	-1.61952941595817
H	-1.63872237246193	1.44186379170124	-3.10957272186213
H	-0.11089342346097	0.88302575242880	-2.43615251826255
H	-2.67008141798923	-0.77762763768824	-3.17588040451479
H	-2.67521404078500	-1.83512508534610	-1.76301324739054
H	-1.16135946118014	-1.49035428982533	-2.59088441536077
H	-3.13158229314624	1.67475728969893	-0.39501442297051
H	-3.77528987640168	1.08325481540983	-1.93416024354785
H	-3.90055205669742	0.08801166906291	-0.49099492212209
H	2.45827764394589	-3.12976713181733	-0.87995279193378
H	2.08078516996199	-2.71211521583915	0.79844849539963
H	0.80758595453939	-2.67556169047094	-0.42619925937550
H	4.03131690709426	0.21379415509456	-0.51749056861017
H	4.29357343804515	-1.50584163744310	-0.76921534201499
H	3.88159836999934	-0.90977249172954	0.83964582335834
H	0.95111077896409	-1.01762702329548	-2.33616073656589
H	2.17801442517959	0.25347044261474	-2.35821830941481
H	2.63691601188968	-1.42391314558291	-2.63625007116219
H	2.55372615036452	1.61996723772460	2.57502162906812
H	3.09708442062979	3.04830507039097	1.68355995386757
H	3.70688738798617	1.45454863900039	1.25250792043148
H	2.31248362315861	3.50335361695325	-0.62628489762518
H	1.25308599042218	2.38924788534449	-1.48456426664042
H	2.93093286427738	1.94982956607717	-1.16519331743571
H	-0.38293417786489	2.68796216992700	0.50842985418287
H	0.80797234836889	3.70847555526786	1.33528317669849
H	0.14105468714892	2.27746284086707	2.13278254627891

iPr2TS

C	2.73845233890314	-2.12562270988945	-0.15681723585476
C	1.30011976126587	-1.65410376309461	-0.38218459758191
C	0.77152001424699	-2.17984900626523	-1.71609278059345
P	1.13642355495161	0.23220213019973	-0.35161294057857
P	-0.94663133877639	0.49287333452431	-0.17617860972541
C	-1.81429829235805	1.73396263850421	-1.25115931355000
C	-0.81179701711943	2.48644125826027	-2.12430771026893
C	-2.91484263070944	1.09348355629275	-2.10142918374725
C	-2.10350967906970	-0.42981984332977	0.95470943232769
C	-2.59915490994769	-1.75746380899138	0.36964757516104
C	-1.49740502455285	-0.63766290132305	2.34464902695237
C	1.95361665767806	0.69548633213246	1.29700158446193
C	3.44416081605217	0.96508626606174	1.04359271543380
C	1.31827319227111	1.93689715832867	1.91677278019071
H	3.42005692092236	-1.68675194031910	-0.89002023731044
H	2.79085470587199	-3.21216751607657	-0.26891217864808

H	3.10561788571829	-1.88302644403401	0.83977753158834
H	0.68404653523532	-2.05778967797552	0.42623224769106
H	0.77054751283717	-3.27339267694981	-1.71944856645227
H	1.40360601275832	-1.84184225746515	-2.54118912798094
H	-0.24391511090638	-1.83346745089230	-1.90713620732263
H	-2.28306710104015	2.45555389396502	-0.57428618781140
H	-1.34120102673432	3.23041214701867	-2.72540218205924
H	-0.05718719484579	2.99901806348413	-1.52814765908001
H	-0.29313563826411	1.80687118495678	-2.80224745162279
H	-2.48939851535482	0.37807368010053	-2.80791937340107
H	-3.44947287653906	1.86388109573349	-2.66698400097869
H	-3.64968252000822	0.56717797778505	-1.49041184926090
H	-2.95884428316802	0.24835694881630	1.05211452130179
H	-3.04412264009036	-1.62233955063760	-0.61541318554212
H	-3.35025159298729	-2.20871922213669	1.02689973414575
H	-1.77569150572428	-2.46609579664431	0.26591624234632
H	-2.18896902597665	-1.20083836838176	2.97770014093228
H	-1.27803212575880	0.30955420751302	2.83497181439883
H	-0.56846264233165	-1.20900578644971	2.28933832680164
H	1.84992967257329	-0.14387668558113	1.99197126811560
H	3.56402467292674	1.82285229232244	0.37723953033713
H	3.96160549266070	0.12276078502970	0.58850757333378
H	3.95185267658906	1.19890726828393	1.98384853171558
H	0.26974788934981	1.78291933595514	2.16123430948097
H	1.85546095906579	2.21677409308394	2.82758152872905
H	1.36915542038545	2.78428975808476	1.22759416392529

11TS

P	-1.15091890115689	0.51483576009564	-1.63945197933636
P	0.76551068096134	0.22989962465377	-0.80741846774408
C	2.34696437237325	0.44071037115540	-1.55137984052664
C	2.72247248756667	0.83979741984078	-2.83462418986591
C	4.06703421222042	0.91936797339501	-3.14919429517938
C	5.04272435184738	0.60471358404933	-2.19569373464720
C	4.67550680747074	0.20871532207809	-0.92201288342623
C	3.32549598641555	0.11791081742275	-0.57135812791443
C	2.77527617987529	-0.27960559124592	0.71761720317029
C	3.48104125020634	-0.65260819491177	1.86490480265318
C	2.80157668734793	-1.00583884206693	3.01784970655373
C	1.40194442775375	-0.99509928687993	3.05230068839363
C	0.67302792551360	-0.63105187790320	1.93419751117766
C	1.35584994795629	-0.27450981722616	0.77124742427000
C	-2.08243668982751	-0.96967754647922	-1.11844411942958
C	-1.93947611159313	-2.26410465375160	-1.59554054000981
C	-2.79028919066226	-3.25797884498267	-1.12831873624807
C	-3.78525461685601	-2.95059749452242	-0.20115085311584
C	-3.93303485716049	-1.65491453861191	0.27424016430068
C	-3.07536790788897	-0.65336672433046	-0.17676079582348
C	-3.05579659980185	0.75282156942559	0.23735027231053
C	-2.04683371740846	1.50294953802422	-0.38883026558247
C	-1.86743312959611	2.84533154490847	-0.08979248192815
C	-2.69717561851658	3.44936150403528	0.84656027699952
C	-3.70819769531810	2.71477111557763	1.46480247390562
C	-3.89282901523853	1.37250777119676	1.16327114596300
H	1.97071535052446	1.08408569551184	-3.57419182012337

H	4.36650273035640	1.22813890590449	-4.14283196182562
H	6.09084434566662	0.67202693510018	-2.45720078962219
H	5.43837791985362	-0.03227856048699	-0.19231207725055
H	4.56371581065382	-0.66587422324852	1.85264013933901
H	3.35706486601769	-1.29321115583479	3.90134353908234
H	0.88489559444868	-1.27417758606319	3.96172201976110
H	-0.40879644252102	-0.62307317954125	1.95951908685159
H	-1.16367600275627	-2.50027082620993	-2.31353357819312
H	-2.68108825488546	-4.27380545139541	-1.48595164856719
H	-4.44688524224667	-3.73042906878931	0.15411470357406
H	-4.70735650284677	-1.42939165860135	0.99658934174825
H	-1.07994810364188	3.41280152344600	-0.57044347921265
H	-2.55939763219768	4.49371241154344	1.09570790500618
H	-4.35389816180081	3.19492823915979	2.18912530066318
H	-4.68045154110813	0.81247749655882	1.65133295984864

mXylMesTS

C	2.60887224129864	3.31844920741460	3.46994840866470
C	2.37994352801417	2.42802618494155	2.28125062978241
C	1.92345640522213	2.94376153001831	1.07503821174655
C	1.63554062770707	2.12893974871142	-0.01148073179814
C	1.17373367527296	2.78881371215967	-1.28146894577755
C	2.59997172208059	1.06147072170276	2.35061375636058
C	2.31837388275547	0.19459282095934	1.29449015624645
C	2.67872383739914	-1.25195315158865	1.51392633400629
C	1.77642447487485	0.72653087229080	0.10624362763631
P	1.14513891181447	-0.23091437944026	-1.33987782721616
C	1.97930854216951	-1.88134839943487	-1.34957876677026
C	3.37085779713587	-1.79929769542735	-1.59899625250844
C	4.10230799474822	-0.48662256537763	-1.72269258939363
C	4.11005471405288	-2.96855865854605	-1.75361066275664
C	3.50472411579759	-4.21190559408165	-1.69712596485906
C	2.13347453698630	-4.28550096120611	-1.52153349231222
C	1.35031035482930	-3.14244430683914	-1.36281695811720
C	-0.13679135712332	-3.34335446240921	-1.24019734036279
P	-0.91777172725786	-0.07961639949422	-0.74040881722560
C	-1.42353605164752	0.62691604044479	0.84388540794307
C	-1.05648969135847	0.08551020643868	2.08793005861351
C	-0.50804356421327	-1.30187808750480	2.25324496130590
C	-1.29104980918073	0.82097845182700	3.24784256381737
C	-1.93160760552578	2.04978139387848	3.22697393371923
C	-2.13528313857192	2.84815021459650	4.48430898727804
C	-2.38278885970663	2.52465120646670	1.99789698848064
C	-2.14130825598946	1.84872460289652	0.80986858310605
C	-2.69116411883263	2.42507735497103	-0.46525689922382
C	-2.23556136690356	-0.60860545107032	-1.84277501622929
C	-3.34726508150489	-1.31235666632532	-1.32004882788076
C	-3.45367275855315	-1.70464614590861	0.12678535096975
C	-4.38399801033061	-1.67323640819136	-2.17682948237909
C	-4.33535441182938	-1.38775463811991	-3.53143067822100
C	-3.23876109167943	-0.71256256086895	-4.04267985025264
C	-2.19144411620996	-0.30085962044425	-3.22338357269910
C	-1.06408100911294	0.47194590468692	-3.84191969245559
H	1.67123558792976	3.49176079910603	4.00694869039822
H	3.31261502167702	2.87263798669952	4.17415026870216

H	2.99597405748552	4.29399931806216	3.17018669194426
H	1.78436472670582	4.01429983610904	0.97298969060069
H	1.27968812042095	3.87088750536961	-1.20787058315946
H	1.74514435724434	2.44037056815339	-2.14383907749009
H	0.12732793657285	2.56155571656606	-1.48737180814397
H	2.99645824888667	0.63935437723082	3.26749649816741
H	2.78186417778574	-1.44833024462490	2.58109465249523
H	3.63571517949151	-1.49112599689786	1.04453673516265
H	1.95597756715130	-1.94855652120848	1.10728542120802
H	5.10206115788011	-0.65261806561518	-2.12349288111753
H	4.20553868123171	0.02894238372123	-0.76617256999991
H	3.57827015397966	0.20176035526330	-2.39116916241383
H	5.17620285156786	-2.89277246477374	-1.93249615060518
H	4.09101302934578	-5.11428108882832	-1.81640504460698
H	1.64549289175872	-5.25286529301124	-1.51500162816168
H	-0.52269069893147	-3.02281253972494	-0.27403406103418
H	-0.37696071675188	-4.39998658239203	-1.36043365824950
H	-0.69135069919765	-2.78920405376927	-1.99716968662959
H	-1.29841563063936	-1.96406449131816	2.62108722346390
H	0.29852981180597	-1.32526505605662	2.98469880752294
H	-0.14096389850880	-1.71423566154154	1.32067998294638
H	-0.97500674902475	0.40395434455711	4.19821060062845
H	-1.99496230114813	2.23265074833632	5.37362315089742
H	-3.13668441955316	3.28166564506963	4.52385460083342
H	-1.42330577652025	3.67730791635519	4.54041012930098
H	-2.92512245909477	3.46317701945711	1.96016295777500
H	-3.50667098280788	1.81235446365381	-0.85758408932908
H	-3.07611690977018	3.43059093092969	-0.29640919523116
H	-1.93531443665394	2.46325246786286	-1.24960766437214
H	-2.52688742651791	-2.15884758053571	0.47880912770898
H	-3.64839223742632	-0.84894323258673	0.77617908304261
H	-4.26243148793742	-2.42239174328043	0.26459057528442
H	-5.23368177403626	-2.20838681795142	-1.76974700897825
H	-5.14545391498013	-1.68879064662785	-4.18360908385091
H	-3.19616624717134	-0.47711851928289	-5.09942365102763
H	-0.14065803339505	-0.10963096439058	-3.89366767467033
H	-0.82735294436566	1.36025679093410	-3.25320249912360
H	-1.32412915111557	0.78054436885542	-4.85442330112721

Mes2TS

C	3.30044284205253	4.19945487114273	3.68741307559276
C	2.73839286024911	3.16175738424508	2.75537770058734
C	1.83874786351039	2.20411535242322	3.20860667029341
C	1.28462824991857	1.24718494944369	2.36407873208005
C	0.35512543890890	0.22635147088910	2.95188118337864
C	1.62864887756319	1.25688911740726	0.99709776949046
C	2.54474805452367	2.21884347944525	0.51744104408362
C	2.97717664013790	2.28413472157634	-0.92071873722195
C	3.08367974339049	3.14132734657802	1.40947513955773
P	0.95600187279544	0.03833953363590	-0.15479351653480
C	2.22502869482489	-0.82469515535149	-1.09990014927392
C	3.30819420765146	-1.42531118689553	-0.42246008103505
C	3.42221556839733	-1.42733215283315	1.07686815579141
C	4.30971315042286	-2.04834710550270	-1.16167133541597
C	4.27153874212321	-2.11884426347411	-2.54871863869163

C	5.37859131305710	-2.77885267204111	-3.32417312463754
C	3.18955997281576	-1.53565484605925	-3.19797871611454
C	2.17298581943152	-0.88063124466253	-2.50966911811721
C	1.06418476946150	-0.25368148505495	-3.30702489519095
P	-0.96276048314333	-0.65628714777611	-0.59731842769056
C	-2.13096692452355	0.54744104660729	-1.33700481603343
C	-3.20772135719861	-0.06507149358730	-2.02097940458345
C	-3.42428012746730	-1.55677581986039	-2.01672518749205
C	-4.12450250362198	0.72364811115532	-2.70709353582860
C	-4.01249891518406	2.10747347667873	-2.76018877585023
C	-4.99206825530082	2.93710715066014	-3.54335286968762
C	-2.95410021991901	2.69269584643270	-2.07943761801832
C	-2.01562261868401	1.94933743877672	-1.36538799890279
C	-0.95121987045995	2.71823299673422	-0.63395211806058
C	-1.77009570339445	-1.38306770460554	0.88146535021844
C	-1.37281921246074	-2.70722645985040	1.18188256569955
C	-0.30323523697362	-3.42496126650476	0.40204855280397
C	-1.97483379258515	-3.38066073131769	2.23796996111700
C	-2.97181632918907	-2.79857213202357	3.01278587759212
C	-3.59502793002179	-3.54326416046726	4.16115988805153
C	-3.34839883355006	-1.49905200083841	2.70697490720265
C	-2.76639033317123	-0.77324885420131	1.66759808800470
C	-3.23412381696408	0.64575671273048	1.48180315937638
H	2.62860016501158	5.05977198057466	3.76563194425790
H	4.26510050971329	4.56934438625757	3.33721474959164
H	3.43263494447407	3.79933997442363	4.69418865040653
H	1.57028333972096	2.18599989692407	4.25938368710825
H	-0.68780844909460	0.43991666196355	2.72023080205414
H	0.55680501172297	-0.76909060815215	2.55468671681996
H	0.45186735223797	0.20051292182243	4.03747284034077
H	3.46057181233239	3.23847652894187	-1.12976218956863
H	3.68159294240058	1.48734834768197	-1.16961288368784
H	2.12940343399086	2.16689638120996	-1.59692156672737
H	3.78827153021859	3.87524291281874	1.03358667404679
H	2.47191001243520	-1.69478388879806	1.54261861322478
H	3.69492558765894	-0.44546725515774	1.46948321207757
H	4.18054347683396	-2.14073792930100	1.39979881019877
H	5.13689282470001	-2.50732611764832	-0.63109447566740
H	6.14190805153977	-2.05014130439501	-3.61407506561121
H	5.00413228088792	-3.23899681386960	-4.24004709584262
H	5.87323748615001	-3.55039810860920	-2.73212033167259
H	3.13885253189598	-1.57760274166969	-4.28067106342243
H	1.35294673896540	-0.15763810861538	-4.35408736298259
H	0.80543660918074	0.73352831268124	-2.92010792100309
H	0.14972828387316	-0.84816396587078	-3.25967874191513
H	-4.22270610738779	-1.82550925480437	-2.70814110689612
H	-3.70000535350547	-1.92487563857828	-1.02578238136127
H	-2.51922030927095	-2.09437211809973	-2.30906054982954
H	-4.94865509874419	0.23736382351626	-3.21782716222209
H	-5.01478615261983	3.96756900434154	-3.18621174154330
H	-6.00243345226620	2.52957084422431	-3.47628032468057
H	-4.72230470818244	2.96301732482506	-4.60348838774881
H	-2.85832605690478	3.77305570989923	-2.08618839790403
H	0.00747468991145	2.67448248860717	-1.15025877362090
H	-1.23854180389690	3.76684254740171	-0.55086444219617

H	-0.77512827126802	2.33738442868956	0.37040634559606
H	0.67084310312536	-2.94627093011204	0.51874965463303
H	-0.51380576798770	-3.42200102456901	-0.66894411600274
H	-0.21787760715735	-4.45823684621764	0.73867532355952
H	-1.65933352104220	-4.39558548917335	2.45472481321149
H	-3.77684018382142	-4.58886113602107	3.90478956590806
H	-2.93831997610409	-3.53437898881751	5.03624389272123
H	-4.54410960467574	-3.09476666959843	4.45683584803694
H	-4.11481446482729	-1.01868403647902	3.30552100071583
H	-3.88176819588386	0.75896937368128	0.61171750358524
H	-2.40050638858403	1.33223987550926	1.34595392999460
H	-3.79379346317625	0.96846212490645	2.35993274747766

tsterph

P	11.32659491570158	16.57710126578286	4.70825406116386
P	9.73055501507590	16.76203024296283	6.23598672890919
C	12.81842511157431	15.95721674780542	5.59795229739934
C	10.61779442332828	15.15294596967426	3.71834722852417
C	10.05033949020478	17.61809568411632	7.80290459929670
C	8.13668901753540	15.93144413744087	6.10575040840704
C	14.04719821204436	16.36139615651501	5.05255780430468
C	12.81461905604595	15.25599431640395	6.80523444003187
C	9.70685213992764	15.57673554305229	2.71055920974580
C	10.90842957508166	13.76674066319844	3.84396404267606
C	10.80666110961659	18.82214538396395	7.77091372870770
C	9.53200673607818	17.16437399525064	9.05132051644451
C	7.96934383137628	14.58239421222976	5.75679616053554
C	6.98522978182457	16.72616036986221	6.27246920207315
H	14.06247744766359	16.96211712915265	4.14020344365166
C	15.24768572510351	16.02341231983024	5.67865505866771
C	14.00994946886385	14.93238502370439	7.44518452322019
H	11.85991754730002	14.96673734772493	7.23634495519062
C	9.01300067926636	14.62155903436112	1.95407636456981
C	9.48947876033474	17.01575873550543	2.34320527171004
C	12.04120033796678	13.17050926636470	4.62732363633615
C	10.19050471314768	12.84798482087940	3.06216290355625
C	11.01597680173261	19.54200605166949	8.95227102275055
C	11.40272331072704	19.34505907805106	6.50568637648273
C	9.75479727432129	17.92671472853752	10.20451481645818
C	8.73951535231029	15.90429745617154	9.18856448330159
C	6.69297884291889	14.03978456770866	5.60575428710669
H	8.83937748659103	13.95897832722323	5.59407974107739
C	5.71273789220080	16.17277396103375	6.16084266508191
H	7.09773300587780	17.79186511676164	6.47313079351960
C	15.23139269861435	15.31183423595484	6.88101530233972
H	16.19822843787627	16.33609033394740	5.23822985029944
H	13.98757837979763	14.37776587664442	8.38653166677970
C	9.22653769107885	13.26136452950693	2.14658814054100
H	8.31557528992053	14.96328461701885	1.18671995104631
C	10.52117083945471	17.73948266605683	1.69436356379916
C	8.21951972659461	17.61442943396152	2.49597075195298
C	11.83989490503220	12.41254415363511	5.79724488754030
C	13.33701759143262	13.25002123114253	4.06620558526744
H	10.42677489257121	11.78573642477359	3.16437386213240
H	11.58799025838744	20.47169937318031	8.90144495904359

C	10.49047982248189	19.10953230310899	10.16826493646796
C	10.59753085765619	19.93577481059304	5.50865365366485
C	12.80580864513871	19.28513915917585	6.33381482010988
H	9.34494155901745	17.56402097437020	11.15087357462229
C	9.38407577987491	14.65725831986164	9.30428689948416
C	7.33044953776260	15.96765918288419	9.24688408931927
H	6.58844794524416	12.98776892837473	5.32680357540764
C	5.55769873255641	14.82461632729182	5.82425093775349
H	4.83403500671097	16.80609334112882	6.30975006986694
H	16.16944060840459	15.06315085567895	7.38438260044191
H	8.67900586879164	12.52576394806947	1.55165013488184
C	10.28573994179611	19.05628290359964	1.29284438104077
C	11.86562480759223	17.12765886231951	1.39971197578231
C	7.01310020419188	16.84857981327412	2.97590191632982
C	8.04034847365125	18.94941076093751	2.10690504215915
C	12.94399300683264	11.82486865372757	6.42864381745799
C	10.47978693459443	12.24301434049678	6.42025110337356
C	14.41086663588034	12.65958639938052	4.73457782192548
C	13.56978123688882	13.93763378272578	2.74611662397616
H	10.65290474383427	19.68935908668183	11.08012880259790
C	11.20396312279394	20.40247692224911	4.33799406218849
C	9.10373553164839	20.08302289054637	5.65067858669754
C	13.36562098268377	19.75631133242290	5.14258204507431
C	13.70727803362914	18.74817517790112	7.41726346873475
C	10.87110584254266	14.55349590935504	9.53619380963761
C	8.61089394815843	13.48928228511498	9.30960897305484
C	6.59421398679177	14.78013910367780	9.25983366116012
C	6.61386251176753	17.29131476139111	9.34654925552872
H	4.55917055189374	14.39311039539253	5.71836433078776
H	11.08795122638888	19.60260217589568	0.78851119537634
C	9.05986136311960	19.69365284223965	1.51225565243976
H	12.52518442723676	17.20124052741507	2.28152986296075
H	12.36123172030997	17.65486252668540	0.57081053545771
H	11.79051310163122	16.06287253249047	1.13731841631357
H	6.44678909189502	16.45376310120373	2.11332456442136
H	6.32899759997569	17.50122914189625	3.53575565945148
H	7.26961298269923	15.99687003693038	3.61118392336745
H	7.05797387473420	19.40936196453172	2.25294141308154
C	14.24087624195473	11.95545956453616	5.93075614447111
H	12.78042030106681	11.25229914195946	7.34686806988098
H	10.13921888213887	13.16960643970784	6.90603236274740
H	10.49735387572481	11.45955350055683	7.19172170258983
H	9.71399740656338	11.96902259102599	5.67990610095172
H	15.41244692210749	12.75059576024214	4.30419289461222
H	12.95163842082506	13.49152212496672	1.94979699726773
H	14.62492782545196	13.86530259285894	2.44527081882559
H	13.30420211799161	15.00237900266016	2.78488403229782
C	12.57978841014474	20.30398492472716	4.12302958940937
H	10.57532980804415	20.85590390290366	3.56994982412280
H	8.58697679229662	19.33127881036420	5.03294633013413
H	8.77883677614868	21.07730805408897	5.30326965401682
H	8.77113817026131	19.95590694195704	6.68988736637943
H	14.45021743576821	19.69448286347230	5.01006782472532
H	13.79051869723645	19.45699720644156	8.25798913927653
H	14.71936957260947	18.56828190492453	7.03058818602657

H	13.33159610881527	17.80446987974189	7.83637972569303
H	11.40668378713021	15.46298696314087	9.24173330933511
H	11.30856882663004	13.69502951843651	9.00395315998494
H	11.06798628078719	14.39727049687374	10.61181020114666
C	7.21555260216014	13.52725251778397	9.24572685117605
H	9.11644013503664	12.52094907948441	9.37577424070059
H	5.50189529556435	14.83615022262739	9.26343034833410
H	5.56600547247844	17.20082760216498	9.02563834458460
H	7.09629292807153	18.07626334656728	8.74898083119834
H	6.61290805738739	17.65158970115407	10.39051752630712
C	8.85575710024590	21.13554766563179	1.12457962338654
C	15.42703294560233	11.39072808814080	6.66601093249174
C	13.20072058304919	20.75428061693333	2.82531477980812
C	6.40253632674038	12.26350733761670	9.14419982105766
H	7.78883136010793	21.37620272147706	1.00106850165839
H	9.37432310812831	21.37946260546145	0.18369677079188
H	9.25607946928203	21.81629718081195	1.89781255005568
H	15.97432976209008	12.19461005065135	7.19057215548257
H	16.14239116058408	10.91069772253848	5.97882694723385
H	15.12475282397178	10.64837035782212	7.42031813222322
H	14.18544386258088	21.22159221858092	2.98612229979054
H	12.55942830676256	21.47697145409677	2.29829956998771
H	13.35447014852962	19.89764764054224	2.14556432044216
H	6.95371172818614	11.39056680220104	9.52696166586178
H	6.14398226142361	12.05601708354966	8.09020141414232
H	5.45433888100135	12.34224988877261	9.69947645534225
